# A New Customizable Surfactant LLPS Strategy for Sustainable and Highly Efficient Radioactive Metal Ion Separation

**DOI:** 10.1002/advs.75493

**Published:** 2026-05-18

**Authors:** Ruihan Yan, Yifu Hu, Wentao Wang, Zhi Cao, Meiwen Cao, Weifang Zheng, Guoan Ye, Taihong Yan

**Affiliations:** ^1^ Department of Radiochemistry China Institute of Atomic Energy Beijing China; ^2^ State Key Laboratory of Heavy Oil Processing & Department of Biological and Energy Chemical Engineering College of Chemical Engineering China University of Petroleum (East China) Qingdao China

**Keywords:** customizable, enhanced separation, liquid–liquid phase separation, radioactive metal ion separation

## Abstract

The traditional aqueous‐organic biphasic extraction for separating radioactive metal ions faces major challenges due to heavy reliance on volatile organic solvents and the resulting refractory radioactive waste. Herein, we propose a customizable, sustainable, and efficient separation strategy for radioactive ions by using liquid–liquid phase separation (LLPS) phenomenon in two coexisting aqueous phases. Unlike conventional organic extraction systems, the LLPS platform operates without forming a bulk organic extraction phase. The LLPS is formed by sodium dodecyl sulfate (SDS), cetyltrimethyl ammonium bromide (CTAB), and hexafluoroisopropyl alcohol (HFIP) (termed as SCH‐extraction system) and enables customized encapsulation of commercial extractants. The selectivity of SCH‐extraction system can be precisely tuned by adjusting parameters like the type of extractant and solution acidity, making it highly versatile for various separation scenarios. In simulated high‐level liquid waste (HLLW), the strategy achieved the separation factors of ${\mathrm{UO}}_{2}^{2+}$/Nd^3+^ reaching 2.33 × 10^4^, outperforming existing methods. This superior efficiency is driven by a dual mechanism: hydrophobic entrapment of metal‐extractant complexes and electrostatic attraction to the negatively charged condensate interface. Based on this, a thermodynamic model is proposed, providing rational design principles for optimizing the system for diverse separation applications. This strategy holds significant promise for separating valuable radioactive metal ions.

## Graphical abstract

A diluent‐free, customizable liquid–liquid phase separation (LLPS) platform enables highly efficient radioactive metal ion extraction and stripping. By encapsulating hydrophobic extractants into an ionic surfactant‐rich condensed phase, this LLPS system leverages synergistic electrostatic and coordination interactions. Consequently, it achieves excellent extraction efficiency and selectivity for radioactive metal‐ion separation.

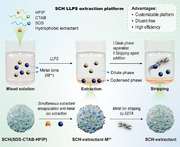

## 1 Introduction

The rapid expansion of nuclear energy and related technologies has intensified the global demand for efficient separation of radioactive isotopes (e.g., ^235^U, ^239^Pu, ^147^Pm, ^90^Sr) from complex waste streams, driven by both environmental safety imperatives and strategic resource recovery needs [1, 2]. Conventional liquid–liquid extraction (LLE) using aqueous‐organic biphasic systems, while widely adopted for its scalability and high separation efficiency for both laboratory‐scale experiments and large‐scale industrial applications. However, LLE heavily relies on large volumes of organic diluents as the continuous extraction phase to dissolve extractants, leading to significant volatile organic compounds (VOCs) emissions and the generation of substantial quantities of difficult‐to‐treat radioactive organic secondary waste, complicating waste management, causing radiolytic degradation issues, and posing safety operation risks [3]. Moreover, limitations in extractant solubility may lead to third‐phase formation, which further complicates the separation process [4]. These challenges underscore the urgent need for process sustainable extraction systems that eliminate organic diluent dependency.

Liquid–liquid phase separation (LLPS) [5, 6, 7] refers to the thermodynamic process where a single liquid phase spontaneously separates into two or more coexisting liquid phases with different compositions, which is often induced by heating (cloud‐point extraction (CPE) [8]) or inducing agents [9]. The novel extraction method based on LLPS, termed as LLPS‐extraction, as an emerging alternative, has shown promise in metal ion separation [10, 11, 12, 13, 14]. Nevertheless, existing LLPS platforms for metal ion separation are predominantly based on non‐ionic compounds (e.g., PEG, Triton‐114, L64) [15], where phase separation is commonly achieved by incorporating an additional polymer [16], salts [17, 18], or by controlling the temperature [19, 20]. *Non‐ionic compound‐based LLPS systems* suffer from critical limitations: (1) restricted solubilization capacity for hydrophobic organic ligands (<2 mmol·kg^−^^1^ 1‐(2‐pyridylazo)‐2‐naphthol (PAN) [21, 22]), which limits their utility to trace‐level separations; (2) high viscosity (>100 mPa·s) [23] prolongs phase separation and hinders mass transfer processes [15]; (3) high ionic strength (1–4 M) may disrupt intermolecular interactions and the phase separation process [18], consequently affecting metal ion separation efficiency and selectivity [15]; (4) phase instability under acidic conditions due to polymer protonation [8]; and (5) the need for energy‐intensive heating [8]. These constraints collectively restrict their viability for acidic, high concentration, or large‐scale radioactive metal ion recovery [24].

To overcome these barriers, *ionic surfactant‑based LLPS systems* offer an alternative. The diverse range of ionic surfactants [25, 26, 27] allows for targeted selection of phase‐forming molecules with excellent acid tolerance (e.g., quaternary ammonium salts), and eliminates the need for a large amount of salt or heating during LLPS. Besides, oppositely charged ionic surfactant condensates offer flexible control over encapsulation by precisely balancing coulombic attraction and short‐range repulsion, in addition to modulating hydrogen bonding and hydrophobic interactions [11], leading to diverse internal structures.

**FIGURE 1:**
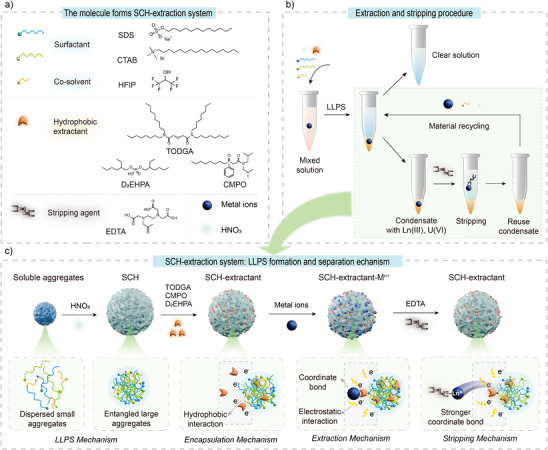
Overview of the SCH‐extraction system. (a) The molecules form SCH‐extraction system, including surfactants, extractants, and stripping agent. (b) Schematic illustration of the process of radioactive metal ion removal from aqueous solutions by the SCH‐extractant system and material recycling process. (c) Schematic of the SCH‐extraction system formation process and generation mechanism.

Herein, we introduce a customizable, sustainable, and highly efficient strategy for radioactive metal ion separation utilizing an innovative ionic surfactant‐based LLPS system in two coexisting aqueous phases. The LLPS system comprises of sodium dodecyl sulfate (SDS), cetyltrimethyl ammonium bromide (CTAB), and hexafluoroisopropyl alcohol (HFIP) (termed as SCH‐extraction system) (Figure 1). Notably, the SCH‐extraction system operates without a bulk organic diluent as the continuous extraction phase, with HFIP serving only as a phase‐modulating component and eliminating the need for salt addition or external heating to induce LLPS (Figure 1). Crucially, the molecular design flexibility of ionic surfactants within the SCH‐extraction system enables the hydrophobic microdomains with enhanced hydrophobicity and electrostatic tunability [28]. This allows for superior hydrophobic extractant solubilization, achieving solvation comparable to organic diluents and surpassing the solubilization capacity of non‐ionic compound‐based LLPS‐extraction systems (e.g., reaching a remarkable maximum surfactant‐to‐extractant molar concentration ratio of *C*_SDS+CTAB_: *C*_TODGA_ = 1:6.25). By selecting appropriate extractants and tuning parameters such as surfactant ratio and acidity, the SCH‐extraction system enables adaptable separation of radioactive metal ions and is validated in simulated high‐level liquid waste (HLLW), bauxite residues, and uranium mining effluents. Demonstrations in simulated HLLW showed ${\mathrm{UO}}_{2}^{2+}$/Nd^3+^ separation factors up to 2.33 × 10^4^, approximately 50 times higher than existing non‐ionic compound‐based LLPS extraction systems [29, 30, 31]. Therefore, the SCH‐extraction system presents a novel alternative for process sustainable nuclear waste management and critical metal recovery.

## 2 Results and Discussion

### 2.1 Phase Behavior, Structural Evolution, and Tunability of SCH‐extraction system

**FIGURE 2:**
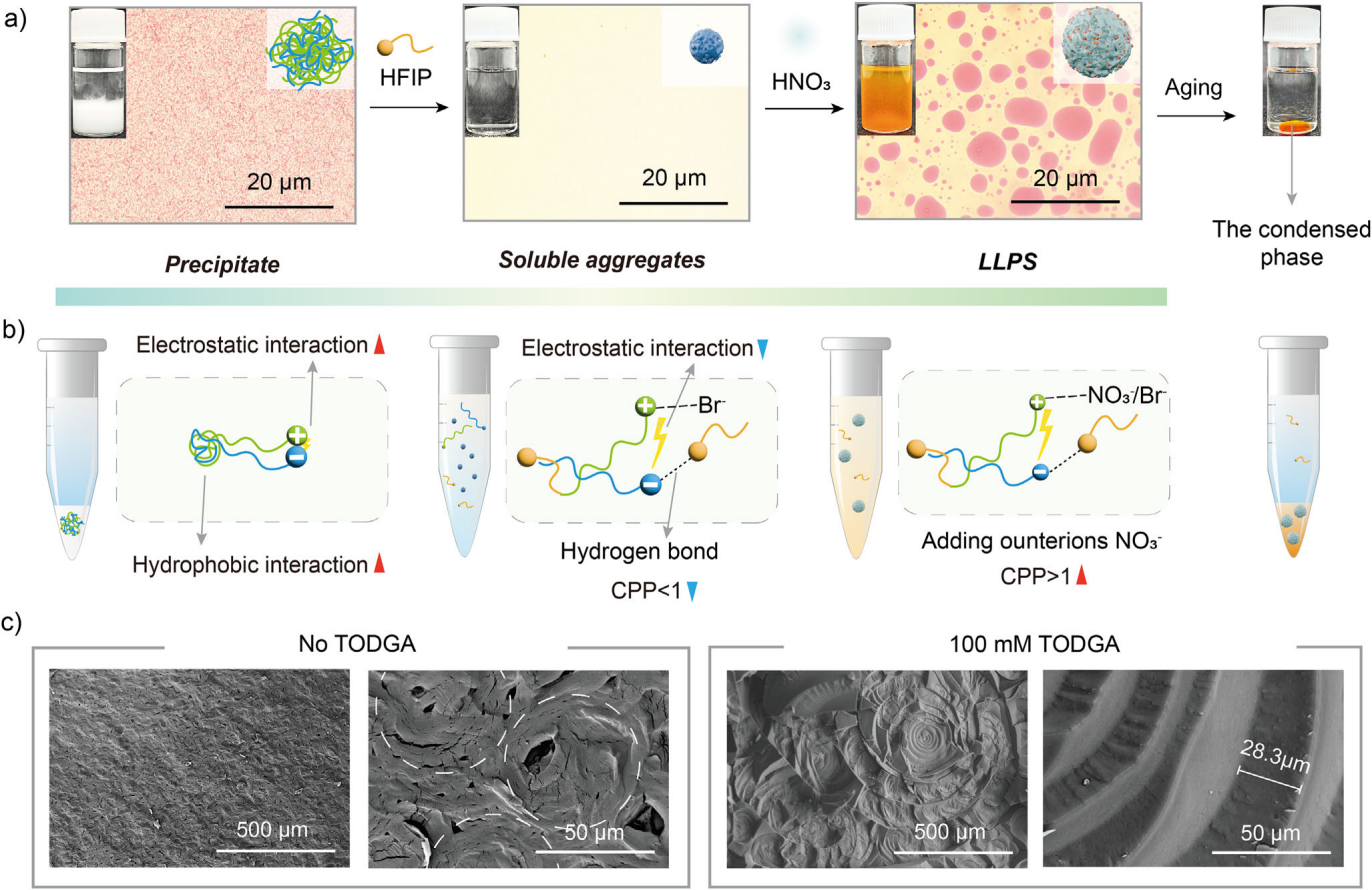
Phase behavior in SCH‐extraction system. (a) Phase transitions: from precipitation to soluble aggregates and macroscopic LLPS. (b) Schematic illustration of the evolution of intermolecular interactions during LLPS formation at the molecular‐level. c) Cryo‐SEM image of condensed droplets in the SCH system (*C_SDS_* = *C_CTAB_* = 40 mM, *C_HFIP_* = 4% v/v, $C_{HNO_{3}}=1\;M$, without extractant) and SCH‐TODGA system (*C_SDS_* = *C_CTAB_* = 40 mM, *C_HFIP_* = 4% v/v, $C_{HNO_{3}}=1\;M$, *C_TODGA_* = 100 mM).

The foundation of our novel LLPS‐extraction strategy lies in the controlled assembly of cationic surfactant CTAB and anionic surfactant SDS facilitated by HFIP. At an equimolar ratio (1:1), CTAB and SDS typically have strong electrostatic and hydrophobic interactions, forming precipitates (Figure 2a). Notably, the introduction of HFIP as a co‐solvent fundamentally alters this behavior. Owing to its strong hydrogen‐bond‐donating ability [32, 33], HFIP preferentially interacts with the sulfate headgroups of SDS, and forms localized solvation shells that attenuate the electrostatic attraction between oppositely charged surfactant headgroups. This hydrogen‐bond‐mediated solvation weakens the formation of rigid ion‐paired structures and promotes the transition from crystalline precipitates to fluidic aggregates (Figure 2b) [34]. In addition, the bulky CF_3_ groups of HFIP can perturb the hydrophobic packing of the alkyl chains by interacting with the terminal methyl groups of the alkyl chains. Inorganic counterions can trigger LLPS in various ionic surfactant systems [35]. ${\mathrm{NO}}_{3}^{-}$ acts as chaotropes with low hydration energy, which promotes the dehydration of CTA^+^ headgroups and effectively screens the electrostatic repulsion, and leads to a reduction in the effective headgroup area. According to the critical packing parameter (CPP) theory [36], this reduction causes the CPP to increase. This structural transition toward low‐curvature aggregates ultimately triggers the formation of the macroscopically separated LLPS. Such LLPS‐mediated reorganization, where weak hydrogen bonding, hydrophobic, and electrostatic interactions become spatially concentrated and cooperative within the condensed phase, is a general feature of LLPS‐driven self‐assembly processes, as extensively discussed in supramolecular systems [37]. Optical microscopy confirmed the LLPS process, revealing spherical condensed droplets (1–20 µm) in the solution (Figure 2a; Figure S1). In addition, the incorporation of the hydrophobic extractant TODGA markedly influenced the morphology of the condensed phase. Cryogenic scanning electron microscopy (cryo‐SEM) unveiled distinct structural differences. While the condensed phase of the SCH system exhibited irregular droplets, that of the SCH‐TODGA system displayed highly ordered, concentric layered structures reminiscent of cabbage leaves (Figure 2c). This striking transformation underscores the role of TODGA in promoting molecular ordering within a structured, water‐rich microenvironment in the condensed phase, leading to well‐defined supramolecular assemblies rather than forming a separate oil phase [37].

**FIGURE 3:**
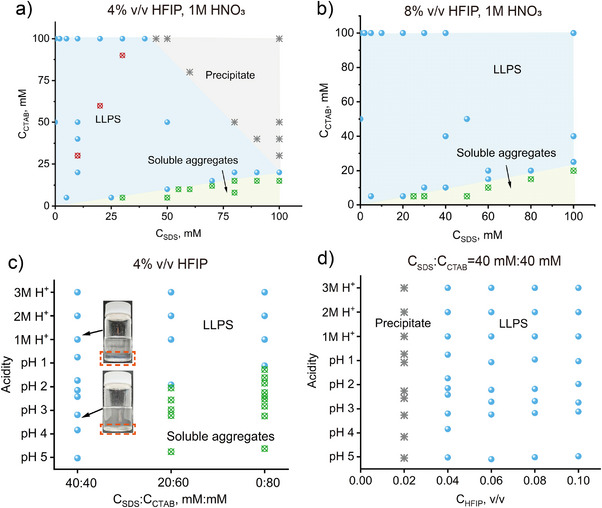
Phase diagram of SCH‐extraction system. (a) with 4%v/v HFIP at different *C_CTAB_* and *C_SDS_* (0–100 mM); (b) with 8%v/v HFIP at different *C_CTAB_* and *C_SDS_* (0–100 mM); (c) with 4%v/v HFIP at different surfactant ratios (*C_CTAB_*: *C_SDS_* = 40:40–0:80); d) with *C_CTAB_*: *C_SDS_* = 40:40 at different *C_HFIP_* (0.02–0.1). * The conditions corresponding to the red dots cannot form condensate.

To establish the operational window and tunability of the SCH LLPS system, the phase boundaries were systematically mapped as a function of key parameters (Figure 3). These phase diagrams were established using the base SCH system (without extractants) to define a conservative “boundary” for LLPS formation. The incorporation of the hydrophobic extractant may further stabilize the condensed phase and expand the LLPS region. Its structural evolution is governed by a delicate competition between electrostatic coupling, hydrophobic association, and steric hindrance. On the one hand, the hydrophobicity of TODGA lowers the threshold for LLPS. On the other hand, its bulky branched structure functions as a “molecular wedge” that disrupts the crystalline packing of the DS^−^‐CTA^+^ complex, effectively preventing ordered precipitation [38, 39]. In all investigated conditions, macroscopic phase separation was observed within several minutes (Figure S3), the detailed kinetic analysis of LLPS formation is discussed in Section S1 of Supporting Information. Representative phase diagrams reveal critical dependencies on surfactant stoichiometry, HFIP concentration (*C_HFIP_*), and counterion. First, at fixed total surfactant concentration (*C_SDS+CTAB_* ≤ 200 mM) and HFIP (4% v/v), LLPS formation is highly sensitive to the SDS/CTAB ratio. Ratios > 5:1 produce soluble aggregates (e.g., micelles, vesicles), while ratios < 5:1 promote aggregate growth and resulting LLPS. Notably, a 3:1 CTAB/SDS ratio only yielded minor turbidity, suggesting near‐monodisperse aggregates [40]. Excessively high concentrations of either surfactant (gray zones, Figure 3a) revert to precipitation due to overwhelming electrostatic and hydrophobic attractions. Second, *C_HFIP_* is important for stabilizing the condensed phase. At *C_HFIP_* = 2% v/v, HFIP is insufficient to fully prevent precipitation. However, at *C_HFIP_* ≥ 4% v/v, stable LLPS form across a wide range of surfactant concentrations and ratios (Figure 3a,b,d), demonstrating the dual function of HFIP as a co‐solvent and electrostatic modulator. Increasing *C_HFIP_* to 8% v/v expanded the LLPS region, suppressing precipitation even at higher surfactant loadings (Figure 3b vs Figure 3a). Third, the interplay between surfactant counterions (Br^−^ from CTAB, ${\mathrm{NO}}_{3}^{-}$ from HNO_3_, Na^+^ from SDS) profoundly impacts LLPS formation, especially under CTAB‐rich conditions (Figure 3c). When *C_CTAB_* > *C_SDS_*, the excess CTAB increases the electrostatic repulsion within the surfactant aggregates. Upon addition of HNO_3_, ${\mathrm{NO}}_{3}^{-}$ can induce the formation of low‐curvature aggregates by screening charges and reducing electrostatic repulsion between surfactant headgroups, allowing for tighter packing [41, 42], and thus LLPS.

Critically, the SCH system exhibits stability across a broad acidity spectrum, maintaining its integrity from 10^−5^ up to 3 M HNO_3_ (Figure 3d). This robust acid tolerance, a key limitation of non‐ionic compound‐based LLPS systems, positions the SCH platform as uniquely suited for the demanding conditions encountered in radioactive waste processing, such as high‐level liquid waste (HLLW).

### 2.2 Tunable Selectivity and Scalability in SCH‑Extraction System

The SCH‐extraction system distinguishes itself by tunable selectivity and scalable separation capabilities, which are critically influenced by the condensate's encapsulation capacity for various extractants and the stability at high metal ion concentrations. As evidenced in Figure S4, the system achieves high extractant solubility and robust stability against metal ion interference under conditions of *C_SDS_* = 40 mM, *C_CTAB_* = 40 mM, *C_HFIP_* = 8%v/v. Even when the extractant concentration was increased to 500 mM, and $C_{SDS+CTAB}\;:\;\;C_{M^{n+}}=4:1$, the system neither precipitated nor transitioned to a single phase [43], maintaining LLPS. Consequently, the SCH‐extraction system demonstrates robust solubilizing power and stability, enabling its application in the separation of high‐concentration metal ions.

The selectivity of the SCH‐extraction LLPS system for metal ions is a critical prerequisite for its application in metal ion separation and enrichment. While extractant determines the intrinsic coordination preference toward specific metal ions, the LLPS platform provides an extractant‐rich and low‐dielectric microdomain that amplifies this selectivity by thermodynamically favoring the partitioning of target metal‐ligand complexes into the condensed phase.

**FIGURE 4:**
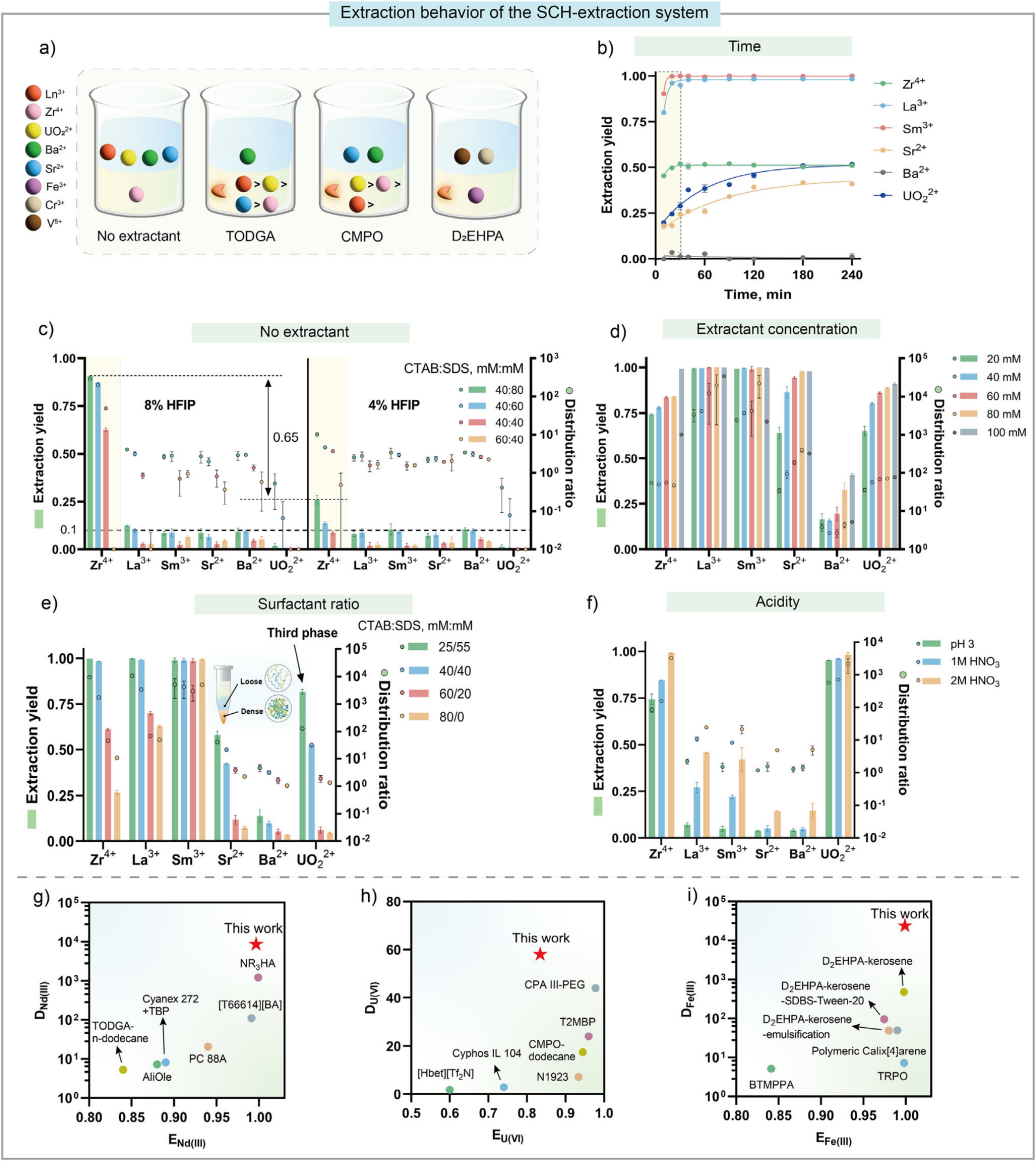
Separation behavior of metal ions in the SCH‐extraction system. (a) Schematic illustration of the selectivity of the SCH‐extraction system for metal ions in the absence and presence of TODGA, CMPO, and D_2_EHPA as extractants. (b) Extraction kinetics within 0–240 min (*C_SDS_* = *C_CTAB_* = 40 mM, *C_HFIP_* = 4% v/v, $C_{HNO_{3}}=1\;M$, *C_TODGA_* = 10 mM, $C_{M^{n+}}=40\;\mathrm{ppm}$). Equilibrium is considered as the condition where the rate of change of the extraction rate approaches 0.01 h^−1^). c) Selectivity of metal ions in SCH‐extraction system without extractants (*C_HFIP_* = 4, 8% v/v, *C_SDS_* = *C_CTAB_* = 40 mM, $C_{HNO_{3}}=1\;M$, $C_{M^{n+}}=40\;\mathrm{ppm}$). (d) Effect of extractant concentration on the extraction yield and distribution ratio of major metal ions in simulated HLLW. (*C_TODGA_* = 20, 40, 60, 80, 100 mM, *C_SDS_* = *C_CTAB_* = 40 mM, *C_HFIP_* = 4% v/v, $C_{HNO_{3}}=1\;M$, $C_{M^{n+}}=40\;\mathrm{ppm}$). (e) Effect of surfactant ratio on the extraction yield and distribution ratio (*C_CTAB_*: *C_SDS_* = 25:55, 40:40, 60:20, 80:0, *C_HFIP_* = 4% v/v, $C_{HNO_{3}}=1\;M$, $C_{M^{n+}}=40\;\mathrm{ppm}$, *C_TODGA_* = 10 mM). (f) Effect of acidity on the extraction yield and distribution ratio ($C_{HNO_{3}}=10^{-3},\;1,\;2\;M$, *C_SDS_* = *C_CTAB_* = 40 mM, *C_HFIP_* = 4% v/v, *C_CMPO_* = 10 mM, $C_{M^{n+}}=40\;\mathrm{ppm}$). (g–i) Extraction yield and distribution ratio of Nd^3+^, ${\mathrm{UO}}_{2}^{2+}$, and Fe^3+^ in the literature and this study.

As shown in Figure 4a, the selectivity of the system was evaluated for major metal ions in simulated spent fuel. The selectivity is influenced by factors such as reaction time, type of extractant, extractant concentration, surfactant ratio, and acidity.

Regarding extraction kinetics and equilibrium, detailed kinetic studies of Zr^4+^, La^3+^, Sm^3+^, Sr^2+^, Ba^2+^, and ${\mathrm{UO}}_{2}^{2+}$ in the SCH‐TODGA system (1 M HNO_3_) revealed distinct extraction yields (Figure 4b). Sm^3+^ reached equilibrium within approximately 30 min, while Sr^2+^ and ${\mathrm{UO}}_{2}^{2+}$ required about 3–4 h. This variation arises primarily from multi‐stage kinetic constraints. Specifically, the prolonged 3‐h equilibration for Sr^2+^ and ${\mathrm{UO}}_{2}^{2+}$ is attributed to (i) effective diffusion resistance within the viscous condensed phase [44], where the “cabbage‐like” multi‐lamellar ordered morphology creates limited path for ion transport; (ii) significant desolvation energy barriers, as the transition of these high‐valent ions from the bulk dilute phase to the dense condensed phase requires overcoming substantial hydration forces [38]; (iii) transfer across the condensate‐water interface; and (iv) the time required for structural reorganization within the LLPS system [37] leading to optimal coordination geometry.

Notably, in the absence of any extractant, minimal extraction was observed for most metal ions, except for Zr^4+^ (Figure 4c), possibly owing to excluded volume effects [45]. Interestingly, Zr^4+^ was preferentially extracted, following the Hofmeister series of cations [35]. Its extraction efficiency decreased with increasing CTAB content and reducing HFIP concentration, consistent with weakened electrostatic attraction to cationic metals under conditions of diminished condensate surface negative charge.

Further insights into the influence of extractant concentration were gained from systematic evaluation of the SCH‐TODGA system (20–100 mM extractant), which confirmed a direct positive correlation between extractant loading and the distribution of metal ions into the condensed phase (Figure 4d; Figure S5a). For instance, at 20 mM TODGA, trivalent lanthanides achieved ∼100% extraction yield, while Sr^2+^, Zr^4+^, and ${\mathrm{UO}}_{2}^{2+}$ showed 70%–80% extraction yields. The extraction yield of Zr^4+^ was enhanced by ≥ 70% in the SCH‐TODGA system compared to the SCH system alone (Figure 4c).

Furthermore, optimizing the surfactant ratio proved critical. Increasing the SDS proportion at fixed total surfactant concentrations (*C_CTAB_*: *C_SDS_* = 80:0 → 25:55) markedly elevated the condensate's negative charge density, consequently enhancing metal ion extraction yield (Figure 4e), reflecting a reorganization of aggregate structure during LLPS. Lanthanides maintained > 60% extraction yield even under CTAB excess conditions (≥ 60 mM). However, an excessively high SDS ratio (e.g., *C_CTAB_*: *C_SDS_* = 1:3) induces pronounced structural and interfacial imbalance, disrupting phase separation or led to the formation of undesirable third phases of coexisting aggregate, complicating the separation process (Figure 3a,b).

Acidity plays a crucial role in modulating both metal complex stability and extractant structure within the condensate (Figure 4f; Figure S5b) [46]. In SCH‐CMPO systems, Ln^3+^ extraction yields decreased below pH 3, even reaching 0 at 10 mM CMPO. Conversely, ${\mathrm{UO}}_{2}^{2+}$ maintained high extraction yields (> 95%) from 10^−3^ ∼ 2 M HNO_3_ (Figure 4f). This differential behavior facilitates single‐stage Ln(III)/U(VI) separation under low acidity conditions, a highly advantageous feature for selective recovery.

The SCH‐extraction system consistently demonstrated superior metal ion extraction performance and versatility in a single cycle compared to previously reported methods, as evidenced by enhanced extraction yield and distribution ratio (Figure 4g–i, Tables S1–S3). Under highly acidic conditions (pH < 2), the SCH‐based LLPS system exhibited distribution ratios for Ln(III) that were 3 orders of magnitude higher than those obtained using the same extractant dissolved in conventional *n*‐dodecane (*D* = 10^3^–10^5^ vs 10^0^–10^2^, Figure S6a) [46]. A similar amplification effect was observed for Fe(III), where distribution ratios increased by approximately 4 orders of magnitude (Figure S6c) [47]. Importantly, this difference does not arise from a change in the intrinsic coordination preference of the extractant, but from the distinct physicochemical environment provided by the LLPS condensates. In the SCH system, extractants are highly enriched within the condensed phase, resulting in substantially elevated local extractant concentrations compared with homogeneous organic solvent systems. Meanwhile, the condensed phase constitutes a water‐restricted, intermediate‐dielectric microenvironment formed by surfactant headgroups, alkyl chains, and HFIP, which weakens metal hydration and enhances metal‐extractant coordination. These combined effects suppress competitive hydration and facilitate complexation of metal ions, thereby amplifying the apparent distribution ratios relative to conventional organic solvent extraction.

**FIGURE 5:**
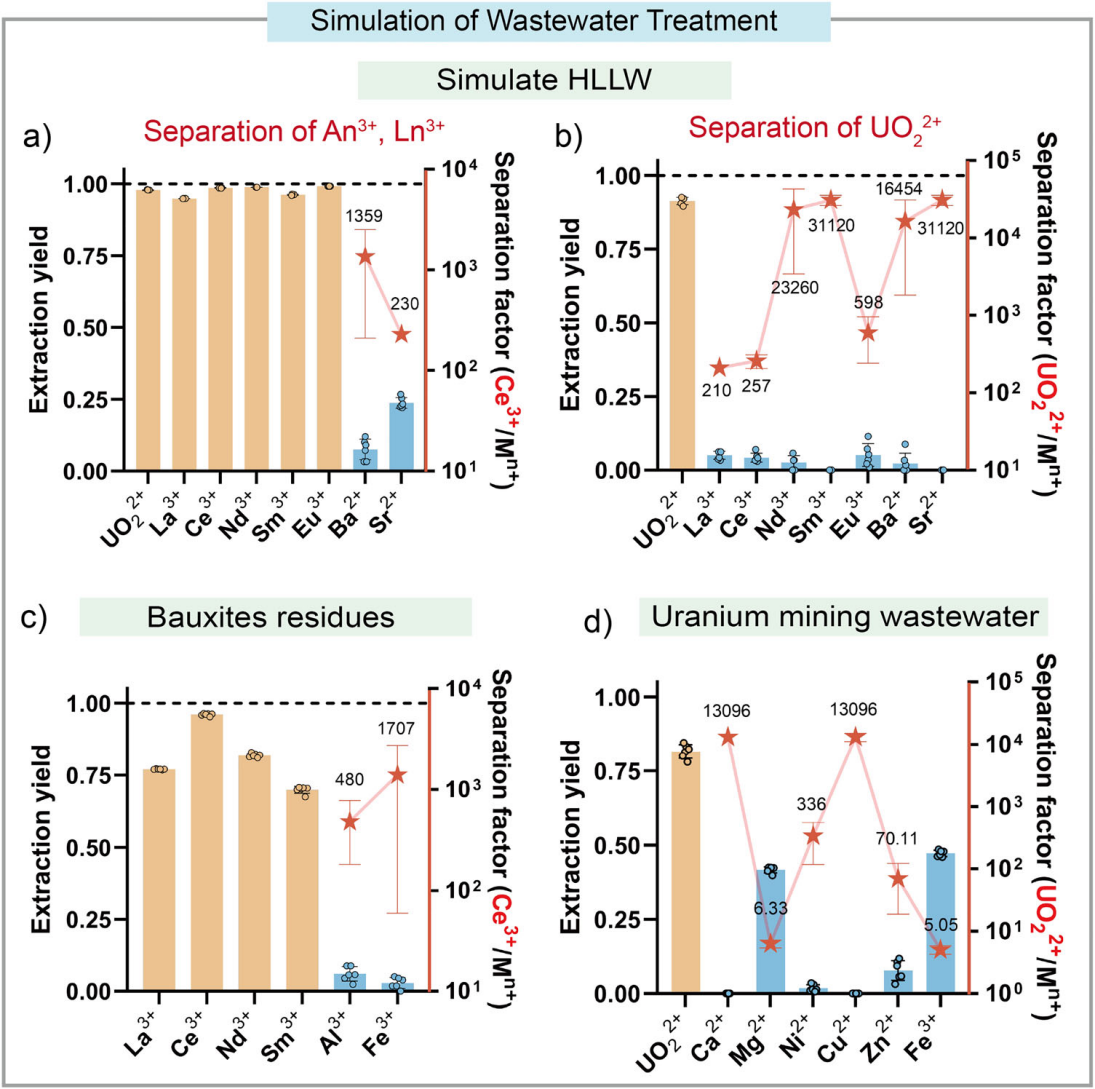
Separation behavior of metal ions in various simulated wastewater. (a) HLLW (*C_TODGA_* = *C_CMPO_* = 10 mM, $C_{HNO_{3}}=1\;M$); (b) HLLW (*C_CMPO_* = 10 mM, $C_{HNO_{3}}\;=10^{-3}\;M$); (c) Bauxites residues (*C_TODGA_* = 10 mM, $C_{HNO_{3}}=1\;M$); c) Acid uranium mining effluents (*C_CMPO_* = 10 mM, $C_{HNO_{3}}=1\;M$) treated by SCH‐extraction system.

In addition, the SCH‐extraction system exhibits broad versatility in diverse wastewater treatment applications (Figure 5 and Table 1). The separation of radioactive ions was simulated with high‐level liquid wastes (HLLW), bauxite slag wastewater [48], acidic uranium ore wastewater [49], and vanadium‐titanium‐magnetite wastewater [50]. For HLLW primarily containing lanthanides, actinides, and alkaline earth metals (Figure S7a), excellent group separation was achieved by SCH‐extraction system. The SCH‐TODGA+CMPO system enabled the group separation of U(VI) and Ln(III) from divalent alkaline earth metal ions, with a separation factor (*SF*) for Ce^3+^ relative to other ions reaching a maximum of $SF_{Ce^{3+}/Ba^{2+}}=1.36\;\times \;10^{3}$ (Figure 5a). In contrast, the SCH‐CMPO system facilitated the group separation of U(VI) from both Ln(III) and divalent alkaline earth metal ions. Specifically, at pH 3, this system exhibited a remarkably high separation factor of $SF_{{\mathrm{UO}}_{2}^{2+}/Sr^{2+}}=3.11\;\times \;10^{4}$, $SF_{{\mathrm{UO}}_{2}^{2+}/Nd^{3+}}=2.33\;\times \;10^{4}$(Figure 5a), which is approximately 50 times greater than that reported for comparable separation systems [29, 30, 31]. For Greek bauxite residue (red mud) (Figure S7b) [48], the SCH‐TODGA system achieved approximately 80% average extraction yield of Ln(III). For Ce^3+^ separation from other elements, notably Fe^3+^ (high content in ore), separation factors reached $SF_{Ce^{3+}/Fe^{3+}}=1.71\;\times \;10^{3}$ (Figure 5c), demonstrating excellent performance for challenging separations. In the context of radioactive uranium decontamination from simulated uranium tailings wastewater (1 M HNO_3_) (Figure S7c) [49], the SCH‐CMPO system achieved approximately 82% U(VI) extraction in a single stage (Figure 5d). U(VI) exhibited substantial separation factors from the heavy metal ions, with $SF_{{\mathrm{UO}}_{2}^{2+}/Cu^{2+}}=1.31\;\times \;10^{4}$. Finally, for vanadium and titanium magnetite resources (Figure S7d) [50], SCH‐D_2_EHPA system allowed for effective separation of Fe^3+^ from ${\mathrm{VO}}_{2}^{+}$ and Cr^3+^ at pH 1, with separation factors of $SF_{Fe^{3+}{/\mathrm{VO}}_{2}^{+}}=4.26\;\times \;10^{3}$ and $SF_{Fe^{3+}/Cr^{3+}}=6.04\;\times \;10^{2}$ respectively (Figure S7e). These examples collectively demonstrate the versatility and high efficiency of the SCH‐extraction system across various separation scenarios, highlighting its potential as a promising separation strategy.

**TABLE 1:** The separation factors of the target metal ions from other metal ions in Figure 5.

Separation factor in Figure 5a						
Ce^3+^/Ba^2+^	Ce^3+^/Sr^2+^					
1.36 × 10^3^	2.30 × 10^2^					

Separation factor in Figure 5b						
${\mathrm{UO}}_{2}^{2+}/La^{3+}$	${\mathrm{UO}}_{2}^{2+}/Ce^{3+}$	${\mathrm{UO}}_{2}^{2+}/Nd^{3+}$	${\mathrm{UO}}_{2}^{2+}/Sm^{3+}$	${\mathrm{UO}}_{2}^{2+}/Eu^{3+}$	${\mathrm{UO}}_{2}^{2+}/Ba^{2+}$	${\mathrm{UO}}_{2}^{2+}/Sr^{2+}$
2.10 × 10^2^	2.60 × 10^2^	2.33 × 10^4^	3.11 × 10^4^	5.98 × 10^2^	1.65 × 10^4^	3.11 × 10^4^

Separation factor in Figure 5c						
Ce^3+^/Al^3+^	Ce^3+^/Fe^3+^					
4.80 × 10^2^	1.71 × 10^3^					

Separation factor in Figure 5d						
${\mathrm{UO}}_{2}^{2+}/Ca^{2+}$	${\mathrm{UO}}_{2}^{2+}/Mg^{2+}$	${\mathrm{UO}}_{2}^{2+}/Ni^{2+}$	${\mathrm{UO}}_{2}^{2+}/Cu^{2+}$	${\mathrm{UO}}_{2}^{2+}/Zn^{2+}$	${\mathrm{UO}}_{2}^{2+}/Fe^{3+}$	
1.31 × 10^4^	6.33	3.36 × 10^2^	1.31 × 10^4^	70.11	5.05	

### 2.3 Mechanism of Extractants Encapsulation and Dual‐Mechanism Extraction

**FIGURE 6:**
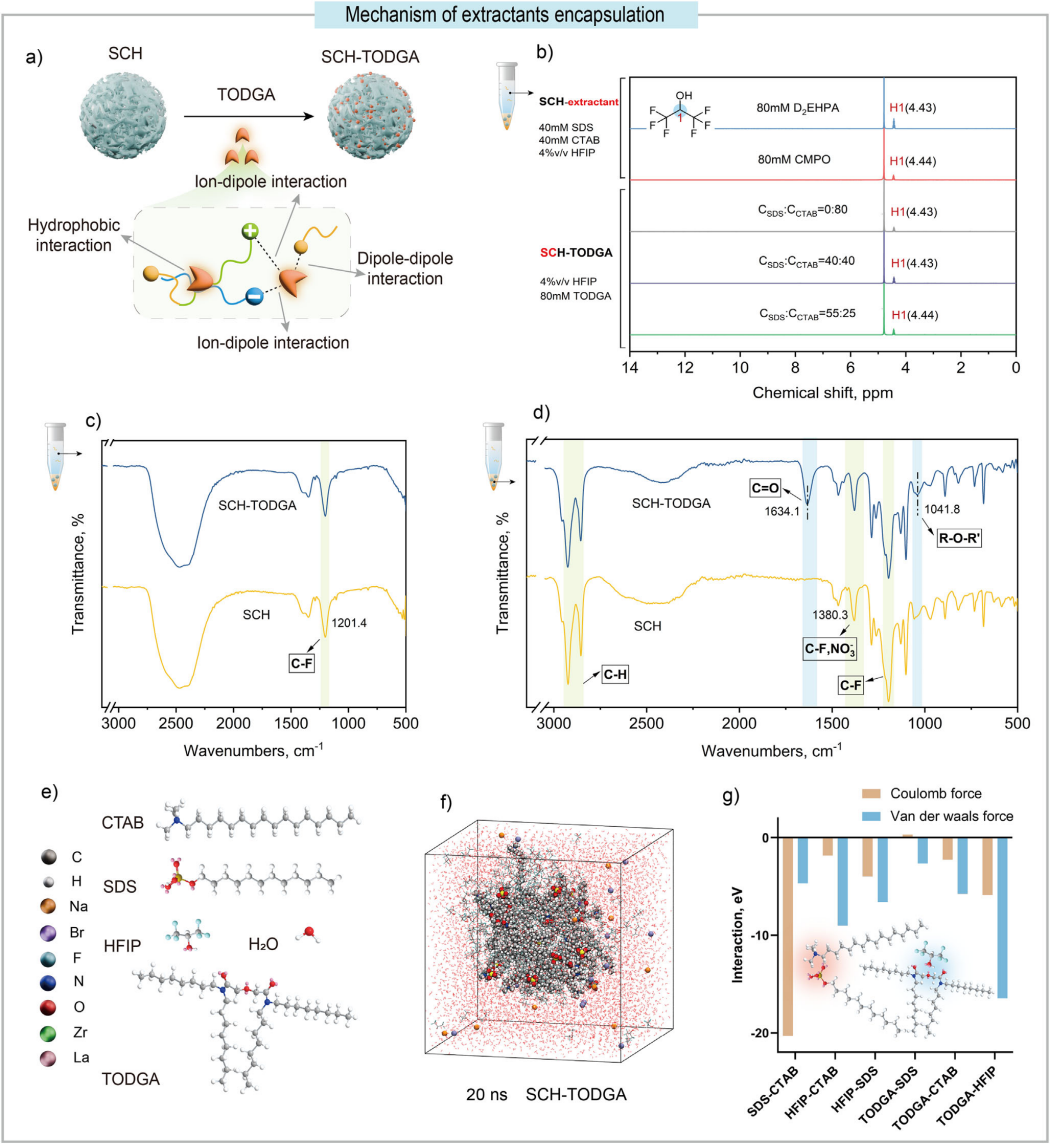
Mechanism of extractants encapsulation by the SCH system. (a) Schematic illustration of extractant encapsulation within the SCH‐extraction system. (b) ^1^H nuclear magnetic resonance spectrum of the dilute phase in the SCH‐TODGA/CMPO/D_2_EHPA system in D_2_O at 25°C. FT‐IR spectra of SCH‐TODGA system in D_2_O (*C_SDS_* = *C_CTAB_* = 40 mM, *C_HFIP_* = 4% v/v, *C_TODGA_* = 40 mM, $C_{\mathrm{HN}O_{3}}=1\;M$) (c) of the dilute phase; (d) of the condensed phase. (e) Molecular model of SDS, CTAB, HFIP, TODGA, Zr, La, H_2_O. (f) Typical snapshots from MD simulations among SDS, CTAB, HFIP, and TODGA in the SCH‐TODGA system over 20 ns at 298 K in a 10 × 10 × 10 nm^3^ volume. (g) Value of non‐bonded interaction energy (Coulomb force and Van der Waals force) between molecules in SCH‐TODGA system. Orange represents electrostatic interaction, while Blue represents Van der Waals interaction.

Metal ion separation efficacy depends on extractant encapsulation within the condensed phase, governed by host‐guest complexation. This encapsulation is driven by synergistic hydrophobic, electrostatic, and hydrogen‐bonding interactions, which determine encapsulation capacity and stability (Figure 6a). To comprehensively evaluate this behavior, we employed both experimental and computational approaches.

Experimental evidence for extractant encapsulation was primarily obtained through ^1^H NMR spectroscopy and FT‐IR analysis of the dilute phase. ^1^H NMR spectroscopy of the dilute phase revealed that HFIP remained the predominant solute, evidenced by a characteristic septet peak at 4.40–4.43 ppm corresponding to its H1 proton (Figure 6b). The absence of extractant, SDS, and CTAB signals (Figure S8) confirms their nearly complete partitioning into the condensed phase. Consistently, total organic carbon (TOC) measurements of the dilute phase show low organic content that does not increase upon extractant addition (Figure S13b), confirming that the residual organic carbon originates primarily from HFIP rather than from extractant or surfactant. A slight shift in the H1 peak with varying solute concentrations (discussed in detail in Supporting Information) suggests subtle alterations in the microenvironment to HFIP within the LLPS system, leading to slight shielding or deshielding effects on its protons [51, 52, 53]. Complementary FT‐IR analysis of the dilute phase further supported these conclusions. No discernible absorption band shifts were observed before and after TODGA addition (Figure 6c; Figure S11). Apart from a sharp peak at 1201.4 cm^−1^ of C─F stretching vibration of HFIP and the peaks at 1348.1 and 1393.8 cm^−1^ that arise from overlapping of C − F and $-{\mathrm{NO}}_{3}^{-}$ groups’ asymmetric stretching vibration [40, 54], no typical peaks of TODGA were detected, signifying the predominant encapsulation of TODGA within the condensed phase. This exclusion of TODGA from the dilute phase is consistent with its stabilization within the condensates via ion‐dipole interactions between the highly polar C═O groups of TODGA and the charged surfactant headgroups, which suppresses its solvation by bulk water.

To further characterize the distribution of components, FT‐IR analysis was conducted on the condensed phase of both the pristine SCH‐extraction system without extractant and SCH‐extractant systems. Peaks at 2925.1 and 2855.4 cm^−1^ indicated C─H stretching vibrations, while the peak at 1195.6 cm^−^^1^ corresponded to the C─F stretching vibration (Figure 6d) [55]. In the dilute phase, the C─F stretching vibration peak at 1201.3 cm^−1^ (Figure S11a) exhibited a slight blue shift and significantly lower intensity. These observations confirm that HFIP is distributed in both the condensed and the dilute phases, with a notably higher concentration within the condensed phase, consistent with the NMR findings (Figure 6c). Upon TODGA addition to the SCH system (Figure 6d), a prominent peak at 1634.1 cm^−1^ appeared, primarily attributed to the C═O stretching vibration with a minor contribution from C─N [56]. Concurrently, an R‐O‐R’ band at 1041.8 cm^−1^ further confirmed the presence of TODGA. Similar spectral features, such as a broad C═O stretching vibration band at 1615.8 cm^−1^ and a P═O stretching vibration band at 1041.2 cm^−1^ [57], were observed for SCH‐CMPO system (Figure S12a), characteristic of CMPO. In SCH‐D_2_EHPA system (Figure S12b), a broad peak at 1027.7 cm^−1^ likely resulted from the overlap of the P═O stretching vibration with P‐O‐H and P‐O‐C stretching vibrations [58], indicating the presence of D_2_EHPA. Collectively, these FT‐IR results unequivocally demonstrate the efficient encapsulation of hydrophobic extractants within the condensed phase. At the molecular level, the stable incorporation of extractants within the SCH condensates can be rationalized by a cooperative network of noncovalent interactions. The oppositely charged surfactant headgroups in the condensed phase generate locally intense electrostatic fields, which are expected to favor ion‐dipole interactions with the highly polar functional groups of the extractants (e.g., C═O or P═O). In addition, dipole‐dipole coupling among adjacent extractant molecules becomes increasingly significant in the relatively low‐dielectric microenvironment of the condensates, facilitating their local enrichment without inducing macroscopic phase separation. Van der Waals interactions between the alkyl chains of extractants and surfactants ensure hydrophobic compatibility, embedding extractants within microdomains without forming a separate oil phase. Collectively, these multivalent interactions provide a consistent molecular‐level explanation for the efficient and stable encapsulation of hydrophobic extractants within the SCH‐based LLPS system. Notably, no macroscopic oil phase or emulsion‐like turbidity was observed upon TODGA addition (Figure S4). Combined with the absence of TODGA signals in the dilute phase, the Bingham‐like rheological behavior (Figure S2), and the highly structured morphology of the condensed phase revealed by cryo‐SEM (Figure 2c), these results indicate that TODGA is partitioned into the surfactant‐rich LLPS condensates rather than forming a separate organic oil phase.

To elucidate the mechanism of extractant encapsulation, molecular dynamics (MD) simulations were performed on the SCH‐TODGA system (Figure 6e). By 20 ns, SDS, CTAB, and TODGA molecules clustered into condensates (Figure 6f), consistent with experimental data (Figure 6b,c). Analysis of non‐bonded potential energy, encompassing electrostatic and van der Waals interactions (Figure 6g), revealed a rapid association between SDS and CTAB (−20.32 eV), primarily driven by coulombic attraction. TODGA encapsulation was driven by hydrophobic interactions [59], evidenced by van der Waals attraction between TODGA and HFIP (−16.45 eV) or surfactant alkyl chains (SDS: −2.64 eV; CTAB: −5.76 eV). Furthermore, ion‐dipole interaction (e.g., −2.27 eV between TODGA and CTAB), and dipole‐dipole interactions between TODGA and HFIP, also stabilize the encapsulation.

Metal ion separation in the SCH‐extraction system occurs via two primary mechanisms: hydrophobic encapsulation of metal‐extractant complexes and electrostatic attraction toward the negatively charged condensate interface. These mechanisms operate independently or synergistically; here, we examine Zr^4+^ in the SCH system (electrostatic attraction), and La^3+^ in the SCH‐TODGA system (synergistic mechanism) to elucidate the underlying processes.

**FIGURE 7:**
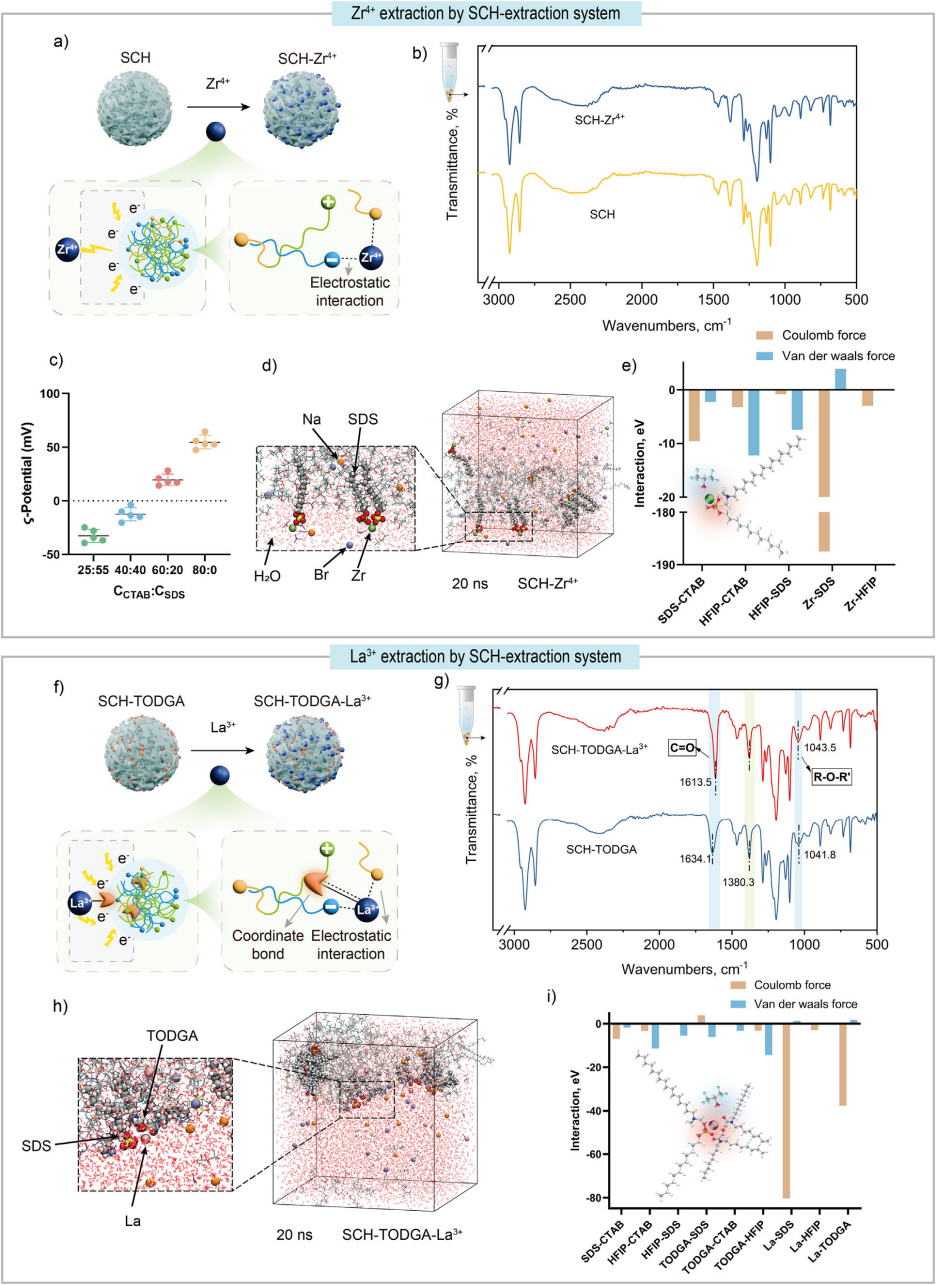
Dual‐mechanism of metal ions separation by the SCH‐extraction system. (a) Schematic of Zr^4+^ extraction by the SCH‐extraction system without extractant. Only the primary ions and molecules participating in Zr^4+^ ion coordination are illustrated, excluding other components like ${\mathrm{NO}}_{3}^{-}$ (same as follows). (b) FT‐IR spectra of the SCH‐extraction system in D_2_O before and after Zr^4+^ addition (*C_SDS_* = *C_CTAB_* = 40 mM, *C_HFIP_* = 4% v/v, $C_{Zr^{4+}}=10\;\mathrm{mM}$, $C_{HNO_{3}}=1\;M$). (c) Apparent zeta potential of SCH‐extraction system. *C_CTAB_*: *C_SDS_* (mM:mM) = 2.5:5.5, 4:4, 6:2, 8:0. (d) Typical snapshots from MD simulations among SDS, CTAB, HFIP, and Zr(NO_3_)_4_ at 298 K and 20 ns. (e) Intermolecular non‐bonded interaction energies in the SCH‐Zr^4+^ system. (f) Schematic illustration of La^3+^ extraction by the SCH‐extraction system. (g) FT‐IR spectra of SCH‐TODGA system in D_2_O before and after La^3+^ addition (*C_SDS_* = *C_CTAB_* = 40 mM, *C_HFIP_* = 4% v/v, *C_TODGA_* = 40 mM, $C_{La^{3+}}\;=10\;\mathrm{mM}$, $C_{HNO_{3}}=1\;M$). (h) Typical snapshots from MD simulations among SDS, CTAB, HFIP, TODGA, and La(NO_3_)_3_. i) Intermolecular non‐bonded interaction energies in SCH‐TODGA‐La^3+^ system.

For Zr^4+^ extraction, the primary association with the SCH system occurs through electrostatic interactions (Figure 7a). FT‐IR analysis of the condensed phase in the SCH system showed no significant changes in wavenumber or peak intensities of C─H, C─F, and ${\mathrm{NO}}_{3}^{-}$ functional groups before and after Zr^4+^ addition (Figure 7b), indicating an absence of coordinate bonding between SCH components and Zr^4+^. Zeta potential analysis revealed that the SCH condensate surface carries negative charges when *C_SDS_*: *C_CTAB_* ≥ 1, with increasing SDS proportion that led to a more negative zeta potential (Figure 7c). MD simulations were employed to study the coordination of Zr^4+^ with SDS and HFIP within the SCH‐Zr^4+^ system ($C_{SDS}:C_{Zr^{4+}}=1:1$) (Figure 7d). At 20 ns, interactions between some Zr^4+^ and HFIP were observed in the dilute phase, followed by a gradual migration to the condensed phase as the simulation progressed. Partial retention of Zr^4+^ in the aqueous phase is observed, reflecting its strong hydration, limited dehydration, and limited electrostatic interaction in the absence of extractants. Energy calculations (Figure 7e) showed strong electrostatic attraction between Zr^4+^ and SDS (−187.45 eV), with HFIP (−2.97 eV) as well. These results indicate that Zr^4+^ extraction is driven by electrostatic attraction with the negatively charged interface. Additionally, Figure S14 reveals that the SCH‐Zr^4+^ condensate is less dense than the SCH‐TODGA condensate. This may be attributed to decreased ordering within the condensate structure due to the shielding effect of high‐valence salt (Figure 2c) [60]. Thus, the addition of hydrophobic extractants serves a dual function: enhancing metal ion selectivity via coordination and promoting the formation of a more ordered condensate.

In contrast, La^3+^ extraction by the SCH‐TODGA system follows a synergistic mechanism involving both hydrophobic and electrostatic pathways (Figure 7f). FT‐IR analysis of functional group variations before and after La^3+^ addition (Figure 7g) showed that the asymmetric stretching vibration of ${\mathrm{NO}}_{3}^{-}$ at 1380.3 cm^−1^ [54] remained unchanged in both wavenumber and peak type, suggesting minimal involvement of ${\mathrm{NO}}_{3}^{-}$ in metal coordination. However, upon La^3+^ addition, the C═O stretching vibration peak red‐shifted from 1634.1 to 1613.5 cm^−1^ [61], indicating coordination between the C═O group and La^3+^. Similarly, for ${\mathrm{UO}}_{2}^{2+}$ extraction by the SCH‐CMPO system, the C═O stretching vibration peak red‐shifted from 1615.8 cm^−1^ to 1579.5 cm^−1^, and the P═O stretching vibration peak nearly disappeared (Figure S15a), confirming involvement of both C═O and P═O in ${\mathrm{UO}}_{2}^{2+}$ coordination. The obvious reduction in the P═O stretching vibration peak upon Fe^3+^ addition further corroborated its involvement in coordination (Figure S15b). These spectroscopic results demonstrate that extractant coordination is essential for the synergistic mechanism.

Further supporting the synergistic separation mechanism for La^3+^ in the SCH‐TODGA system, MD simulations of SCH‑TODGA‐La^3+^ system ($C_{\mathrm{TODGA}}:C_{La^{3+}}=1:1$) confirmed that La^3+^ is primarily extracted into the condensed phase (Figure 7h). Non‐bonded potential energy calculations (Figure 7i) revealed dominant electrostatic attractions between La^3+^ and SDS (−80.36 eV), TODGA (−37.67 eV), and HFIP (−2.98 eV). The culmination of these findings (Figure 7g,i) highlights a dual mechanism governing metal ions extraction in the SCH‐extraction system: 1) Hydrophobic encapsulation, where metal ions coordinate with TODGA to form hydrophobic complexes [62], thereby promoting their transfer into the hydrophobic microdomains of the condensed phase; 2) Electrostatic interaction, where coulombic attraction between La(NO_3_)_3_ and negatively charged condensate interface drives selective ion accumulation within the condensate. The synergy between these hydrophobic encapsulation and electrostatic interactions underpins the high separation efficiency observed for the SCH‐extraction system. Collectively, these interactions substantially enhance the extraction yield. A generalized reaction mechanism equation (Equation (1)) has been proposed to describe this comprehensive process.

(1)

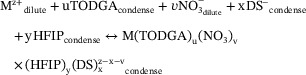




**FIGURE 8:**
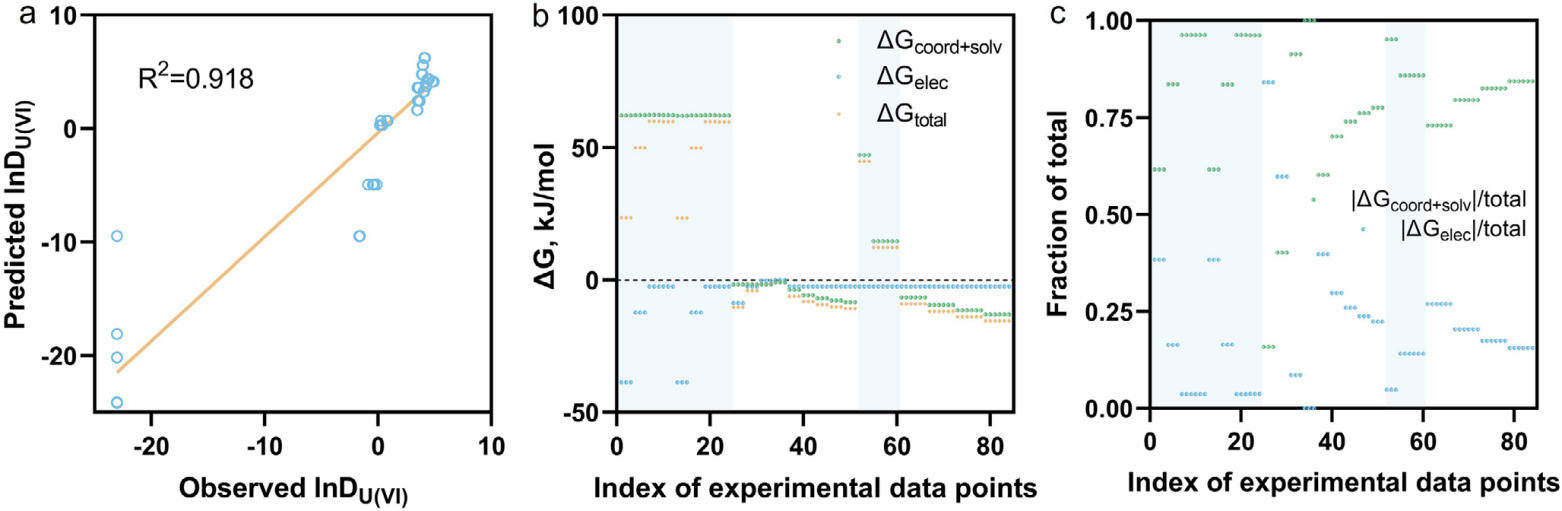
(a) Parity plot comparing experimentally observed lnD_U(VI)_ values (horizontal axis) with those calculated from the fitted model (vertical axis). (b) Total free energy contributions and (c) fractional contributions of the electrostatic, coordination, and solvation free energy. The horizontal axis in (b,c) represents the specific index of the experimental data points (obtained under varying extraction conditions, such as different surfactant ratios and acidities) used for model fitting. *total = |Δ*G_elec_* |+|Δ*G_coord_* |+|Δ*G_solv_* |.

Experimental results indicate that metal ions are predominantly extracted by the SCH‐extraction system via a dual‐mechanism. To further quantify and distinguish these mechanisms, we developed a thermodynamic model that decomposes the total Gibbs free energy change (Δ*G_total_*) into three contributions: electrostatic interactions (Δ*G_elec_*), coordination with extractants (Δ*G_coord_*), and solvent effects (Δ*G_solv_*) (Equation (2)). Equation (3) was employed to connect the distribution ratio to the free energy terms, allowing parameterization against the experimental dataset. In our model, the total free energy is determined primarily by the metal‐ion charge in the condensed phase, the SDS concentration that strongly attracts the cation electrostatically, the ionic strength, the concentration of HFIP, ${\mathrm{NO}}_{3}^{-}$, and the extractant (Equation (4)). To keep the model concise, variables that vary during diffusion and interfacial processes, such as dielectric constant and entropy changes, are absorbed into the constants c or A. Furthermore, non‐electrostatic interaction such as desolvation of metal ion and ligand coordination is regarded as coupled processes. Therefore, their combined free energy contributions are treated as a unified term (Δ*G_coord+solv_*) for fitting purposes. The desolvation penalties of metal ions are mathematically incorporated into an apparent coordination term *c* [63]. Only the most influential parameters are retained, ensuring both numerical stability and physical transparency; the full derivation is provided in Section S6 part of Supporting Information. For numerical stability during the non‐linear regression, the concentrations of SDS ([*SDS*]) and ionic strength (*I*) were scaled by their respective median values (Equation (5)). Taking the extraction of ${\mathrm{UO}}_{2}^{2+}$ by SCH‐TODGA system as a representative example. In a neutral extraction system, nonlinear regression fitting yielded great agreement (R^2^ = 0.918), confirming the validity of this decomposition and enabling the explanation of the mechanism (Figure 8a).

(2) $$\Delta \;G_{total}=\Delta G_{elec}+\Delta G_{coord}+\Delta G_{solv}$$

(3) $$\mathrm{lnD}\;=-\frac{\Delta G_{\mathrm{total}}}{\mathrm{RT}}$$

(4) $$\begin{array}{ccc} \Delta \;G_{total} & = & -Az^{2}\frac{{\left[SDS\right]}^{m}}{1+B\sqrt{I}}-RTln\\ & & \times \;\left(C_{1}\cdot {\left[SDS\right]}^{p}\cdot {\left[HFIP\right]}^{q}\cdot {\left[NO_{3}^{-}\right]}^{r}\cdot {\left[L\right]}^{n}\right)+C_{2} \end{array}$$

(5) $$\begin{array}{ccc} lnD & = & a\cdot \frac{{\left[\frac{SDS}{SDS_{med}}\right]}^{m}}{1+b\sqrt{\frac{I}{I_{med}}}}+c+pln\left[SDS\right]+qln\left[HFIP\right]\\ & & +\;rln\left[NO_{3}^{-}\right]+nln\left[L\right] \end{array}$$

where *z* is the metal ion charge, [*SDS*] and [*HFIP*] are the concentrations (M) of SDS and HFIP, respectively, in the condensed phase. *I* is the ionic strength (M), governed by the counter‐ions of the surfactant and HNO_3​_ in this experiment condition. $\left[NO_{3}^{-}\right]$ is the nitrate ion concentration (M), and [*L*] is the extractant concentration (M). *R* is the ideal gas constant (8.314 J·mol^−1^·K^−1^) and *T* is the absolute temperature (*K*). *B* is the Debye‐Hückel constant, dependent on the solvent's dielectric constant and temperature, *b* is the fitting parameter of this item after scaling *I*. In the present fitting, *b* was not treated as an adjustable parameter, because the effects of nitrate concentration and ionic strength are strongly coupled within the explored acidity range, making *b* difficult to identify independently from the experimental data. *A*, *C*_1_​, and *C*_2_​ are constants scaling the strength of interfacial electrostatics, coordination, and solvent effects, respectively. *n* is the apparent extractant stoichiometric coefficient, because the extractant is treated as the direct coordinating ligand in the model. In contrast, *p*, *q*, and *r* are the apparent participation exponents of SDS, HFIP, and HNO_3,_ respectively, describing how these components contribute to the effective free‐energy term through electrostatic association, interfacial organization, solvation effects, and coordination. And $a=\frac{Az^{2}}{RT}\;\;b=B,\;c=lnC=lnC_{1}-\frac{C_{2}}{RT}$ *. a*, *m*, *b*, *c*, *p*, *q*, *r*, *n* are the parameters to be fitted (Table 2).

**TABLE 2:** Nonlinear least‐squares fitted values for the parameters in the equation.

a	*m*	*c*	*p*	*q*	*r*	*n*
0.975	4.0	−7.639	0.014	0.065	2.855	1.183

Analysis of the fitted parameters and the free‐energy decomposition plots reveals a cooperative but condition‐dependent extraction mechanism. The negative Δ*G_elec_* values indicate that the electrostatic attraction between the ${\mathrm{UO}}_{2}^{2+}$, and the negatively charged condensate interface remains a thermodynamically favorable contribution to extraction. In contrast, Δ*G_coord+solv_* is strongly condition‐dependent and changes from positive to negative with increasing extractant and nitrate concentration, indicating that coordination stabilization increasingly outweighs the desolvation penalties of metal ions (Figure 8b). This trend is consistent with the fitted parameters: the exponent *n* = 1.18, and *r* = 2.85 suggest that the higher the concentration of TODGA and HNO_3_, the more negative the free energy within the experimental conditions. By comparison, *p* and *q* are both close to zero, indicating that the explicit concentration dependences of SDS and HFIP in Δ*G_coord_* are weak within the explored range. Overall, these results suggest that electrostatic attraction promotes extraction, while coordination‐solvation reorganization largely determines the thermodynamic feasibility of U(VI) extraction.

Given the limited experimental data, we restrict our discussion to a qualitative assessment. In the SCH system without TODGA, Δ*G_total_* is positive (Figure 8b), indicating that U(VI) extraction is thermodynamically unfavorable and that the extractant is essential for activating extraction. Under conditions of low acidity, Δ*G_coord+solv_* is positive and therefore acts as the principal thermodynamic barrier, even though Δ*G_elec_* is already favorable. With increasing acidity, SDS concentration, and extractant concentration, Δ*G_coord+solv_* decreases and becomes negative. Correspondingly, Δ*G_total_* also becomes negative, indicating that the extraction process is thermodynamically favorable. At the same time, the fractional contribution of Δ*G_coord+solv_* to the overall free‐energy change increases significantly (Figure 8c). The solvent effects term remains constant within the studied range, serving primarily as a baseline offset. Importantly, the fitted apparent extractant stoichiometric coefficient (*n =* 1.18) suggests an approximately 1:1 to 1:2 apparent dependence of extraction on the extractant‐to‐metal concentration ratio, consistent with literature findings [62, 64], but not implying a strict coordination stoichiometry. To assess the robustness of the proposed thermodynamic model against experimental uncertainty, parameter identifiability and sensitivity analyses were performed. While certain fitted parameters exhibit strong mutual correlation, the relative partitioning between electrostatic, coordination, and solvation free‐energy contributions is weakly affected by parameter perturbations, indicating that the mechanistic conclusions are robust. Detailed uncertainty and sensitivity analyses are provided in the Supporting Information (S 6.4–6.6).

Beyond its descriptive function, the model serves as a powerful **design framework for tailoring SCH‐extraction systems** to achieve selective separations. The decomposition analysis provides clear, **qualitative design rules** for tuning solution conditions and extractant properties based on the target ion.

- *High‐valent ions* (e.g., Zr^4+^, Th^4+^): The extraction of high‐valent metal ions focuses on maximizing the electrostatic attraction. Δ*G_elec_* scales with the square of the metal ion charge (*z* ^2^), making it an important contributor to Δ*G_total_* of extraction for ions such as Zr^4+^ and Th^4+^. To optimize this favorable electrostatic contribution, the model provides a clear directive: the interfacial negative charge density ([*SDS*]) must be maximized. Δ*G_elec_* scales with the surfactant concentration through the exponent *m* ([*SDS*]*^m^*). Consequently, increasing the surfactant ratio is the primary lever for amplifying the electrostatic attraction. In addition, the ionic strength (*I*) of the solution is also a critical factor. Δ*G_elec_* is inversely proportional to *I* ($1+B\sqrt{I}$). Therefore, reducing the acidity or the concentration of counterions is a direct method for minimizing ionic strength, thereby reducing the screening effect and further boosting the electrostatic attraction.
- *Divalent ions* (e.g., Sr^2+^, Co^2+^): For divalent ions (*z* = 2), electrostatic contribution is substantially reduced compared to that for tetravalent ions (*z* = 4). This reduction in electrostatic driving force necessitates a design strategy focused on optimizing Δ*G_coord_*. Efficient extraction of divalent ions thus requires extractants with high affinity, such that the resulting favorable Δ*G_coord_* is sufficiently large to overcome the energy penalty from other terms. The coordination term is a function of ligand concentration and its stoichiometric coefficient (*n*ln[*L*]), suggesting that increasing the effective concentration of coordinating ligands also contributes to the extraction of divalent metal ions. The SDS ratio needs to be maximized to minimize the loss of Δ*G_elec_* ​​ as well.
- *Salt‐rich environments*: Δ*G_elec_​*​ is strongly dependent on ionic strength (*I*) through the screening term in the denominator. In salt‐rich environments, the high ionic strength suppresses the electrostatic effect. As a direct consequence of this electrostatic screening, Δ*G_coord_* ​ becomes the controlling factor. This design rule parallels the strategy for extracting divalent ions: efficient extraction requires the use of extractants with high intrinsic affinity.
- *Acidic conditions*: The model was derived with a neutral extractant (TODGA), where the two free energy terms operate relatively independently. Based on our prior spectroscopic analysis, the increase in acidity, together with the nitrate concentration, promotes the formation of more extractable neutral metal‐nitrate‐ligand species. This acidity effect is represented through the [$NO_{3}^{-}$] in Δ*G_coord_*, while ionic strength effects are accounted for separately in the electrostatic term.

This behavior contrasts with that of acidic extractants such as D_2_EHPA, which our current model is not equipped to fully describe. In these systems, increasing acidity not only changes the [$NO_{3}^{-}$], but also leads to proton competition with the metal ion for the extractant, thereby reducing the free ligand concentration, [*L*], and making Δ*G_coord​_* less favorable. Simultaneously, the increased concentration of ${\mathrm{NO}}_{3}^{-}$​ counterions compresses the interfacial double layer and increases the ionic strength, which weakens the electrostatic attraction and makes Δ*G_elec_​* less favorable. The combined effect is a significant increase in Δ*G_total_​*, which dually inhibits extraction. Therefore, to accurately model acidic systems and capture their selectivity trends, Δ*G_coord_* term would need to be modified to reflect the combined influence of both extractant and acidity (e.g., by a term such as ln([*L*]^x^/[H^+^]^y^)). Further research is being conducted on this topic and will be detailed in a forthcoming article.

Taken together, the experimental findings and the fitted model establish a coherent dual‐mechanism picture: electrostatics and coordination act in a tunable synergy, while solvent defines the baseline energy landscape. More importantly, the model provides qualitative design guidance, enabling rational adjustment of extractant identity, surfactant composition, and solution conditions to construct systems optimized for specific separation tasks across diverse metal ions, acidities, and ionic strengths.

### 2.4 Sustainability and Practicality Assessment

**FIGURE 9:**
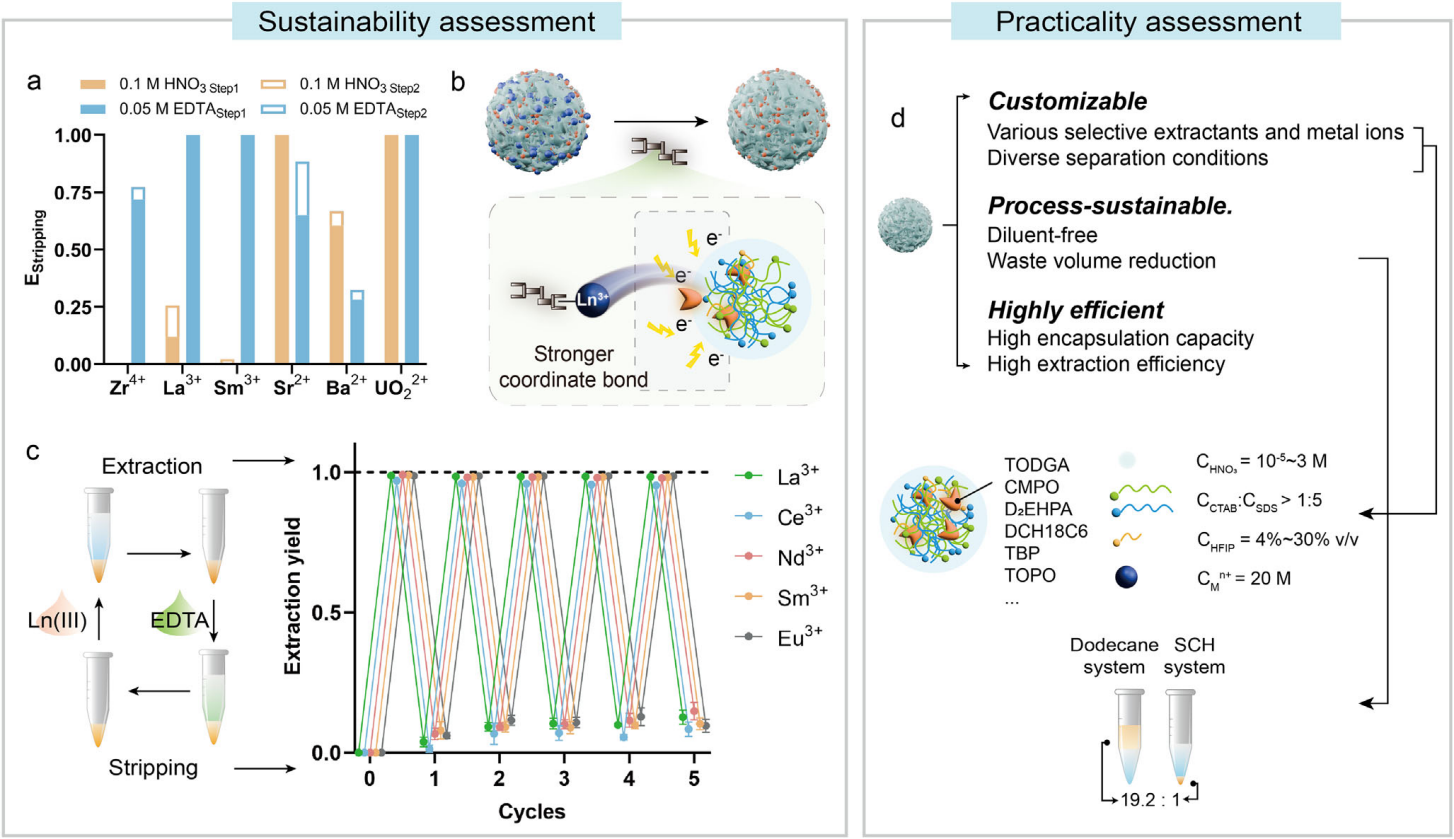
Sustainability and practicality assessment of the SCH‐extraction system. (a) Two‐step stripping ratio of pre‐extracted metal ions by 0.01 M HNO_3_ and strong chelating agent EDTA. Pre‐extraction of metal ions was achieved within SCH‐TODGA system. (b) Schematic of Ln^3+^ stripping by the EDTA. (c) Cycles of extraction and stripping of Ln^3+^ in the SCH‐TODGA system. In each extraction stage, 10 ppm Ln^3+^ is added with the solution acidity adjusted to 1 M HNO_3_. (d) Practicality assessment of SCH‑extraction system [34].

The stripping ratios of the SCH‐TODGA system were evaluated to determine its sustainability. Figure S19 illustrates the concentration of metal ions transferred from the original solution into the condensed phase. Stripping experiments were performed using 0.1 M HNO_3_ and 0.05 M EDTA as individual stripping agents. And the volume of the stripping solution was set to be equal to that of the separated dilute phase. The results reveal distinct stripping performances (Figure 9a). A single‐stage stripping with 0.1 M HNO_3_ achieved nearly 100% recovery of Sr^2+^ and ${\mathrm{UO}}_{2}^{2+}$. However, the two‐stage stripping ratio for La^3+^ and Sm^3+^ was considerably lower, at approximately 25.5% and 2.1%, respectively. In comparison, the strong chelating agent EDTA (0.05 M) achieves nearly 100% stripping ratio for both ${\mathrm{UO}}_{2}^{2+}$ and Ln^3+^. Additionally, the extraction yield of Zr^4+^ after a two‐stage extraction cycle was approximately 77.3%. This indicates that while 0.1 M HNO_3_ offers selective stripping for Sr^2+^ and ${\mathrm{UO}}_{2}^{2+}$, chelating agents like EDTA provide superior performance for comprehensive recovery of metal ions from the SCH‐extraction system (Figure 9b). This versatility in stripping agents allows for tailored recovery strategies depending on the target metal and desired selectivity.

The inherent equilibrium of interactions within the LLPS system ensures their stability in liquid form even after stripping, rendering them well‐suited for multi‐stage serial extraction‐stripping cycles. The reusability of the SCH‐TODGA system over five extraction‐stripping cycles for Ln^3+^ was rigorously tested by employing 0.05 M EDTA for the stripping process. After each Ln^3+^ extraction, the upper dilute phase was separated, and an equivalent volume of 0.05 M EDTA solution was introduced to the remaining condensed phase at each cycle. For subsequent extraction stages, Ln^3+^, HNO_3_, and HFIP were re‐added at their initial concentrations. As shown in Figure 9c, the system maintained a stable extraction yield exceeding 98% throughout five cycles. While a slight decrease in stripping ratio was observed with increasing reuse cycles, potentially due to minor accumulation of metal ions in the residual condensates, the average stripping ratio across the five cycles remained impressively above 92%. These results unequivocally demonstrate the system's impressive recycling performance and sustained efficacy over multiple uses, a critical factor for industrial application. Furthermore, the structural integrity of the SCH‐TODGA system is preserved through multiple extraction‐stripping cycles due to the reversibility of its condensate interface.

The SCH‐extraction system presents advantages over conventional aqueous‐organic biphasic extraction and aqueous two‐phase systems, primarily attributable to its customizability, inherent green chemistry principles, and extraction efficiency. The system's customizability arises from its robust structural stability, which allows for the encapsulation of various hydrophobic extractants (e.g., TODGA, TBP, and DCH18C6) across diverse operating conditions (10^−5^ ∼ 3 M HNO_3_, 4% ∼ 30% v/v [34], $C_{Ln^{3+}}=C_{Zr^{4+}}\;=20\;\mathrm{mM}$, Figure 3). This inherent high stability is a key factor for flexible optimization of reaction conditions, firmly establishing the SCH‐extraction system as a highly adaptable and robust extraction strategy suitable for diverse radioactive ion separation challenges.

From the process‐sustainability perspective, SCH‐extraction the SCH extraction system demonstrates clear feasibility in terms of secondary waste generation, safety, and industrial practicality. A key advantage of the SCH‐extraction system lies in its ability to induce LLPS in the aqueous phase, thereby eliminating the need for traditional hazardous organic diluents such as *n*‐dodecane or kerosene. Unlike traditional solvent extraction processes, which rely on continuous organic phases with a typical 1:1 ratio, the SCH‐extraction system forms concentrated phases with high surfactant, extractant concentrations and limited volumes. For instance, under representative conditions (*C_SDS_* = *C_CTAB_* = 40 mM, *C_TODGA_* = 10 mM, *C_HFIP_* = 4% v/v), the condensed phase volume (*V_con_* ≈ 0.09 mL) is less than 1/19th of the dilute phase volume (*V_dil_* ≈ 1.91 mL). Therefore, the volume of the extraction phase required to treat an equal amount of wastewater containing metal ions is nearly 19.2 times smaller than that used by traditional organic solvent extraction systems. The significant reduction in the volume of the solvent in the extraction phase directly leads to a reduction in radioactive organic secondary waste generation. From a safety perspective, the SCH‐extraction system avoids the use of a large amount of flammable organic diluents. Although SDS, CTAB, and HFIP in this system also have inherent chemical hazards, they are all confined to small‐volume condensed solutions. Compared with large‐volume organic diluent systems, this configuration can reduce the overall risk level and be more manageable. In addition, the components used in the SCH‐extraction system are all commercially available surfactants and cosolvent, and the LLPS only requires simple mixing, stirring and settling, without complex synthesis, high‐salt addition, or external heating. This operational simplicity, together with the reduced solvent volume, contributes to improved industrial feasibility and scalability for potential practical applications.

Finally, SCH‐extraction system demonstrates high efficiency, which is evidenced by high encapsulation capacity for extractants and high extraction yield for target metal ions. In addition, the components forming the condensed phase primarily consist of common commercially available small‐molecule surfactants. This not only keeps economic costs controllable but also ensures that waste disposal benefits from mature commercial treatment methods. These combined attributes firmly establish SCH‐extraction system as a highly practical extraction strategy with immense potential for widespread industrial deployment (Figure 9d), offering a superior alternative for challenging separation processes.

## 3 Conclusions

In this study, we successfully developed a highly customizable, green, and efficient separation strategy using LLPS, termed as the SCH‐extraction system, composed of two oppositely charged small‐molecule surfactants (CTAB and SDS) and HFIP. The SCH‐extraction system facilitates the encapsulation of various commercial hydrophobic extractants (e.g., TODGA, CMPO, D_2_EHPA) via hydrophobic interactions. A key benefit of SCH‐extraction system is organic‐solvent‐free, which allows for a significantly reduced condensed phase volume, leading to a substantial decrease in organic waste generation at the source. Unlike previous non‐ionic compound‐based LLPS‐extraction systems, the SCH‐extraction system requires neither heating nor salt for LLPS induction, thereby eliminating inherent operational complexities and application limitations. Crucially, the molecular design flexibility afforded by its ionic surfactants enables the formation of hydrophobic microdomains with enhanced hydrophobicity and electrostatic tunability. This property improves hydrophobic extractant solubilization, offering solvation capabilities comparable to organic diluents and surpassing those of non‐ionic compound‐based LLPS systems (e.g., C_SDS + CTAB_: C_TODGA_ = 1:6.25). The SCH‐extraction system demonstrated customizable and highly efficient separation of various radioactive metal ions, achieved by appropriately employing extractants and adjusting parameters like surfactant ratio and acidity. Its broad applicability was validated in simulated HLLW, bauxite residues, and uranium mining effluents. Notably, experiments in simulated HLLW showed impressive ${\mathrm{UO}}_{2}^{2+}$ distribution ratios up to 1.31 × 10^3^ and ${\mathrm{UO}}_{2}^{2+}$/Nd^3+^ separation factors reaching 2.33 × 10^4^, outperforming existing extraction methods. This superior efficiency is attributed to a dual extraction mechanism: hydrophobic encapsulation of metal‐extraction complexes within the microdomains, complemented by electrostatic attraction to the negatively charged condensate interface. This fundamental dual mechanism was elucidated by a thermodynamic model, which provides a framework for dissecting the complex extraction process by separating the total Gibbs free energy into distinct electrostatic and coordination components. This approach moves beyond a purely empirical fit, offering a physically‐meaningful basis for understanding how system parameters influence the dominant extraction mechanism, thereby providing guidance for the design of separation schemes for diverse solution conditions and target metal ions. Beyond its performance, the SCH‐extraction system prioritizes practicality. Its components are commercially available, and the system exhibited excellent recyclability during extraction‐stripping cycles. Collectively, these characteristics position the SCH‐extraction system as a promising and adaptable extraction platform with significant potential for further development in process sustainable nuclear waste management and critical metal recovery.

## 4 Experimental Methods

### 4.1 Materials

Sodium dodecyl sulfate (>99%) and Hexadecyl trimethyl ammonium bromide (>98%) were purchased from Sigma‐Aldrich. 1,1,1,3,3,3‐Hexafluoro‐2‐propanol (HFIP) was purchased from Macklin. N,N,N’,N’‐tetraoctyl‐diglycolamide (TODGA, 99.8%), N,N‐Diisobutyl‐2‐(octyl(phenyl)phosphoryl)acetamide (CMPO, 95%), and di‐(2‐ethylhexyl) phosphoric acid (D_2_EHPA, 98%) were purchased from Macklin. was purchased from. All the chemicals were of analytical grade and were used as received without further purification. Water with the resistance of 18.2 M Ω·cm was processed on a Milli‐Q Biocel ultrapure water system.

### 4.2 Preparation of LLPS Solution

Stock solutions of CTAB and SDS were prepared in MilliQ water. Typically, stock solutions were prepared at 100 mM. SCH‐extraction system was prepared by mixing SDS, CTAB and HFIP in a microcentrifuge tube. The mixed solution was shaken at 25°C in a 200 r/min thermostatic shaking water bath for 4 h. Following a sufficient reaction, the solution is centrifuged at a speed of 7900 r/min in a centrifuge for 5 min. The subsequent tests conducted after centrifugation were aged for 1 d.

### 4.3 The Stock Solution Preparation

To simplify the preparation for use of the liquid–liquid separation solution system, the metal ions and surfactants are dissolved in the mother solution prior to usage. SDS and CTAB were first prepared as 200 mM and 100 mM stock solutions in pure water, respectively. All metal ions (Fe^3+^, Cu^2+^, Zr^4+^, Cr^3+^, Ni^2+^, Cu^2+^, Zn^2+^, ${\mathrm{VO}}_{2}^{+}$, La^3+^, Ce^3+^, Nd^3+^, Sm^3+^, Eu^3+^, ${\mathrm{UO}}_{2}^{2+}$, Mg^2+^, Al^3+^, Ca^2+^, Ba^2+^, Sr^2+^) were prepared as 1g/L stock solutions for use. The acidity of the solution is regulated by HNO_3_ and NaOH.

### 4.4 SCH‐Extraction System Characterization

The condensed droplets were observed by an optical microscope (OM) of SCH mixed solutions. The morphology of the fracture surface of condensate is obtained by observing droplets in the condensed phase by cryogenic scanning electron microscopy (cryo‐SEM, Hitachi SU8200) with electron beam energies of 5 kV. UV–vis spectroscopy (UV9000, Metash, Shanghai, China) was operated in scan mode to identify a wavelength without characteristic absorption. Then, the kinetics of phase separation for the SCH‐extraction LLPS mixed solution were monitored in real‐time at this wavelength by tracking the change in absorbance.The rheological properties of the two aqueous phases of different CTAB/SDS ratios and extractant loading were measured using a rheometer (Brookfield DV3T). The apparent ζ‐potentials of the condensates formed in the SCH‐extraction LLPS system with different surfactant ratio were measured using a Malvern Panalytical Zetasizer Ultra. Prior to the measurements, the samples were sonicated for 5 min at 25°C. A folded capillary cell (DTS1070) was employed, and a pre‐equilibration time of 120 s was maintained for each run.

### 4.5 Extractant‐Loading Capacity Characterization

The extractant‐loading capacity of the SCH‐extraction system was evaluated by quantifying residual extractant content in the dilute phase. SDS, CTAB, and CMPO powder were added directly into the centrifuge tube, while the liquid phase of HFIP, TODGA, and D_2_EHPA in analytical grade were directly added. SCH‐TODGA/CMPO/D_2_EHPA stock solutions were prepared in D_2_O, DNO_3_ media. Following phase separation, aliquots of the dilute phase were transferred to NMR tubes for ^1^H NMR analysis using Bruker Ascend 400 MHz spectrometer (Germany). MestRenova 11.0 software was used to process the correct baselines and predict the ^1^H NMR spectra. Fourier Transform Infrared Spectroscopy (FT‐IR) was acquired at room temperature in the attenuated total reflection (ATR) mode on 400 MHz Bruker Alpha II by detecting changes in peak positions and peak intensities from 500 to 4000 cm^−1^ wavenumbers. To eliminate interference from water absorption bands, LLPS samples were prepared in D_2_O with an extractant‐to‐metal ion molar ratio of 4:1. The substrate water vapors, correct baselines, normalized signal intensities, and deconvolution were processed by OPUS 6.5 software. Surfactant distribution between phases was determined through Total Organic Carbon (TOC) analysis of the dilute phase using a Jena Multi N/C 3100 analyzer operated in TC‐IC (Total Carbon‐Inorganic Carbon) mode, following established indirect measurement protocols.

### 4.6 Thermodynamic Experiment of Metal Ion Extraction

The separation efficiency and selectivity of the SCH‐extraction system for metal ions were evaluated by measuring their concentrations in the dilute liquid phase. Metal ions were added using stock solutions, and the order of reagent addition did not impact the extraction efficiency. To maintain consistency and control influencing factors, the reagents were added in the following sequence: CTAB, SDS, HFIP, metal ions, extractant, and acid. The LLPS solution was prepared as detailed in the section on *preparation of LLPS solution*. Metal ion concentrations were determined using an inductively coupled plasma optical emission spectrometer (ICP‐OES, Agilent 5110). Extraction yield (E) was calculated by comparing the metal ion concentrations before and after extraction, using the formula:

(6) $$E=\frac{c_{2}V_{2}}{c_{0}V_{0}}=1-\frac{c_{1}V_{1}}{c_{0}V_{0}}$$

where *c_0_* (mg/L) represents the initial metal concentration in the mixed solution before phase separation, while *c_1_* (mg/L) and *c_2_* (mg/L) represent the metal ion concentrations after extraction in the dilute and the condensed phase, respectively. *V_0_* (mL) represents the total initial volume of the mixed solution, while *V_1_* (mL) and *V_2_* (mL) are the volumes of the dilute and the condensed phase after phase separation, respectively (*V_0_* ≈ *V_1+_ V_2_*). The volume of the condensed phase is measured using a pipette. All the error bars indicate standard deviation, unless otherwise noted.

The difference between these two concentrations corresponds to the concentration of metal ions initially added that were extracted into the condensed phase. To express the extraction of the system for metal ions, the distribution ratio (*D*) is used, defined as:

(7) $$D=\frac{c_{2}}{c_{1}}=\frac{c_{0}V_{0}-c_{1}V_{1}}{c_{1}V_{2}}$$

### 4.7 MD Simulation

Molecular Dynamics (MD) simulation of these three solution systems was carried out using the Gromacs program suite [65], with the hybridized force field of CL&P forcefield [66], Merz force field [67, 68] and OPLS‐AA force field [69]. The CL&P force field was used to describe SDS anion, CTAB cation, bromide, sodium, and nitrite anion. The neutral organic molecules of HFIP and DGA were all parameterized using OPLS‐AA force field [69]. The zirconium and lanthanum ions were simulated using Merz forcefield and the water molecules were simulated using OPC3 water model [70]. All these topology files of these molecules and ions were generated directly using the all‐atom and ionic‐liquid modules of AuToFF web server [71].

The initial simulation boxes contained the solution components with dimensions of 10 × 10 × 10 nm^3^, were created using the packmol program [72]. The structures were first energy‐minimized and then annealed from 0 to 400 K and then cooled down over a 1 ns time period with a time step of 1 ps, to reach an equilibrium state. The temperature was maintained at 298.15 K using the velocity‐rescale thermostat [73] with a relaxation constant of 1 ps. The pressure was maintained at 1.01325 × 10^5^ Pa using Berendsen's barostat with an isothermal compressibility constant of 4.5 × 10^−5^. Periodic boundary conditions were applied in all directions, and the electrostatic interactions and van der Waals forces were treated using the Particle‐mesh Ewald (PME) method with a cut‐off distance of 15 Å.

Following the energy‐minimization and equilibration steps, a 20 ns MD simulation at NVT ensemble was performed, with the trajectory saved every 1 ps. The further statistics results were analyzed from the trajectory data by Gromacs tool‐suites. The simulation box was rendered using Visual Molecular Dynamic program (VMD) [74].

### 4.8 System Regeneration and Reuse

The cyclic operation of the SCH‐extraction system primarily involved metal ion extraction, stripping, and solvent recovery. Following metal ion extraction as described above, the condensed phase was contacted with 0.05 M EDTA/0.1 M HNO_3_ solution and vigorously agitated for 4 h at 25°C in a constant temperature shaking water bath at 200 rpm. For ease of stripping ratio calculations, the volume of the added stripping solution matched that of the separated dilute phase. Subsequently, the mixture was centrifuged and allowed to settle for 24 h (a 24‐h settling period was maintained between each subsequent extraction/stripping cycle). This completed one extraction‐stripping cycle. The upper stripped phase was then removed, and the remaining metal‐ions‐unloaded condensed phase was recharged with an equal concentration of metal ions and HFIP. The solution acidity was adjusted to 1 M HNO_3_ to initiate the next extraction cycle. The stripping rate *E_s_* is calculated using the following formula:

(8) $$E_{s}=\frac{c_{1}V_{1}}{c_{0}V_{0}}$$

## Author Contributions

R.Y. and W.W. designed the experiments. R.Y. and Z.C. conducted the LLPS system phase behavior experiments. R.Y. and Y.H. performed the metal ion extraction and characterization experiments. W.W. and M.C. provided information related to MD simulations. G.Y., T.Y., W.W., and M.C. provided suggestions and technical support. G.Y. and T.Y. supervised the study. R.Y., Y.H., W.W., Z.C., M.C., G.Y., and T.Y. analyzed the data. R.Y. wrote the initial manuscript with assistance and input from all authors. All authors approved the final version before submission.

## Funding

This work was supported by the Continuous‐Support Basic Scientific Research Project (BJ030261224901, BJ030261224862).

## Conflicts of Interest

The authors declare no conflicts of interest.

## Supporting information




**Supporting File 1**: advs75493‐sup‐0001‐SuppMat.docx.

**Supporting File 2**: advs75493‐sup‐0002‐VideoS1.mp4.

## Data Availability

The main data supporting the findings of this study are available from the corresponding author upon reasonable request.

## References

[1] S. Park and R. C. Ewing , “US Legal and Regulatory Framework for Nuclear Waste from Present and Future Reactors and Their Fuel Cycles,” Annual Review of Environment and Resources 48 (2023): 713–736.

[2] J. Li , X. Wang , G. Zhao , et al., “Metal–Organic Framework‐Based Materials: Superior Adsorbents for the Capture of Toxic and Radioactive Metal Ions,” Chemical Society Reviews 47 (2018): 2322–2356. 29498381 10.1039/c7cs00543a

[3] J. Veliscek‐Carolan , “Separation of Actinides from Spent Nuclear Fuel: a Review,” Journal of Hazardous Materials 318 (2016): 266–281. 27427893 10.1016/j.jhazmat.2016.07.027

[4] M. Espino , M. De Los Ángeles Fernández , F. J. V. Gomez , and M. F. Silva , “Natural Designer Solvents for Greening Analytical Chemistry,” Trends in Analytical Chemistry 76 (2016): 126–136.

[5] S. F. Banani , H. O. Lee , A. A. Hyman , and M. K. Rosen , “Biomolecular Condensates: Organizers of Cellular Biochemistry,” Nature Reviews Molecular Cell Biology 18 (2017): 285–298. 28225081 10.1038/nrm.2017.7 PMC7434221

[6] N. A. McDonald , R. D. Fetter , and K. Shen , “Assembly of Synaptic Active Zones Requires Phase Separation of Scaffold Molecules,” Nature 588 (2020): 454–458. 33208945 10.1038/s41586-020-2942-0

[7] S. Cao , P. Zhou , G. Shen , et al., “Binary Peptide Coacervates as an Active Model for Biomolecular Condensates,” Nature Communications 16 (2025): 2407. 10.1038/s41467-025-57772-z PMC11897134 40069227

[8] P. Mukherjee , S. K. Padhan , S. Dash , S. Patel , and B. K. Mishra , “Clouding Behaviour in Surfactant Systems,” Advances in Colloid and Interface Science 162 (2011): 59–79. 21296314 10.1016/j.cis.2010.12.005

[9] Y. Cheng , E. Hirano , H. Wang , et al., “Mechanically Strong yet Metabolizable Supramolecular Plastics by Desalting Upon Phase Separation,” Science 386 (2024): 875–881. 39571014 10.1126/science.ado1782

[10] T. I. Zvarova , V. M. Shkinev , G. A. Vorob'eva , B. Ya Spivakov , and Y. A. Zolotov , “Liquid‐Liquid Extraction in the Absence of Usual Organic Solvents: Application of Two‐Phase Aqueous Systems Based on a Water‐Soluble Polymer,” Mikrochimica Acta 84 (1984): 449–458.

[11] A. M. Rumyantsev , N. E. Jackson , and J. J. De Pablo , “Polyelectrolyte Complex Coacervates: Recent Developments and New Frontiers,” Annual Review of Condensed Matter Physics 12 (2021): 155–176.

[12] G. Zhu , J. Yu , R. Zhang , et al., “A Natural Deep Eutectic Solvent‐Based Aqueous Biphasic System Coupled with MoS_2_ Photocatalytic Reduction for Green Recovery of Gold from Thiosulfate Solution,” Green Chemistry 24 (2022): 8330–8344.

[13] A. U. Oya , Y. G. Zeynep , D. Sabahattin , K. Y. Ece , and A. Adnan , “A Novel Ligand for Cloud Point Extraction to Determine Gold Content in Ore Samples,” Environmental Chemistry Letters 12 (2014): 449–453.

[14] J. Chen , W. Shi , Y. Ren , et al., “Strong Protein Adhesives through Lanthanide‐Enhanced Structure Folding and Stack Density,” Angewandte Chemie International Edition 62 (2023): 202304483. 10.1002/anie.202304483 37670725

[15] R. Karmakar and K. Sen , “Aqueous Biphasic Extraction of Metal Ions: an Alternative Technology for Metal Regeneration,” Journal of Molecular Liquids 273 (2019): 231–247.

[16] H. Fu , J. Huang , J. J. B. Van Der Tol , et al., “Supramolecular Polymers Form Tactoids through Liquid–Liquid Phase Separation,” Nature 626 (2024): 1011–1018. 38418913 10.1038/s41586-024-07034-7 PMC10901743

[17] H. Passos , S. H. Costa , A. M. Fernandes , M. G. Freire , R. D. Rogers , and J. A. P. Coutinho , “A Triple Salting‐out Effect Is Required for the Formation of Ionic‐Liquid‐Based Aqueous Multiphase Systems,” Angewandte Chemie International Edition 56 (2017): 15058–15062. 28967998 10.1002/anie.201705704 PMC6157712

[18] H. P. Neves , L. A. Silva , L. M. Prates , et al., “Aqueous Two‐Phase Systems for Environmentally Friendlier Separation of Rare Earth Elements and Transition Metals: Applications and New Molecular Insights,” Chemical Engineering Journal 502 (2024): 158083.

[19] M. Le , W. Huang , K.‐F. Chen , et al., “Upper Critical Solution Temperature Polymeric Drug Carriers,” Chemical Engineering Journal 432 (2022): 134354.

[20] Z. Zhang , H. Li , S. Kasmi , et al., “A Synthetic, Transiently Thermoresponsive Homopolymer with UCST Behaviour within a Physiologically Relevant Window,” Angewandte Chemie International Edition 58 (2019): 7866–7872. 30925024 10.1002/anie.201900224

[21] R. D. Rogers , A. H. Bond , J. Zhang , and E. P. Horwitz , “New Technetium‐99m Generator Technologies Utilizing Polyethylene Glycol‐Based Aqueous Biphasic Systems,” Separation and Purification Technology 32 (1997): 867–882.

[22] L. R. De Lemos , I. J. B. Santos , G. D. Rodrigues , L. H. M. Da Silva , and M. C. H. Da Silva , “Copper Recovery from Ore by Liquid–Liquid Extraction Using Aqueous Two‐Phase System,” Journal of Hazardous Materials 237‐238 (2012): 209–214. 10.1016/j.jhazmat.2012.08.028 22959476

[23] Y. Chao and H. C. Shum , “Emerging Aqueous Two‐Phase Systems: from Fundamentals of Interfaces to Biomedical Applications,” Chemical Society Reviews 49 (2020): 114–142. 31750468 10.1039/c9cs00466a

[24] A. P. Paiva and P. Malik , “Recent Advances on the Chemistry of Solvent Extraction Applied to the Reprocessing of Spent Nuclear Fuels and Radioactive Wastes,” Journal of Radioanalytical and Nuclear Chemistry 261 (2004): 485–496.

[25] E. J. Creatto , F. B. Okasaki , M. B. Cardoso , and E. Sabadini , “Wormlike Micelles of CTAB with Phenols and with the Corresponding Phenolate Derivatives‐When Hydrophobicity and Charge Drive the Coacervation,” Journal of Colloid and Interface Science 627 (2022): 355–366. 35863194 10.1016/j.jcis.2022.07.044

[26] J. M. Katona , V. J. Sovilj , and L. B. Petrović , “Microencapsulation of Oil by Polymer Mixture–Ionic Surfactant Interaction Induced Coacervation,” Carbohydrate Polymers 79 (2010): 563–570.

[27] C. Paganini , U. Capasso Palmiero , S. Picciotto , et al., “High‐Yield Separation of Extracellular Vesicles Using Programmable Zwitterionic Coacervates,” Small 19 (2023): 2204736. 10.1002/smll.202204736 36367966

[28] A. S. Yazdi , “Surfactant‐Based Extraction Methods,” TrAC Trends in Analytical Chemistry 30 (2011): 918–929.

[29] P. Zhang , Y. Zhang , F. Wu , et al., “Photoisomerization‐Mediated Tunable Pore Size in Metal Organic Frameworks for U(VI)/V(V) Selective Separation,” Nature Communications 16 (2025): 2361. 10.1038/s41467-025-57638-4 PMC11894057 40064899

[30] K. Liu , Y.‐L. Liu , Z.‐F. Chai , and W.‐Q. Shi , “Electroseparation of Uranium from Lanthanides (La, Ce, Pr, Nd and Sm) on Liquid Gallium Electrode (La, Ce, Pr, Nd and Sm) on Liquid Gallium Electrode,” Separation and Purification Technology 265 (2021): 118524.

[31] Y. Huang , D. Chen , S. Chen , et al., “A Green Method for Recovery of Thallium and Uranium from Wastewater Using Polyethylene Glycol and Ammonium Sulfate Based on Aqueous Two‐Phase System,” The Journal of Cleaner Production 297 (2021): 126452.

[32] A. Molinaro , C. De Castro , R. Lanzetta , E. Manzo , and M. Parrilli , “Solvent Effect on the Isomeric Equilibrium of Carbohydrates: the Superior Ability of 2,2,2‐Trifluoroethanol for Intramolecular Hydrogen Bond Stabilization,” Journal of the American Chemical Society 123 (2001): 12605–12610. 11741425 10.1021/ja016471i

[33] B. Gabryelczyk , H. Cai , X. Shi , et al., “Hydrogen Bond Guidance and Aromatic Stacking Drive Liquid‐Liquid Phase Separation of Intrinsically Disordered Histidine‐Rich Peptides,” Nature Communications 10 (2019): 5465. 10.1038/s41467-019-13469-8 PMC6884462 31784535

[34] S. I. Jenkins , C. M. Collins , and M. G. Khaledi , “Perfluorinated Alcohols Induce Complex Coacervation in Mixed Surfactants,” Langmuir 32 (2016): 2321–2330. 26881998 10.1021/acs.langmuir.5b04701

[35] L. Zhou , Z. Liu , and Y. Wang , “Molecular Insights: How Counterions Determine Surfactant Aggregation,” Advances in Colloid and Interface Science 341 (2025): 103484. 40157336 10.1016/j.cis.2025.103484

[36] D. J. Tobias and J. C. Hemminger , “Getting Specific about Specific Ion Effects,” Science 319 (2008): 1197–1198. 18309069 10.1126/science.1152799

[37] C. Yuan , A. Levin , W. Chen , et al., “Nucleation and Growth of Amino Acid and Peptide Supramolecular Polymers through Liquid–Liquid Phase Separation,” Angewandte Chemie International Edition 58 (2019): 18116–18123. 31617663 10.1002/anie.201911782

[38] Q. Zhao , L. Wen , M. Moniruzzaman , F. Wu , P. Gao , and K. Shih , “Cloud Point Extraction of Sr(II) from Simulated High‐Level Liquid Waste: Minimizing Radioactive Organic Liquid Volume,” Chemical Engineering Science 312 (2025): 121673.

[39] H. Zhang , L. Deng , B. Zeeb , and J. Weiss , “Solubilization of Octane in Cationic Surfactant–Anionic Polymer Complexes: Effect of Polymer Concentration and Temperature,” Journal of Colloid and Interface Science 450 (2015): 332–338. 25841059 10.1016/j.jcis.2015.03.003

[40] N. Watanabe , S. Watase , N. Kadonishi , Y. Okamoto , and H. Umakoshi , “Revealed Properties of Various Self‐Assemblies in Two Catanionic Surfactant Systems in Relation to Their Polarity and Molecular Packing State,” Langmuir 38 (2022): 14768–14778. 36437713 10.1021/acs.langmuir.2c02411

[41] L. Zhou , Y. Fan , Z. Liu , L. Chen , E. Spruijt , and Y. Wang , “A Multiresponsive Transformation between Surfactant‐Based Coacervates and Vesicles,” CCS Chemistry 3 (2021): 358–366.

[42] C. S. G. Butler , V. T. Kelleppan‐Meaney , A. P. Williams , et al., “Influence of Tail Group Length, Amide Functionality and Added Salt Ion Identity on the Behaviour of Betaine Surfactants,” Journal of Colloid and Interface Science 653 (2024): 338–350. 37717434 10.1016/j.jcis.2023.08.171

[43] A. Melnyk , J. Namieśnik , and L. Wolska , “Theory and Recent Applications of Coacervate‐Based Extraction Techniques,” Trends in Analytical Chemistry 71 (2015): 282–292.

[44] M. Novakovic , Y. Han , N. C. Kathe , Y. Ni , L. Emmanouilidis , and F. H.‐T. Allain , “LLPS REDIFINE Allows the Biophysical Characterization of Multicomponent Condensates without Tags or Labels,” Nature Communications 16 (2025): 4628. 10.1038/s41467-025-59759-2 PMC12089286 40389460

[45] L. Li , S. Srivastava , M. Andreev , A. B. Marciel , J. J. De Pablo , and M. V. Tirrell , “Phase Behavior and Salt Partitioning in Polyelectrolyte Complex Coacervates,” Macromolecules 51 (2018): 2988–2995.

[46] Z.‐X. Zhu , Y. Sasaki , H. Suzuki , S. Suzuki , and T. Kimura , “Cumulative Study on Solvent Extraction of Elements by N,N,N′,N′‐Tetraoctyl‐3‐Oxapentanediamide (TODGA) from Nitric Acid into n‐Dodecane,” Analytica Chimica Acta 527 (2004): 163–168.

[47] R. K. Biswas and D. A. Begum , “Solvent Extraction of Fe^3+^ from Chloride Solution by D_2_EHPA in Kerosene,” Hydrometallurgy 50 (1998): 153–168.

[48] K. Binnemans , P. T. Jones , B. Blanpain , T. Van Gerven , and Y. Pontikes , “Towards Zero‐Waste Valorisation of Rare‐Earth‐Containing Industrial Process Residues: a Critical Review,” Journal of Cleaner Production 99 (2015): 17–38.

[49] L. Sheng , D. Ding , and H. Zhang , “Efficient Removal of Uranium from Acidic Mining Wastewater Using Magnetic Phosphate Composites,” Separation and Purification Technology 337 (2024): 126397.

[50] Y.‐H. Wang , Y.‐F. Wang , Y.‐T. Li , et al., “A Review on Vanadium Extraction Techniques from Major Vanadium‐Containing Resources,” Rare Metals 43 (2024): 4115–4131.

[51] H. F. Motiwala , A. M. Armaly , J. G. Cacioppo , et al., “HFIP in Organic Synthesis,” Chemical Reviews 122 (2022): 12544–12747. 35848353 10.1021/acs.chemrev.1c00749

[52] J. V. Gavette , I. D. Petsalakis , G. Theodorakopoulos , K.‐D. Zhang , Y. Yu , and J. Rebek , “The Effects of Hexafluoroisopropanol on Guest Binding by Water‐Soluble Capsule and Cavitand Hosts,” Chemical Communications 51 (2015): 17604–17606. 26482864 10.1039/c5cc06405h

[53] S. Bag , S. K , A. Mondal , et al., “Palladium‐Catalyzed Meta ‐C–H Allylation of Arenes: a Unique Combination of a Pyrimidine‐Based Template and Hexafluoroisopropanol,” Journal of the American Chemical Society 142 (2020): 12453–12466. 32496791 10.1021/jacs.0c05223

[54] Y. Chen , S. Ning , Y. Zhong , et al., “Study on Highly Efficient Separation of Zirconium from Scandium with TODGA‐Modified Macroporous Silica‐Polymer Based Resin,” Separation and Purification Technology 305 (2023): 122499.

[55] J. Huang , Y. Jiang , X. Du , and J. Bi , “Sens,” Sensors and Actuators B: Chemical 146 (2010): 388–394.

[56] Y. Zhao , N. Khalid , G. Shu , M. A. Neves , I. Kobayashi , and M. Nakajima , “Complex Coacervates from Gelatin and Octenyl Succinic Anhydride Modified Kudzu Starch: Insights of Formulation and Characterization,” Food Hydrocolloids 86 (2019): 70–77.

[57] C. S. Kesava Raju and M. S. Subramanian , “Sequential Separation of Lanthanides, Thorium and Uranium Using Novel Solid Phase Extraction Method from High Acidic Nuclear Wastes,” Journal of Hazardous Materials 145 (2007): 315–322. 17178189 10.1016/j.jhazmat.2006.11.024

[58] Y. Liu and M. S. Lee , “Analysis of the Interaction between Organophosphorus Acid and Tertiary Amine Extractants in the Binary Mixtures by Fourier Transform Infrared Spectroscopy (FT‐IR),” Solvent Extraction and Ion Exchange 34 (2016): 74–85.

[59] J. N. Israelachvili , Intermolecular and Surface Forces, 3rd Edition (Academic Press, 2011), 163.

[60] T. Lu and E. Spruijt , “Multiphase Complex Coacervate Droplets,” Journal of the American Chemical Society 142 (2020): 2905–2914. 31958956 10.1021/jacs.9b11468 PMC7020193

[61] Q. Li , X. Wang , L. Song , et al., “Cyclohexyl Substituted Diglycolamide Ligands for Highly Efficient Separation of Strontium: Synthesis, Extraction and Crystallography Studies,” Journal of Environmental Chemical Engineering 11 (2023): 110495.

[62] A. N. Turanov , V. K. Karandashev , M. Boltoeva , C. Gaillard , and V. Mazan , “Synergistic Extraction of Uranium(VI) with TODGA and Hydrophobic Ionic Liquid Mixtures into Molecular Diluent,” Separation and Purification Technology 164 (2016): 97–106.

[63] D. Bashford and D. A. Case , “Generalized Born Models of Macromolecular Solvation Effects,” Annual Review of Physical Chemistry 51 (2000): 129–152. 10.1146/annurev.physchem.51.1.129 11031278

[64] S. Panja , P. K. Mohapatra , P. Kandwal , and S. C. Tripathi , “Uranium(VI) Pertraction across a Supported Liquid Membrane Containing a Branched Diglycolamide Carrier Extractant: Part III,” Desalination 285 (2012): 213–218.

[65] M. J. Abraham , T. Murtola , R. Schulz , et al., “GROMACS: High Performance Molecular Simulations through Multi‐Level Parallelism from Laptops to Supercomputers,” SoftwareX 1–2 (2015): 19–25.

[66] J. N. Canongia Lopes and A. A. H. Pádua , “CL&P: a Generic and Systematic Force Field for Ionic Liquids Modeling,” Theoretical Chemistry Accounts 131 (2012): 1129.

[67] Z. Li , L. F. Song , P. Li , and K. M. Merz , “Systematic Parametrization of Divalent Metal Ions for the OPC3, OPC, TIP3P‐FB, and TIP4P‐FB Water Models,” Journal of Chemical Theory and Computation 16 (2020): 4429–4442. 32510956 10.1021/acs.jctc.0c00194 PMC8173364

[68] P. Li , B. P. Roberts , D. K. Chakravorty , and K. M. Merz , “Rational Design of Particle Mesh Ewald Compatible Lennard‐Jones Parameters for +2 Metal Cations in Explicit Solvent,” Journal of Chemical Theory and Computation 9 (2013): 2733–2748. 23914143 10.1021/ct400146w PMC3728907

[69] M. J. Robertson , Y. Qian , M. C. Robinson , J. Tirado‐Rives , and W. L. Jorgensen , “Development and Testing of the OPLS‐AA/M Force Field for RNA,” Journal of Chemical Theory and Computation 15 (2019): 2734–2742. 30807148 10.1021/acs.jctc.9b00054 PMC6585454

[70] S. Izadi and A. V. Onufriev , “Accuracy Limit of Rigid 3‐Point Water Models,” The Journal of Chemical Physics 145 (2016): 074501. 27544113 10.1063/1.4960175 PMC4991989

[71] C. Wang , W. Li , K. Liao , Z. Wang , Y. Wang , and K. Gong , “AuToFF Program, Version 1.0. Hzwtech, Shanghai,” (2023), https://cloud.hzwtech.com/web/product-service?id=36.

[72] L. Martínez , R. Andrade , E. G. Birgin , and J. M. Martínez , “PACKMOL: a Package for Building Initial Configurations for Molecular Dynamics Simulations,” Journal of Computational Chemistry 30 (2009): 2157–2164. 19229944 10.1002/jcc.21224

[73] G. Bussi , D. Donadio , and M. Parrinello , “Canonical Sampling through Velocity Rescaling,” The Journal of Chemical Physics 126 (2007): 014101. 17212484 10.1063/1.2408420

[74] W. Humphrey , A. Dalke , and K. Schulten , “VMD: Visual Molecular Dynamics,” Journal of Molecular Graphics 14 (1996): 33–38. 8744570 10.1016/0263-7855(96)00018-5

