# Supplementary material for: A New Customizable Surfactant LLPS Strategy for Sustainable and Highly Efficient Radioactive Metal Ion Separation
Source: Adv Sci (Weinh). 2026 May 18;13(41):e75493. doi: 10.1002/advs.75493 (PMC13335465; doi:10.1002/advs.75493)
Supplement: Supplementary file 1 — Supporting File 1: advs75493‐sup‐0001‐SuppMat.docx. [file ADVS-13-e75493-s002.docx]

**Supporting Information**

**A New Customizable Surfactant LLPS Strategy for Sustainable and Highly Efficient Radioactive Metal Ion Separation**

Ruihan Yan, Yifu Hu, Wentao Wang*, Zhi Cao, Meiwen Cao, Weifang Zheng, Guoan Ye*, Taihong Yan*

Ruihan Yan, Yifu Hu, Wentao Wang, Zhi Cao, Weifang Zheng, Guoan Ye, Taihong Yan

Department of Radiochemistry, China Institute of Atomic Energy, Beijing 102413, China
E-mail: wangwt@ciae.ac.cn; caozhi@cnncmail.cn; yeguoan@cnncmail.cn; yantaihong@cnncmail.cn

Meiwen Cao
State Key Laboratory of Heavy Oil Processing & Department of Biological and Energy Chemical Engineering, College of Chemical Engineering, China University of Petroleum (East China), 66 Changjiang West Road, Qingdao 266580, China

**Table of Contents**

**Supplementary discussion3**

**Tables S1-310**

**Figures S1-S2012**

**Reference23**

**Supplementary Discussion**

**S1. Kinetic analysis of LLPS formation**

To characterize the kinetics of the phase separation process, the gravitational sedimentation of condensed droplets was monitored via UV-vis spectrophotometry under varying conditions (SDS/CTAB ratios, HFIP concentrations, and acidities). Measurements were performed at 500 nm, a wavelength selected due to the absence of electronic transitions for all components in the dilute phase within the 400~700 nm range, ensuring that the absorbance change reflects the change of turbidity. A kinetic parameter, *t*_9/10_ (defined as the time required for a 90% reduction in initial absorbance), was employed to quantify the separation rate.

The results indicate that increasing the SDS fraction from 0 to 40 mM (within a total surfactant concentration of 80 mM) significantly accelerated the separation, with *t*_9/10_ decreasing from 60 to 20 min (**Figure S3a**). Similarly, increasing the HFIP concentration from 4% to 12% v/v enhanced the sedimentation velocity, thereby reducing *t*_9/10_ by 12 mins (**Figure S3b**). In contrast, the separation kinetics remained relatively insensitive to acidity variations across a wide range (10^-5^ to 3 M HNO_3_) (**Figure S3c**).

**S2. Viscosity of the condensed phase**

Viscosity significantly influences the two-phase contacting and the mass transfer efficiency, as long as the stress and droplet size are fixed. Shear flow experiments of SCH-extraction system are carried out at 25 ℃. As depicted in **Figure S2a** and **Figure S2b**, SCH-extraction system exhibited different rheological properties with different extractants. The SCH system without extractant exhibited pseudoplastic shear-thinning behavior with significantly lower apparent viscosity than the extractant-loaded systems (**Figure S2a**). The shear-thinning property of the condensate may be attributed to the structural breakdown or rearrangement under shear.^[1]^ In contrast, the SCH-extractant systems displayed Bingham plastic characteristics,^[2]^ evidenced by a distinct yield stress followed by abrupt viscosity reduction under shear. Notably, while extractant hydrophobicity variations caused minimal viscosity differences among SCH-extractant systems, the condensates consistently demonstrated higher viscosities than the SCH system without extractant. Extractant addition strengthened intermolecular interactions, creating a densely packed microstructure that resists deformation until reaching a critical shear threshold.^[3]^ In **Figure S2b**, ​the viscosity of the condensate with varying SDS/CTAB ratios was measured. Replacing SDS with CTAB (55:25→0:80) enhanced pseudoplasticity and triggered a transition to Bingham plastic behavior. All TODGA-loaded systems exhibited Bingham fluid behavior, with viscosities remaining stable (<18 mPa·s) regardless of the SDS/CTAB ratio. This invariability suggests that TODGA dominates the intermolecular interactions, thereby stabilizing condensate microstructure against surfactant ratio variations.

**S3. Encapsulation of extractant**

The encapsulation behavior of various extractants within the SCH-extraction LLPS system was investigated, particularly focusing on the subtle influences of different extractants and phase-forming agents on the system's internal structure. Upon adding 80 mM of TODGA, CMPO, or D_2_EHPA (C_SDS_ : C_CTAB_ = 1:1), the HFIP ^1^H NMR chemical shifts remained within a narrow range of 4.43~4.44 ppm. This minor shift can be attributed to the varying polarities of the added extractants, which subtly influence the hydration and solvent effects around HFIP. For instance, the higher electronegativity of the -C=O group in CMPO compared to the -P=O group in D_2_EHPA can lead to a decrease in the electron cloud density of adjacent hydrogen nuclei upon HFIP coordination, resulting in a slight upfield shift.^[4]-[5]^ Despite these minor alterations, the overall hydrogen bonding network and solvation shell of HFIP in the dilute phase remained largely unaffected. This suggests that the predominant HFIP-H_2_O or HFIP-HFIP interactions continue to govern the HFIP proton environment in this phase. Similarly, maintaining an 80 mM total concentration of SDS+CTAB while varying the SDS:CTAB ratio (e.g., 0:80, 40:40, 55:25) with 80 mM TODGA showed that the main peak chemical shift remained consistently within 4.43~4.44 ppm. These findings indicate that the LLPS internal structure is robust against minor perturbations from different extractants or surfactant ratios at these concentrations.

However, a more pronounced structural response was observed at higher concentrations. When the concentrations of CTAB and SDS were doubled, and the concentration of HFIP was tripled, the chemical shift of the main peak of the HFIP septet exhibited a statistically significant upfield shift (from 4.44/4.43 ppm to 4.40 ppm). This notable shift is likely due to the enhanced electrostatic interactions between cations and anions within the system at increased surfactant concentrations. This is attributed to strengthened hydrogen bonding between HFIP and the surfactants, which reduces the electron cloud density at the HFIP H1 position.^[6]-[7]^ This indicates that while the system is robust to minor changes, significant increases in component concentrations can induce more substantial alterations in its internal structure, primarily driven by amplified intermolecular forces.

**S4. High Efficiency of SCH-extraction system**

The SCH-extraction system achieved high single-stage extraction yields across various conditions. As shown in **Figure S6a-d**, a comparative evaluation involving four distinct extractants (TODGA, CMPO, D_2_EHPA, and DCH18C6) within the SCH-extraction system was conducted to assess their extraction yields and distribution ratios for Ln^3+^, UO_2_^2+^, Fe^3+^, and Sr^2+^.

In the SCH-TODGA system, Ln^3+^ (La^3+^, Ce^3+^, Nd^3+^, and Eu^3+^) yields reached ~100%, with distribution ratios (*D* = 10^3^ ~ 10^5^) three orders of magnitude higher than conventional *n*-dodecane systems (10^0^ ~ 10^2^, **Figure S6a**).^[8]^

In the SCH-CMPO system, the extraction yield of UO_2_^2+^ exhibited pH-dependent fluctuations, ranging from 68% to 80% (**Figure S6b**). This behavior contrasts with the monotonic increase in *D* typically observed in *n*-dodecane/isodecanol (10% v/v) systems.^[9]^ Notably, while the extraction yield of UO_2_^2+^ when present as a single ion in solution ranged from 68% to 80%, it sharply contrasted with the nearly 100% extraction yield observed in a mixed ion solution (**Figure S5**). This deviation suggests synergistic effects from Ln^3+^ and Zr^4+^, likely mediated by: (1) direct electrostatic attraction between high-valence cations and SDS headgroups; ^[10]^ or (2) salting-out effects where kosmotropic (hydrophilic) ions disrupt the surfactant hydration shells, enhancing $\text{UO}_{\text{2}}^{\text{2+}}$-CMPO coordination.^[11]^

For the SCH-D_2_EHPA system, Fe^3+^ yields exceeded 90% across 0.01~2 M HNO_3_ (**Figure S6c**), with *D* values up to four orders of magnitude higher than *n*-dodecane systems.^[12]^ These comprehensive results underscore the SCH-extraction system's consistent ability to achieve high extraction yields and superior distribution ratios across a variety of extractants and target metal ions, showcasing its high efficiency as a separation platform.

**S5. Intermolecular Interactions in the SCH-extraction Systems**

Non-bonded potential energies were compared across SCH-Zr^4+^, SCH-TODGA, and SCH-TODGA-La^3+^ systems to evaluate the influence of ions and extractants on intermolecular interactions. **Figure S16** summarizes the dominant attractive interactions observed in these systems, with repulsive forces omitted for clarity.

A comparative analysis of systems containing hydrophilic Zr^4+^ ions versus hydrophobic TODGA extractant reveals marked enhancements in both electrostatic attraction (from -9.57 eV to -20.32 eV) and van der Waals interactions (from -2.20 eV to -4.66 eV) between SDS and CTAB (**Figure 4g** vs. **Figure 5e**). This increase in attraction stems from TODGA-induced modulation of solvation dynamics; TODGA incorporation compresses the solvation shell,^[13]^ allowing closer proximity and stronger interactions between SDS and CTAB. This spatial confinement, in turn, facilitates enhanced orbital overlap and stronger intermolecular interactions. Additionally, TODGA-driven partial dehydration reduces the water shielding effect, weakening the intrinsic ion pair between SDS and CTAB,^[14]^ strengthening the SDS-CTAB coulombic attraction. This dual mechanism synergistically strengthens the SDS-CTAB association.

Adding La^3+^ to the SCH-TODGA system weakened SDS-CTAB electrostatic (from -20.32 to -7.01 eV) and van der Waals forces (from -4.66 to -1.73 eV) due to enhanced Debye shielding at higher ionic strength (Fig. 4g vs. Fig. 5i).^[15]^ Furthermore, the coordination of La^3+^ with surfactant headgroups perturbs their orderly assembly, resulting in a reduction of hydrophobic interactions between the hydrocarbon chains. These complex interplays of interactions collectively dictate the overall stability and selective performance of the SCH-extraction system in the presence of diverse metal ions.

**S6. Thermodynamic Model for Dual-Mechanism Extraction**

To quantitatively disentangle the contributions of key physical forces, the total Gibbs free energy change (Δ*G_total_​*) for the transfer of a metal ion (M^z+^) from the dilute phase to the condensed phase was decomposed into three primary components: electrostatic interactions, extractant coordination, and solvent effects. This approach allows the model to move beyond a purely empirical fit and provide a physically grounded explanation for the observed extraction behavior.

$$\begin{aligned} \Delta G_{total}=\Delta G_{elec}+\Delta G_{coord}+\Delta G_{solv}\#\left( S1 \right) \end{aligned}$$

The experimentally measurable distribution coefficient (D) is related to Δ*G_total_​* through:

$$\begin{aligned} lnD=-\frac{{\Delta G}_{total}}{RT}\#\left( S2 \right) \end{aligned}$$

Where *R* is the ideal gas constant (8.314 J·mol^-1^·K^-1^) and *T* is the absolute temperature (K).

**S6.1** **Electrostatic Free Energy (ΔG_elec_​)**

The electrostatic energy, Δ*G_elec_*​, of the model is designed to quantify the non-specific coulombic attraction between the positively charged metal ion (M^z+^) and the negatively charged interface of the condensed phase. This interaction is fundamentally governed by the ion’s valence, the interfacial charge density, and the extent of electrostatic screening from ions in the surrounding aqueous solution.

We modeled this term as being directly proportional to the square of the metal ion’s charge (*z*^2^), consistent with fundamental electrostatic principles. The interfacial charge density, which is a key factor, is captured as a function of the surfactant concentration [*SDS*], parameterized by the fitted constant *m*. The crucial effect of electrostatic screening is represented by an inverse relationship with the ionic strength of the solution, *I*, and the equation's form is based on the Debye-Hückel equation. A higher *I* leads to a more compressed electrical double layer, effectively diminishing the electrostatic attraction. This effect is parameterized by a fitting constant, *b*.

The resulting electrostatic term, therefore, accounts for the charge-driven component of the extraction process, providing a physically meaningful contribution that is evaluated independently of the specific chemical coordination.

$$\begin{aligned} \Delta G_{ele}\propto z_{M}\psi,\psi\propto z_{s}\left[ SDS \right]^{m}\to\Delta G_{ele}\propto z^{2}\left[ SDS \right]^{m}\#\left( S3 \right) \end{aligned}$$

$$\begin{aligned} \log\left( \gamma_{i} \right)=-\frac{A\left( z_{i} \right)^{2}\sqrt{I}}{1+Ba\sqrt{I}}\to\Delta G_{ele}\propto\frac{1}{1+Ba\sqrt{I}}\#\left( S4 \right) \end{aligned}$$

$$\begin{aligned} \Delta G_{ele}=-Az^{2}\frac{\left[ SDS \right]^{m}}{1+B\sqrt{I}}\#\left( S5 \right) \end{aligned}$$

Where *B* is the Debye-Hückel constant, which depends on the solvent’s dielectric constant and temperature. *A* is a constant scaling the strength of interfacial electrostatics.

**S6.2 Coordination Free Energy (ΔG_coord_​)**

The coordination energy, Δ*G_coord_*​, of the model is designed to quantify the specific chemical interactions between the metal ion and the various coordinating species present in the condensed phase. In this semi-empirical framework, 𝐾*_con_* is defined as an apparent equilibrium constant describing the coordination-related free-energy contribution.

To quantitatively capture the concentration dependencies, 𝐾*_con_* is modeled as a function of the concentrations of the key coordinating species, specifically the neutral extractant, [*L*], the surfactant [*SDS*], the inducer, [*HFIP*], and [$\text{NO}_{\text{3}}^{\text{-}}$] in accordance with the law of mass action. Among these, [*L*] and [$\text{NO}_{\text{3}}^{\text{-}}$] were retained because they are directly associated with the formation and stabilization of nitrate-metal neutral extractable species in TODGA-based systems^[16]^, whereas [*SDS*] and [*HFIP*] were included to account for their modulation of the LLPS microenvironment. The resulting coordination term, therefore, accounts for the chemically-driven component of the extraction process, allowing us to evaluate its contribution to the overall free energy of extraction.

$$\begin{aligned} \Delta G_{coord}=-RTlnK_{con}\#\left( S6 \right) \end{aligned}$$

$$\begin{aligned} K_{con}=f\left( \left[ SDS \right],\left[ HFIP \right],\left[ {NO}_{3}^{-} \right],\left[ L \right] \right)=C_{1}{\cdot\left[ SDS \right]}^{p}\cdot\left[ HFIP \right]^{q}\cdot\left[ {NO}_{3}^{-} \right]^{r}\cdot\left[ L \right]^{n}\#\left( S7 \right) \end{aligned}$$

$$\begin{aligned} \Delta G_{coord}=-RTln\left( C_{1}{\cdot\left[ SDS \right]}^{p}\cdot\left[ HFIP \right]^{q}\cdot\left[ {NO}_{3}^{-} \right]^{r}\cdot\left[ L \right]^{n} \right)\#\left( S8 \right) \end{aligned}$$

Where *C*_1_​ is a constant scaling the strength of coordination. *p*, *q*, *r*, and *n* represent the apparent concentration-dependent exponents of the concentration of SDS, HFIP, nitrate, and the extractant.

**S6.3 Solvation Free Energy (ΔG_solv​_)**

While solvent effects are a critical component of the overall free energy balance, a distinct, explicit solvation term is not included in our model. Instead, the effects of solvation and desolvation in the condensed phases are implicitly captured within the coordination term.

Specifically, the baseline constant *c* in the coordination term serves as a broad descriptor, accounting for the inherent free energy of transfer in the absence of specific interactions, including the net difference in solvation energy between the two phases. Following the Born equation,^[17]^ the Gibbs free energy of solvation for $\text{UO}_{\text{2}}^{\text{2+}}$ scales as Δ*G_solv_* ∝ z^2^. The solvation free energy was approximated as a constant term, which represents the baseline penalty or stabilization associated with transferring the hydrated metal ion into the condensed phase.

$$\begin{aligned} \Delta G_{solv}\propto z^{2}=C_{2}\#\left( S9 \right) \end{aligned}$$

By integrating these effects implicitly, our model remains parsimonious while still providing a physically meaningful representation of the key energetic drivers of the extraction. The final thermodynamic model is obtained:

$$\begin{aligned} \Delta G_{total}=-Az^{2}\frac{\left[ SDS \right]^{m}}{1+B\sqrt{I}}-RTln\left( C_{1}{\cdot\left[ SDS \right]}^{p}\cdot\left[ HFIP \right]^{q}\cdot\left[ {NO}_{3}^{-} \right]^{r}\cdot\left[ L \right]^{n} \right)+C_{2}\#\left( S10 \right) \end{aligned}$$

$$\begin{aligned} lnD=\frac{Az^{2}}{RT}\cdot\frac{\left[ SDS \right]^{m}}{1+B\sqrt{I}}+lnC+pln\left[ SDS \right]+qln\left[ HFIP \right]+rln\left[ {NO}_{3}^{-} \right]+nln\left[ L \right]\#\left( S11 \right) \end{aligned}$$

$$\begin{aligned} lnD=a\cdot\frac{\left[ \frac{SDS}{{SDS}_{med}} \right]^{m}}{1+b\sqrt{\frac{I}{I_{med}}}}+c+pln\left[ SDS \right]+qln\left[ HFIP \right]+rln\left[ {NO}_{3}^{-} \right]+nln\left[ L \right]\#\left( S12 \right) \end{aligned}$$

Where $a=\frac{Az^{2}}{RT}, b=B, c=lnC=lnC_{1}-\frac{C_{2}}{RT}$ . The model parameters (*a*, *m*, *b*, *c*, *p*, *q*, *r*, *n*) were determined by fitting the experimental data. Nonlinear least-squares fitting of the model was performed using the $\text{UO}_{\text{2}}^{\text{2+}}$ extraction dataset corresponding to Figure 4 and Figure S5b as a representative case. For each composition condition, 3 to 6 independent parallel experiments were carried out, and the measured distribution ratios were entered individually into the fitting procedure rather than being averaged prior to fitting. For clarity, the mean values and standard deviations of the corresponding distribution ratios are provided in the *Original Data*.

**S6.4. Parameter identifiability, uncertainty**

Parameter uncertainty was evaluated using 95% confidence intervals, relative standard error (Rel.SE, the ratio of the standard error to the fitted parameter value), t-statistics, and Jacobian-based sensitivity indices (**Table S4**). These metrics show that the fitted parameters exhibit clearly different levels of identifiability, indicating that the present model contains both strongly constrained and weakly constrained terms.

Among all fitted parameters, the apparent extractant stoichiometric coefficient *n* and the nitrate exponent *r* are the most robust. Both parameters are with low relative standard errors and large *t*-values, indicating that the extractant-dependent and nitrate-dependent contributions are strongly supported by the present dataset. This result confirms that [*L*] and [$\text{NO}_{\text{3}}^{\text{-}}$] are the two most statistically identifiable composition variables in Δ*G_coord_* term.

*c* and the nonlinearity exponent *m* show intermediate identifiability. Their uncertainties remain moderate, suggesting that the model captures the overall concentration dependence of Δ*G_elec_*, although the precise value of *m* should not be overinterpreted. By contrast, the *a* is larger uncertainty. This reflects a common challenge in nonlinear regression, namely, partial parameter non-identifiability caused by correlation between terms with similar functional dependence on composition variables. In the present model, *a* and *m* jointly determine the magnitude and surfactant-concentration dependence of the electrostatic contribution, and can therefore partially compensate for each other during fitting. As a result, although the model still achieves good overall regression performance (R^2^ = 0.918), the confidence interval of *a* remains relatively broad.

The weakest identifiability is observed for *p* and especially *q*. Both parameters exhibit very large relative standard errors and small *t*-values, indicating that the SDS- and HFIP-dependent terms in Δ*G_coord_* are only weakly resolved within the experimental conditions. Therefore, these parameters should not be assigned a strong mechanistic meaning. Instead, they are more appropriately regarded as secondary correction terms in the semi-empirical free-energy expression.

Overall, the present results indicate that the statistically reliable information captured by the model lies in the extractant-dependent and nitrate-dependent contributions, whereas the exact magnitudes of weaker SDS- and HFIP-related effects remain uncertain. Accordingly, the model is best used for mechanistic interpretation and qualitative trend analysis, rather than as a strictly unique quantitative parameterization of every fitted coefficient.

**S6.5. Sensitivity Analysis**

To further evaluate the robustness of the model, a local sensitivity analysis was conducted by independently perturbing each parameter (*a*, *m*, *c*, *p*, *q*, *r*, *n*) by ±10% while monitoring changes in the sum of squared errors (SSE) and the relative electrostatic, coordination and solvation free-energy contributions. As shown in **Figure S18a**, the fitted parameters exhibit clearly different sensitivity levels. Among them, the apparent extractant stoichiometric coefficient *n* produces the largest change in SSE, followed by *m* and *r*, indicating that the overall fit is most sensitive to the extractant-dependent and nitrate-dependent terms, as well as to the nonlinearity of the electrostatic contribution. By contrast, the sensitivities of *p* and *q* are negligible, which is fully consistent with their weak identifiability discussed in **Section S6.4**.

The sensitivity of the fractional free-energy contributions is likewise different (**Figure S18b**). Perturbation of *n* causes the largest change in the relative partitioning between Δ*G_elec_* and Δ*G_coord+solv_*, whereas *c*, *a*, and *r* produce smaller but still noticeable effects. In contrast, perturbation of *p* and *q* leads to essentially no change in the free-energy fractions. Importantly, even for the most sensitive parameters, the variation in the fractional free-energy contributions remains limited, staying within a relatively narrow range (<10%). This result indicates that although the quality of the numerical fit is more sensitive to certain parameters, especially *n*, the mechanistic interpretation based on the balance between electrostatic attraction, coordination, and solvation effects is not strongly altered by moderate parameter perturbation.

Taken together with the identifiability analysis, these results show that the dominant physically meaningful information contained in the model is associated mainly with the extractant-dependent (*n*) and nitrate-dependent (*r*) terms, whereas the SDS- and HFIP-dependent terms in Δ*G_coord_* contribute little to the fitted behavior within the explored composition range. Therefore, the model is robust in qualitative analysis, even though some parameters remain only weakly constrained.

**S6.6. Physical interpretation of selected fitted coefficients**

In the present semi-empirical free-energy model, the fitted coefficients should be interpreted as effective thermodynamic descriptors under the LLPS microenvironment, rather than as direct metal-ligand binding constants. This distinction is important because the model resolves the overall extraction free energy into electrostatic, coordination, and solvation contributions, and the fitted exponents therefore quantify how the concentrations of different components modulate these effective free-energy terms within the phase-separated system.

Among these parameters, *n* can be interpreted as an apparent extractant stoichiometric coefficient. Because the extractant is the principal coordinating ligand in the system, *n* describes how the coordination-related free-energy responds to the concentration of extractant. Under the present conditions, an *n* value close to 1.18 indicates an approximately 1:1.18 apparent dependence of metal ions on extractant concentration, rather than a strict stoichiometric coordination number.

By contrast, the exponents *p*, *q*, and *r* are not treated as stoichiometric coefficients. In the present model, SDS and HFIP primarily construct the phase-separation microenvironment rather than acting as direct coordinating ligands, while the nitrate term reflects the nitrate dependence of the coordination-related free-energy contribution. Accordingly, *p*, *q*, and *r* are more appropriately interpreted as apparent dependence exponents, describing how variations in SDS, HFIP, and nitrate concentration modulate the effective coordination contribution of the extractant.

For SDS, the fitted exponent *p* is slightly positive but remains close to zero, and its confidence interval spans both positive and negative values. This indicates that the explicit concentration dependence of SDS in the coordination-related free-energy term is weak within the experimental conditions. Therefore, *p* should not be overinterpreted as evidence for a definite promoting or suppressing effect of [*SDS*]. Instead, the dominant role of SDS in the present system is more reliably reflected by the Δ*G_elec_*​ term (an increase in [*SDS*] will lead to an increase in the value of the [*SDS*]^m^ term), whereas its residual contribution to Δ*G_coord_* appears to be limited.

The exponent *q*, associated with HFIP, should be interpreted with caution because the variation in *D* is weak over the explored [*HFIP*] range, making this parameter weakly identifiable. Although its fitted value is slightly positive, the confidence interval is broad, indicating that this parameter is only weakly identifiable in the current dataset. Accordingly, *q* cannot be taken as reliable evidence for a monotonic effect of HFIP on the coordination-related free energy. Rather, the present fitting suggests that the HFIP dependence in Δ*G_coord_* is not well resolved within the explored range, which is consistent with the role of HFIP primarily as a cosolvent and phase-regulating component rather than a direct ligand.

The exponent *r*, associated with nitrate concentration, is also treated as an apparent dependence exponent. In the present dataset, [$\text{NO}_{\text{3}}^{\text{-}}$] is coupled with acidity, and therefore *r* should be understood as describing the apparent nitrate dependence of the coordination-related free-energy term. Its positive fitted value indicates that increasing nitrate concentration strengthens the coordination-related driving force, which is consistent with the nitrate-assisted extraction behavior commonly observed for U(VI) in neutral oxygen-donor extractant systems.

Overall, the fitted exponents *n*, *p*, *q* and *r* should all be regarded as effective parameters in the semi-empirical model. Here, *n* can be largely explained based on the dependence of the extractant, while *p*, *q* and *r* are the apparent dependence exponents of Δ*G_coord_*​.

**Supplementary Tables**

**Table S1**. Extraction yield and distribution ratio of Nd^3+^ from previously reported literature.

| Extractant | Diluent | Distribution ratio | Extraction yield | Acidity | Reference |
| --- | --- | --- | --- | --- | --- |
| PC 88A | Escaid 110 | 20.74 | 0.94 | pH 4  (pH 2.1~5.9) | [18] |
| AliOle | Kerosene | 7.2 | 0.88 | pH 6.5  (pH 5~6.5) | [19] |
| NR_3_HA | Toluene | 1230 | 0.999 |  | [20] |
| Cyanex 272+TBP | Escaid 110 | 8.09 | 0.89 |  | [21] |
| [T66614][BA] | Toluene | 110 | 0.991 | pH 6.4  (pH 1.4~7.1) | [22] |
| TODGA | n-dodecane | 5.32 | 0.84 | 2.9 M  (0.002~6.0 M) | [8] |
| This work | H_2_O | 8420 | 0.997 | 2.9 M  (10^-5^~3.0 M) |  |

**Table S2**. Extraction yield and distribution ratio of $\text{UO}_{\text{2}}^{\text{2+}}$ from previously reported literature.

| Extractant | Diluent | Distribution ratio | Extraction yield | Acidity | Reference |
| --- | --- | --- | --- | --- | --- |
| Cyphos IL 104 | cyclohexane | 2.9 | 0.74 | pH=1 | [23] |
| CPA III | PEG-Na_2_SO_4_ | 44 | 0.978 | pH 5  (pH 5~6) | [24] |
| T2MBP | n-dodecane | 24 | 0.96 | 5 M  (0.01~8 M) | [25] |
| N1923 | Isoparaffin IP-2028 | 7.1 | 0.934 | pH 1.5 | [26] |
| [Hbet][Tf_2_N] |  | 1.8 | 0.6 | 0.08 M  (0.01~1 M) | [27] |
| CMPO | n-dodecane + isodecanol | 17.4 | 0.945 | 3 M  (0.01-6.0 M) | [9] |
| This work | H_2_O | 59.1 | 0.849 | 2 M  (10^-5^~3.0 M) |  |

**Table S3.** Extraction yield and distribution ratio of Fe^3+^ from previously reported literature.

| Extractant | Diluent | Distribution ratio | Extraction yield | Acidity | Reference |
| --- | --- | --- | --- | --- | --- |
| BTMPPA | Cyclohexane | 5.1 | 0.8413 | pH 1.8  (pH 1.25~ 2.25) | [28] |
| D_2_EHPA | Kerosene (emulsification) | 49.5 | 0.99 | pH 4  (pH 4~4.5) | [29] |
| TRPO | D80 solvent | 7.19 | 0.998 | 3 M HNO_3_ | [30] |
| D_2_EHPA | Kerosene-SDBS-Tween-20 | 95.54 | 0.9745 | pH 4.5  (pH 4~4.5) | [31] |
| Polymeric Calix[4]arene | Chloroform | 49 | 0.98 | pH 5.4  (pH 2.2~5.4) | [32] |
| D_2_EHPA | Kerosene+ TBP | 483 | 0.9979 | 0.01 M  (0.01-12.0 M) | [12] |
| This work | H_2_O | 22779 | 0.9996 | 0.01 M  (10^-5^~3.0 M) |  |

**Table S4.** Nonlinear least-squares fitted values for the parameters in the equation with fixed electrostatic contributions.

| Parameter | *a* | *m* | *c* | *p* | *q* | *r* | *n* |
| --- | --- | --- | --- | --- | --- | --- | --- |
| Value | 0.975 | 4.0 | -7.638 | 0.014 | 0.065 | 2.855 | 1.183 |
| 95% CI | [-0.960, 2.909] | [1.327, 6.673] | [-10.305, -4.972] | [-0.183, 0.210] | [-4.376, 4.506] | [2.384, 3.326] | [1.072, 1.293] |
| relative standard errors | 101.23% | 34.10% | 17.81% | 728.33% | 3474.28% | 8.41% | 4.79% |
| t-statistics | 0.988 | 2.933 | -5.615 | 0.137 | 0.029 | 11.886 | 20.897 |
| Jacobian-based sensitivity indices | 0.374 | 0.239 | 0.081 | 0.354 | 0.023 | 0.299 | 1.000 |

**Table S5.** Composition of the MD simulation boxes.

| System | DS^-^ | Na^+^ | CTA^+^ | Br^-^ | HFIP | TODGA | Zr^4+^ | La^3+^ | $\text{NO}_{\text{3}}^{\text{-}}$ | H_2_O |
| --- | --- | --- | --- | --- | --- | --- | --- | --- | --- | --- |
| SCH- Zr^4+^ | 12 | 12 | 14 | 14 | 262 | / | 10 | / | 40 | 7910 |
| SCH-TODGA | 12 | 12 | 14 | 14 | 262 | 10 | / | / | / | 7910 |
| SCH-TODGA- La^3+^ | 12 | 12 | 14 | 14 | 262 | 10 | / | 10 | 30 | 7910 |

*Box size: 10×10×10 nm^3^

**Supplementary Figures**

**
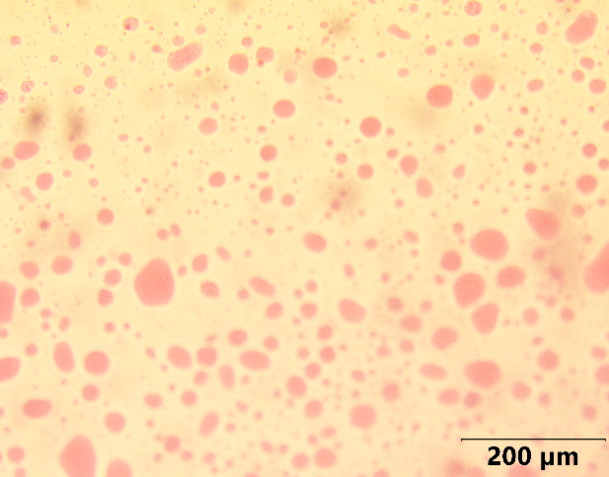

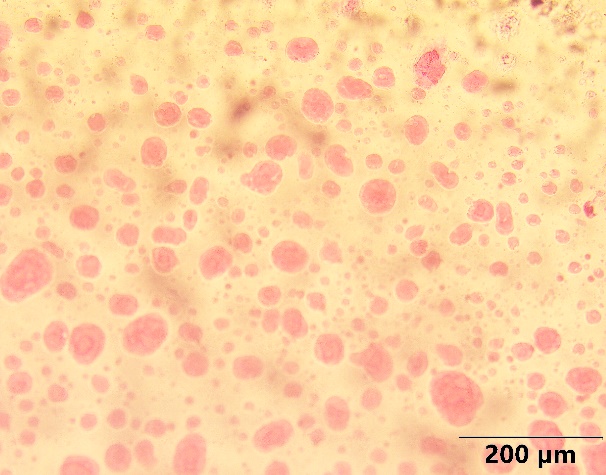
**

**Figure S1.** Optical microscope image of the SCH-extraction system (40 mM SDS, 40 mM CTAB, 4% HFIP).

**
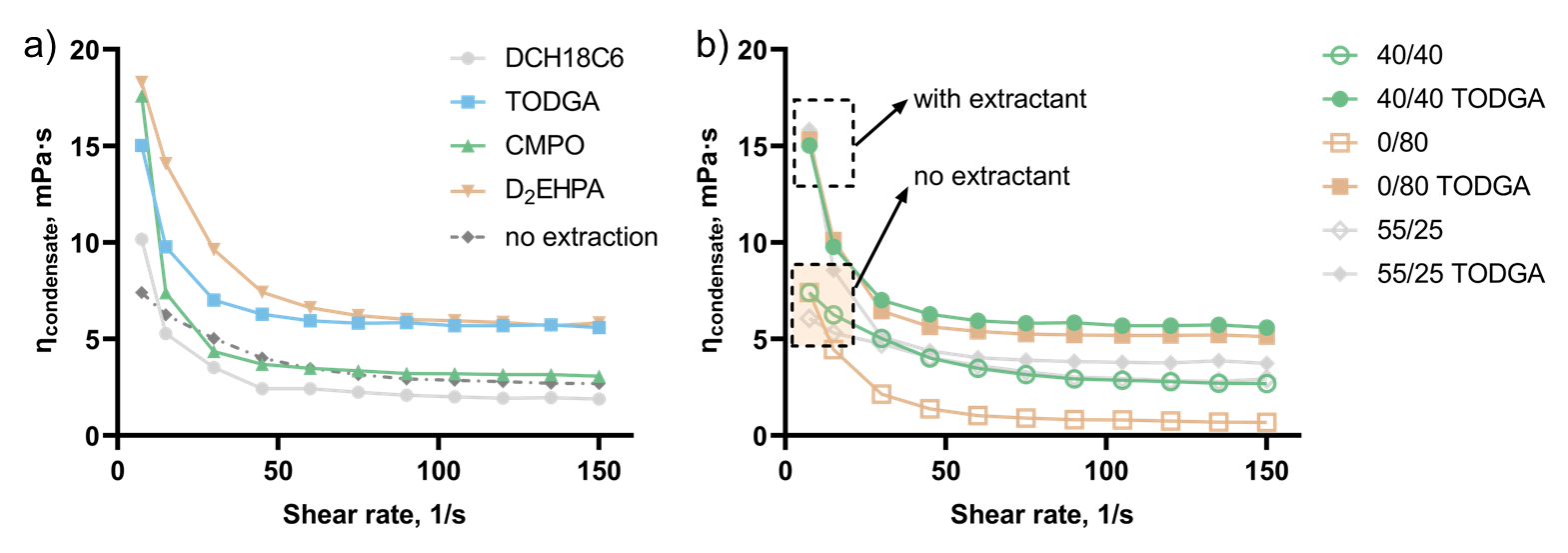
**

c)**
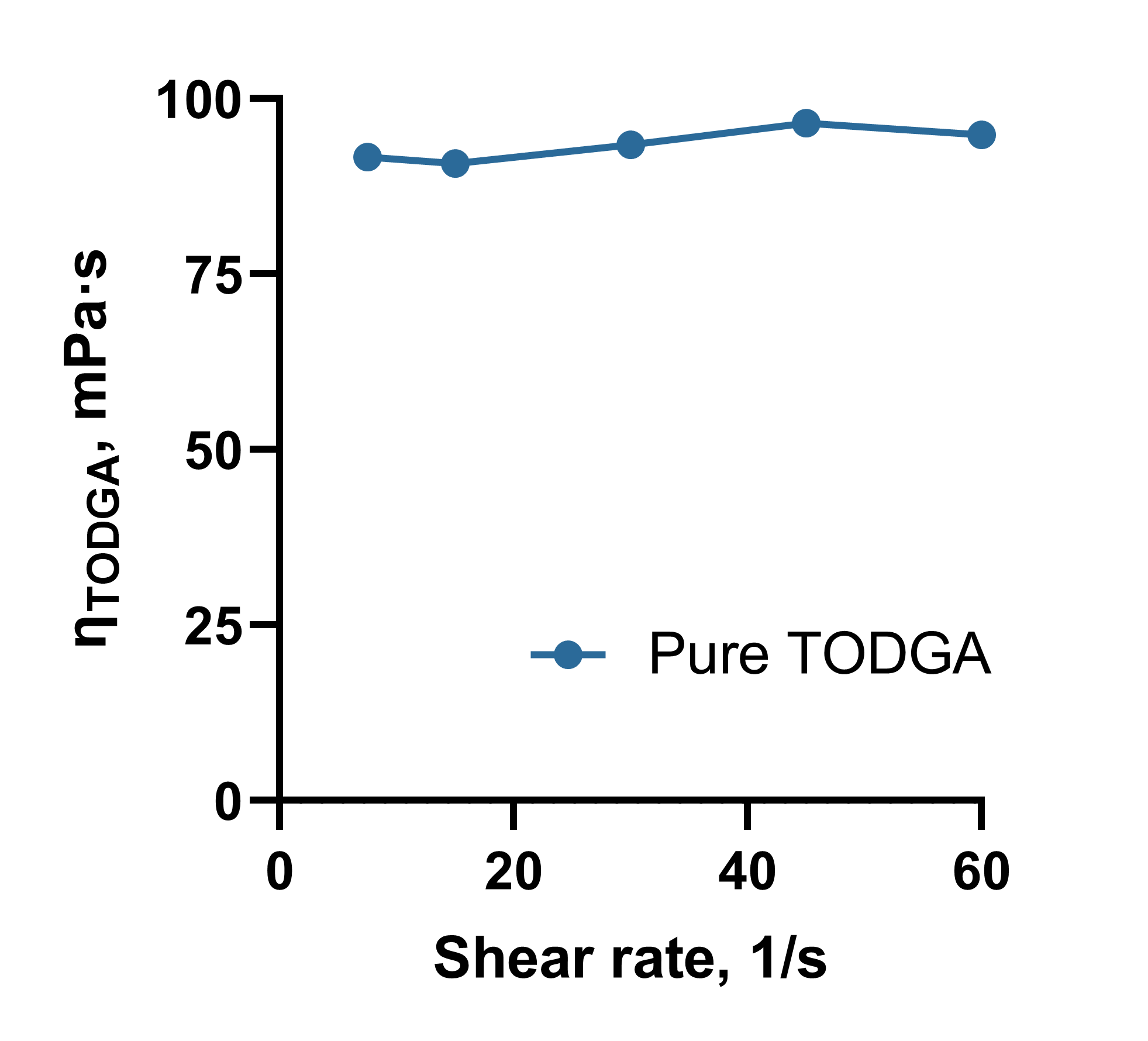
**

**Figure S2.** Apparent viscosity. a) Apparent viscosity of the SCH-extraction system encapsulating 10 mM TODGA/CMPO/D_2_EHPA. b) Effect of surfactant ratio on the apparent viscosity of the SCH-extraction system, shown with and without TODGA encapsulation (40 mM SDS:40 mM CTAB, 0 mM SDS:80 mM CTAB, 55 mM SDS:25 mM CTAB). c) TODGA.


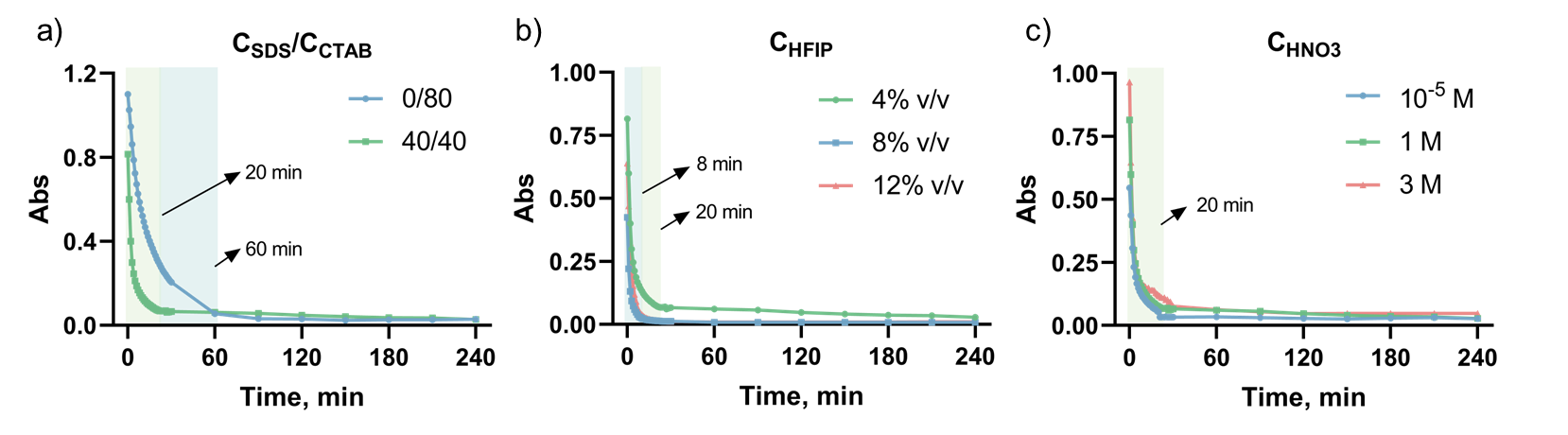


**Figure S3.** The sedimentation time of the condensed phase droplets in the SCH-extraction system. a) with 4%v/v HFIP and 1M HNO_3_ at different proportions of SDS and CTAB (*C_SDS_* : *C_CTAB_* = 40:40, 80:0); b) with *C_CTAB_* : *C_SDS_* = 40:40 and 1M HNO_3_ at different *C_HFIP_* (0.04~0.12). c) with 4%v/v HFIP and C_CTAB_ : C_SDS_ = 40:40 at different acidity (10^-5^~3 M).

**
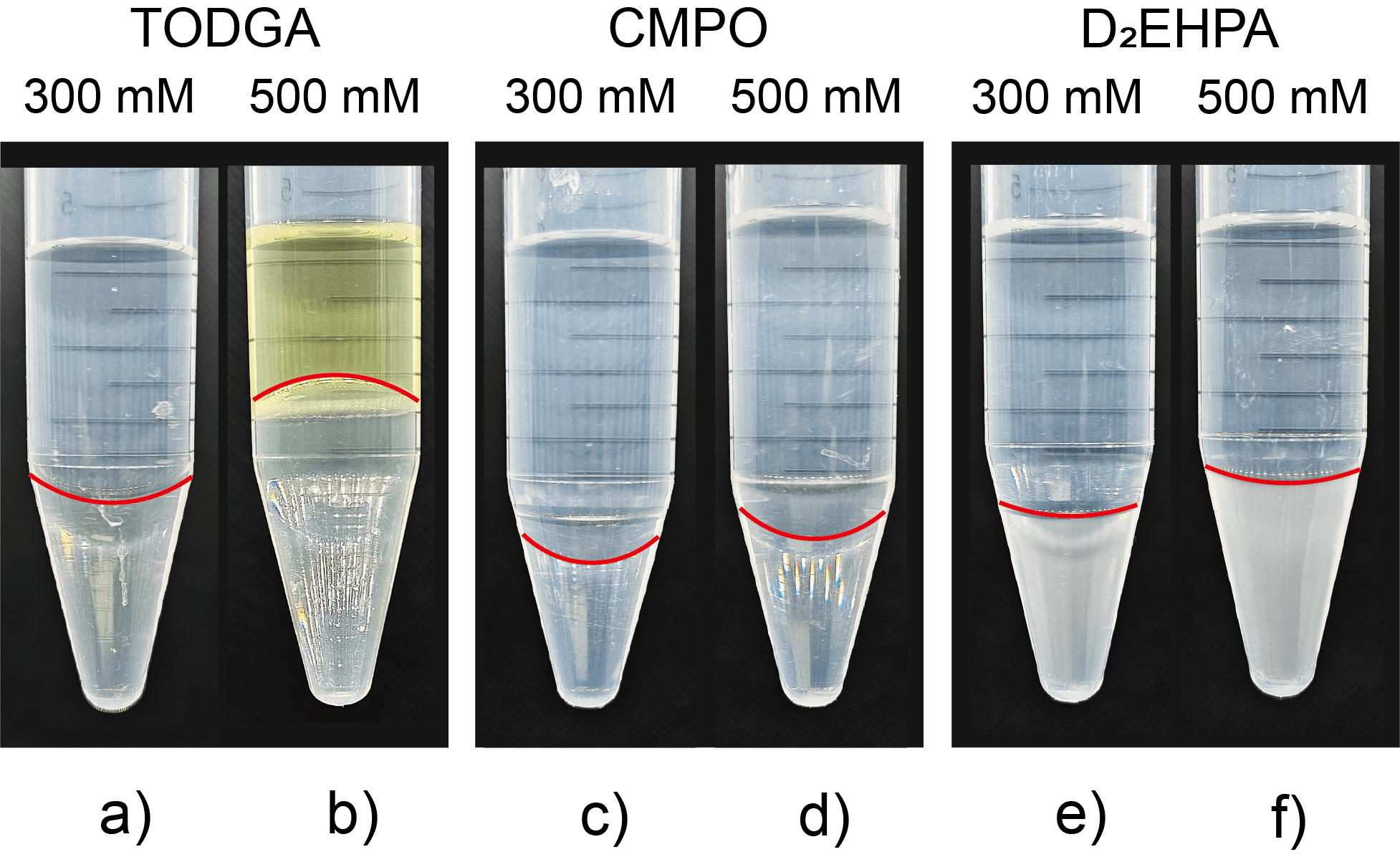
**

**Figure S4.** Visual representation of the SCH-extraction system encapsulating various commercial extractants. a,b) The SCH-extraction system containing 300 mM and 500 mM TODGA, respectively. c,d) The SCH-extraction system containing 300 mM and 500 mM CMPO, respectively. e,f) The SCH-extraction system containing 300 mM and 500 mM D_2_EHPA, respectively. Note the phase inversion observed in (b) as TODGA concentration increases to 500 mM, where TODGA (ρ_TODGA_= 0.910 g/cm^3^) becomes the dominant component of the condensed phase, leading to a decrease in its overall density.

**
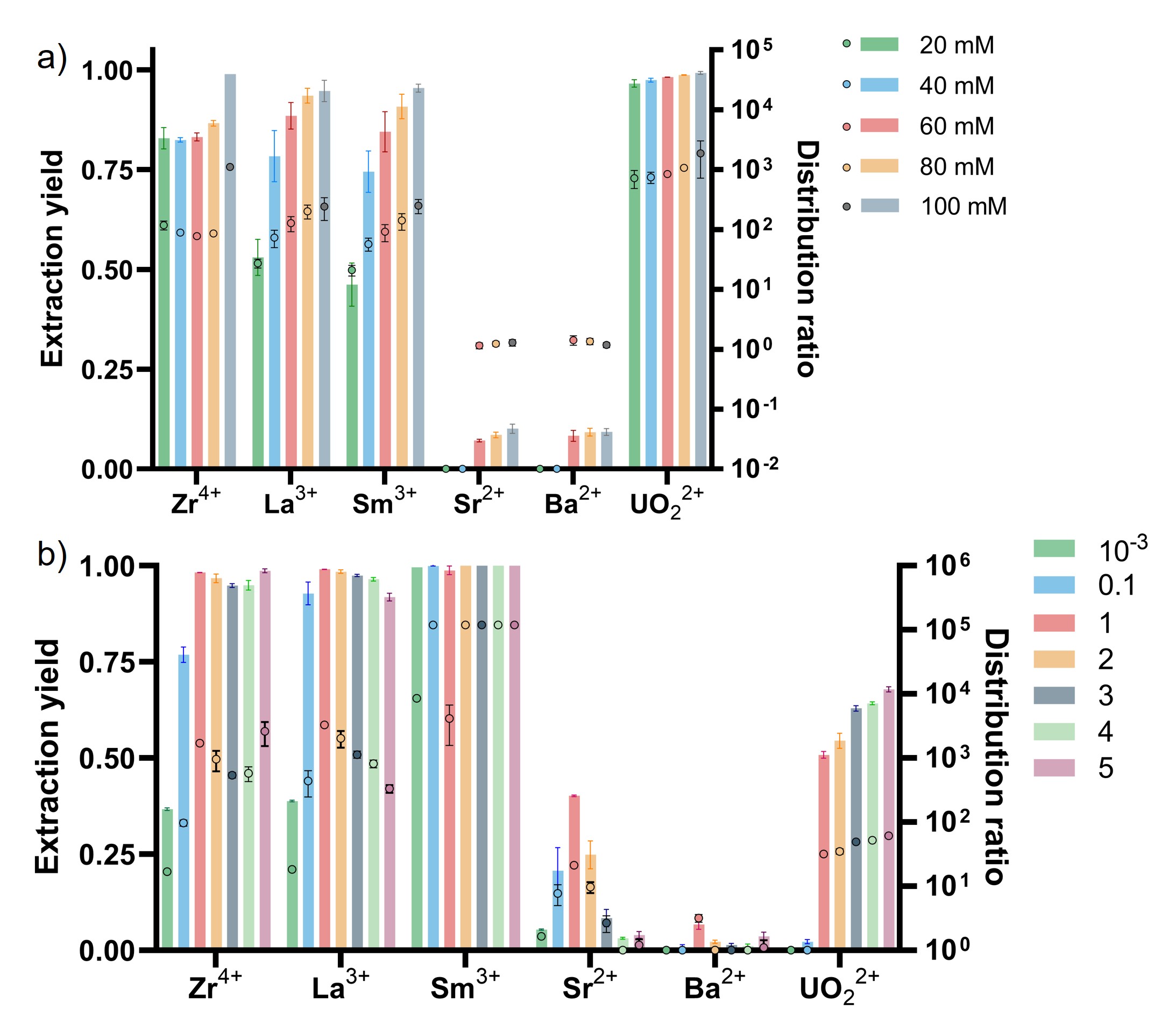
**

**Figure S5.** a) Effect of extractant concentration on the extraction yield and distribution ratio. (C_CMPO_ = 20,40,60,80,100 mM, C_SDS_ = C_CTAB_ = 40 mM, C_HFIP_ = 4 % v/v, $\text{C}_{\text{HNO}_{\text{3}}}\text{ = 1 M}$,$\text{C}_{\text{M}^{\text{n+}}}\text{ = 40 ppm}$). b) Effect of acidity on the extraction yield and distribution ratio ($\text{C}_{\text{HNO}_{\text{3}}}\text{ = }\text{10}^{\text{-3}}\text{,}\text{ 0.1, }\text{1, 2}\text{, 3, 4, 5}\text{ M}$, *C_SDS_* = *C_CTAB_* = 40 mM, *C_HFIP_* = 4 % v/v, *C_TODGA_* = 10 mM, $\text{C}_{\text{M}^{\text{n+}}}\text{ }\text{=}\text{ }\text{40 ppm}$).

**
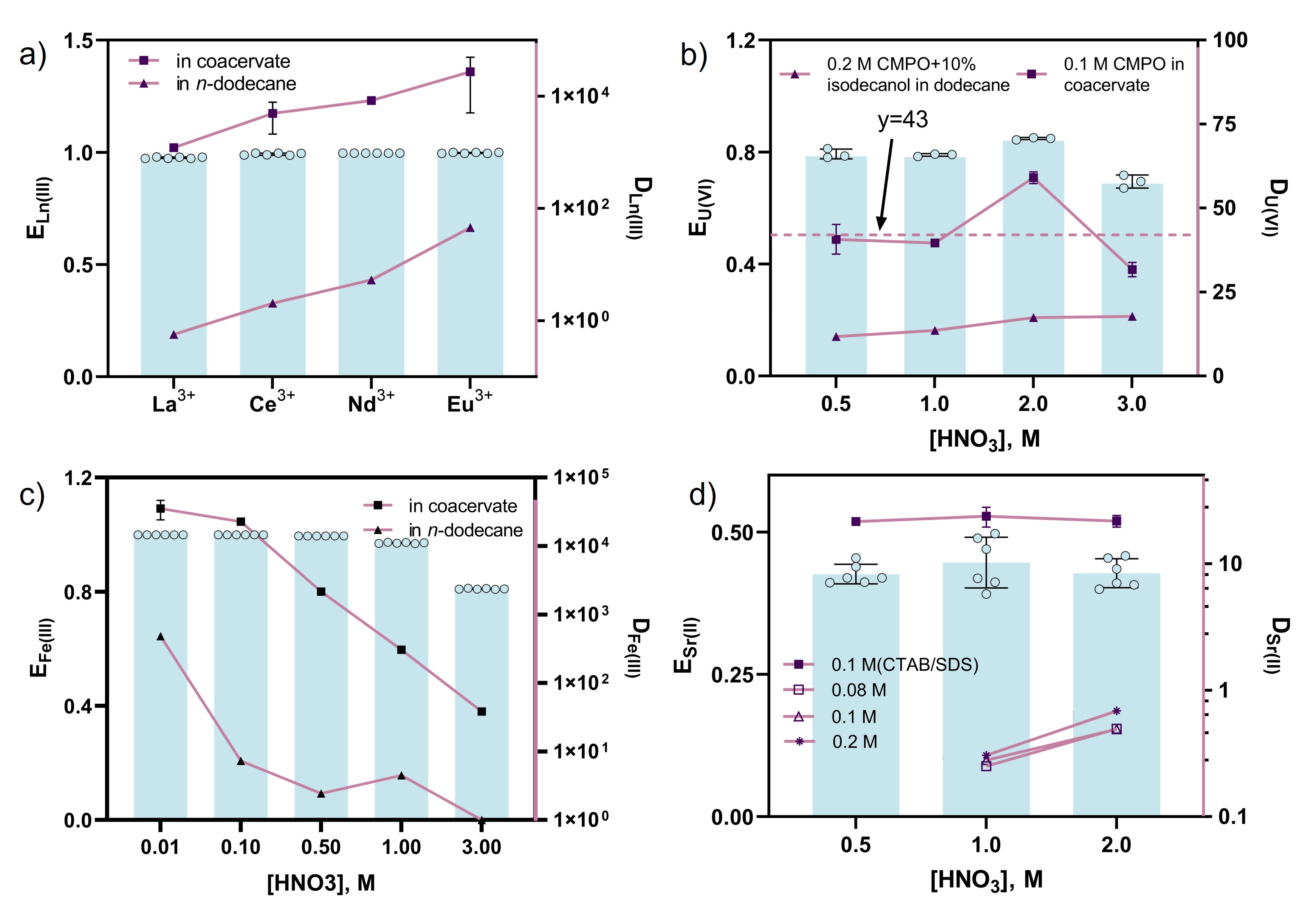
**

**Figure S6.** a) Extraction yield and distribution ratio of 100 ppm Ln^3+^ by the SCH-extraction system and the *n*-dodecane diluted system.^[8]^ C_SDS_ = C_CTAB_ = 40mM, C_HFIP_ = 4% v/v, C_TODGA_ = 10 mM, $\text{C}_{\text{HNO}_{\text{3}}}\text{ = 2.9 M}$. b) Extraction yield and distribution ratio of 2.5 ppm $\text{UO}_{\text{2}}^{\text{2+}}$ by the SCH-extraction system and the *n*-dodecane diluted system.^[9]^ C_SDS_ = C_CTAB_ = 40mM, C_HFIP_ = 4% v/v, C_CMPO_ = 100 mM, $\text{C}_{\text{HNO}_{\text{3}}}\text{ = 0.5\textasciitilde3 M}$. c) Extraction yield and distribution ratio of 500 ppm Fe^3+^ by SCH-extraction system and the *n*-dodecane diluted system.^[12]^ C_SDS_ = C_CTAB_ = 40mM, C_HFIP_ = 4% v/v,$\text{C}_{\text{D}_{\text{2}}\text{EHPA }}\text{= 100 mM}$, $\text{C}_{\text{HNO}_{\text{3}}}\text{ = 0.01\textasciitilde3 M}$. d) Extraction yield and distribution ratio of 100 ppm Sr^2+^ by the SCH-extraction system and the benzene diluted system.^[33]^ C_SDS_ = C_CTAB_ = 40mM, C_HFIP_ = 4% v/v, C_DCH18C6_ = 100 mM, $\text{C}_{\text{HNO}_{\text{3}}}\text{ = 0.5, 1, 2 M}$.

**
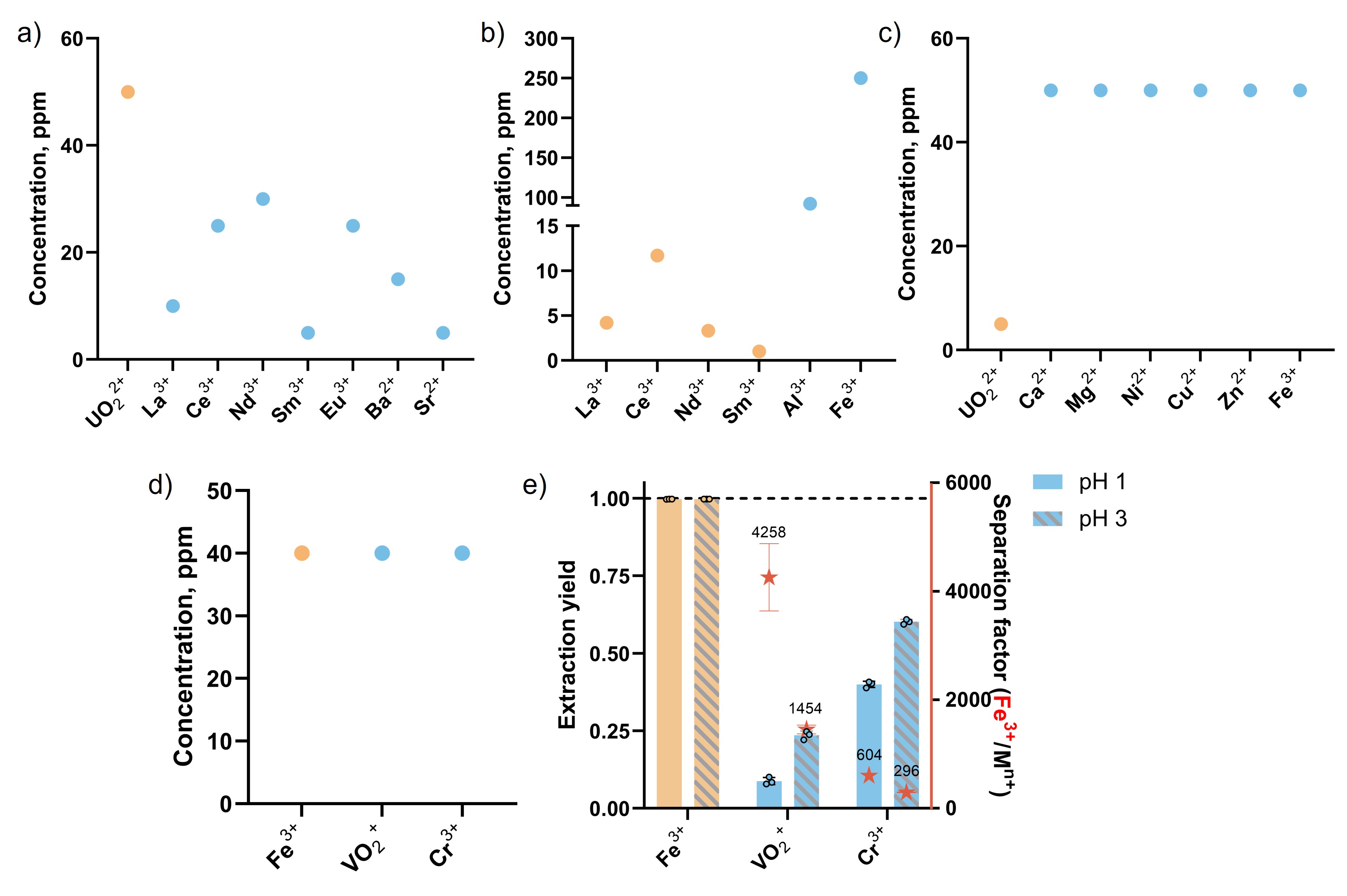
**

**Figure S7.** Application instances of simulation scenarios in the SCH-extraction system. a) The composition of simulated High-Level Liquid Waste (HLLW). b) The composition of simulated Bauxites residues. c) The composition of simulated acid uranium mining wastewater. d) The composition of simulated vanadium titano-magnetite. e) The extraction yield of Fe^3+^ and the separation factors between Fe^3+^, $\text{VO}_{\text{2}}^{\text{+}}$, and Cr^3+^ by the SCH-extraction system. C_SDS_ = C_CTAB_ = 40mM, C_HFIP_ = 4% v/v, $\text{C}_{\text{D}_{\text{2}}\text{EHPA }}\text{= 10 mM}$, $\text{C}_{\text{HNO}_{\text{3}}}\text{ = }\text{10}^{\text{-1}}\text{,}\text{10}^{\text{-3}}\text{ M}$).

**
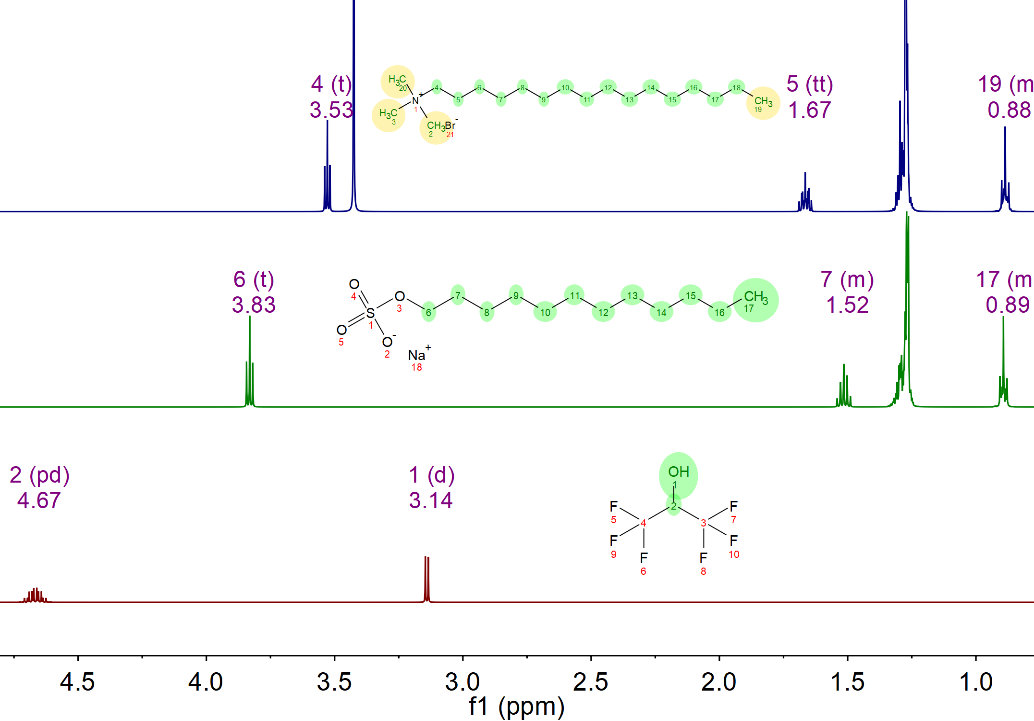
**

**Figure S8.** ^1^H nuclear magnetic resonance spectrum of the dilute phase of ^1^H NMR for SDS, CTAB and HFIP by MestReNova 12.0.

**
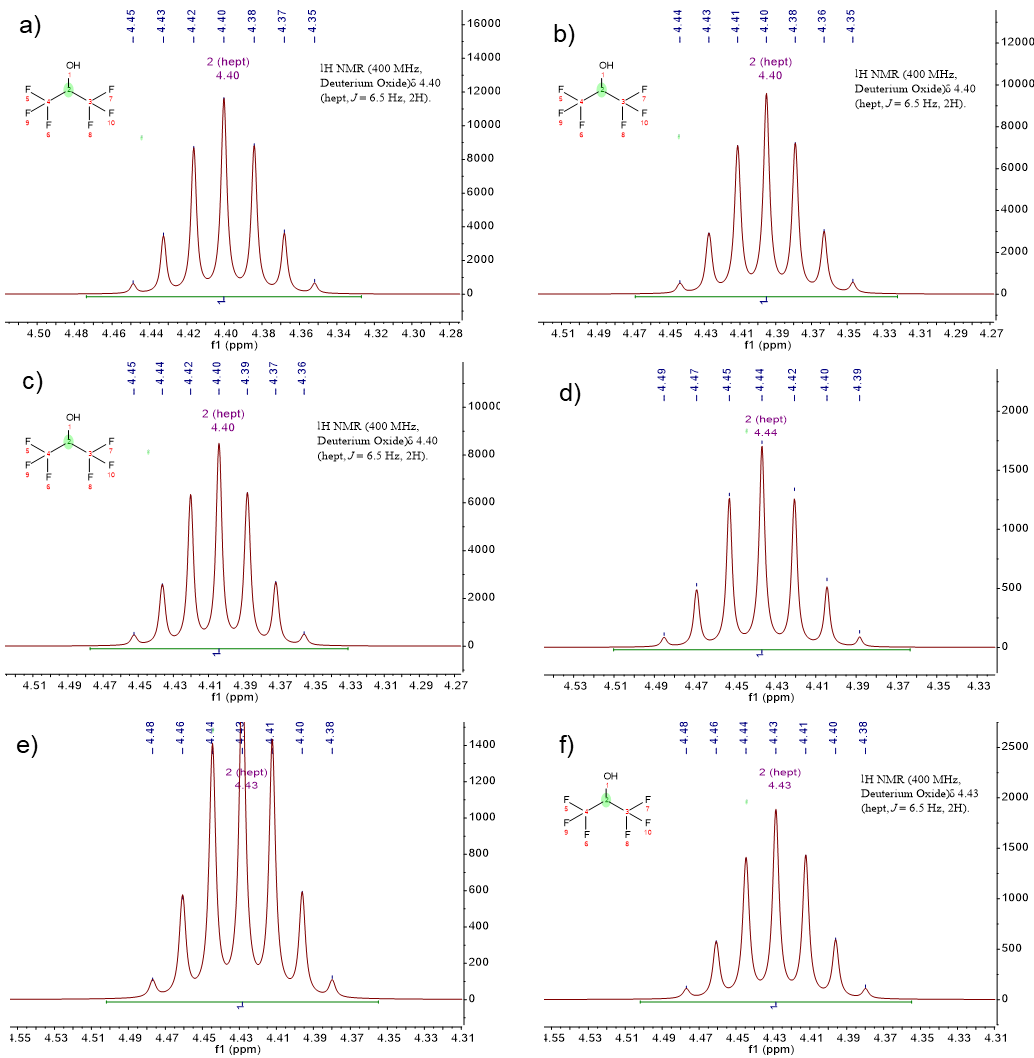
**

**Figure S9.** ^1^H nuclear magnetic resonance spectrum of the dilute phase in the SCH-extraction system encapsulating TODGA in D_2_O at 25℃. a) C_SDS_ = 110 mM, C_CTAB_ = 50 mM, C_TODGA_ = 160 mM, C_HFIP_ = 15% v/v. b) C_SDS_ = C_CTAB_ = 80 mM, C_TODGA_ = 160 mM, C_HFIP_ = 15% v/v. c) C_CTAB_ = 80 mM, C_TODGA_ = 160 mM, C_HFIP_ = 15% v/v. d) C_SDS_ = 55 mM, C_CTAB_ = 25 mM, C_TODGA_ = 80 mM, C_HFIP_ = 4% v/v. e) C_SDS_ = C_CTAB_ = 40 mM, C_TODGA_ = 80 mM, C_HFIP_ = 4% v/v. f) C_CTAB_ = 80 mM, C_TODGA_ = 80 mM, C_HFIP_ = 4% v/v.

**
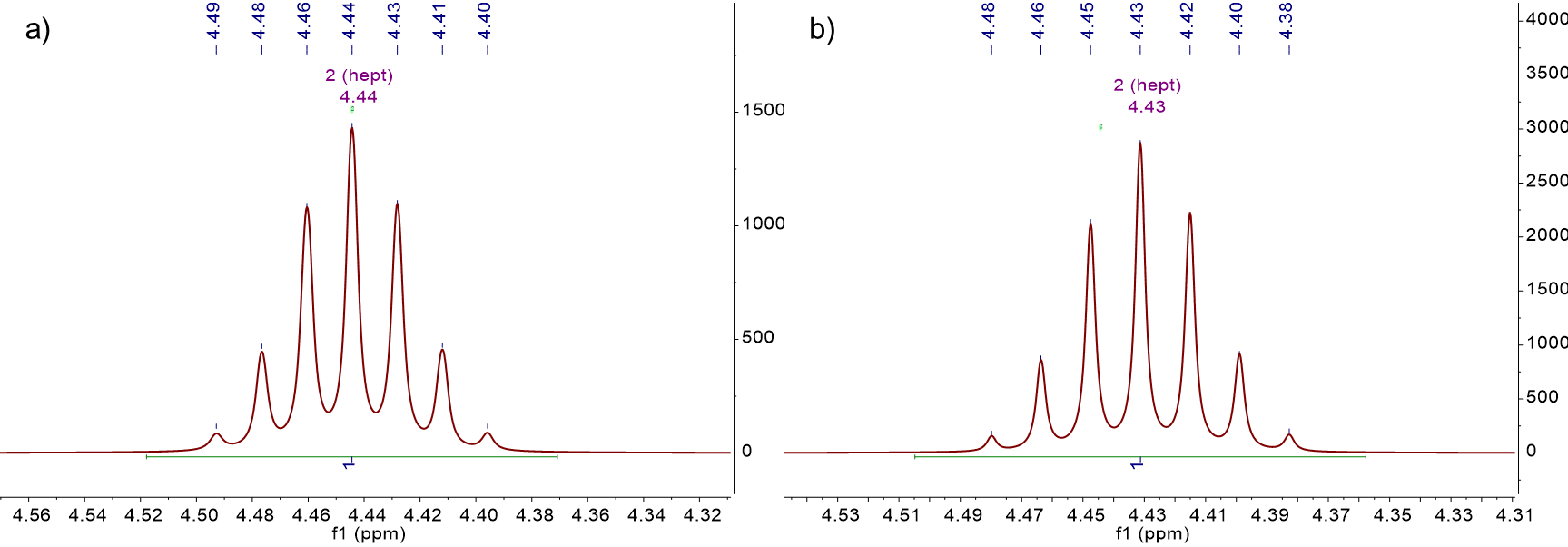
**

**Figure S10.** ^1^H nuclear magnetic resonance spectrum of the dilute phase in the SCH-extraction system encapsulating CMPO in D_2_O at 25℃. a) C_SDS_ = C_CTAB_ = 40 mM, C_CMPO_ = 80 mM, C_HFIP_ = 4% v/v. b) C_SDS_ = C_CTAB_ = 40 mM, $\text{C}_{\text{D}_{\text{2}}\text{EHPA }}\text{= 80 mM}$, C_HFIP_ = 4% v/v.

**
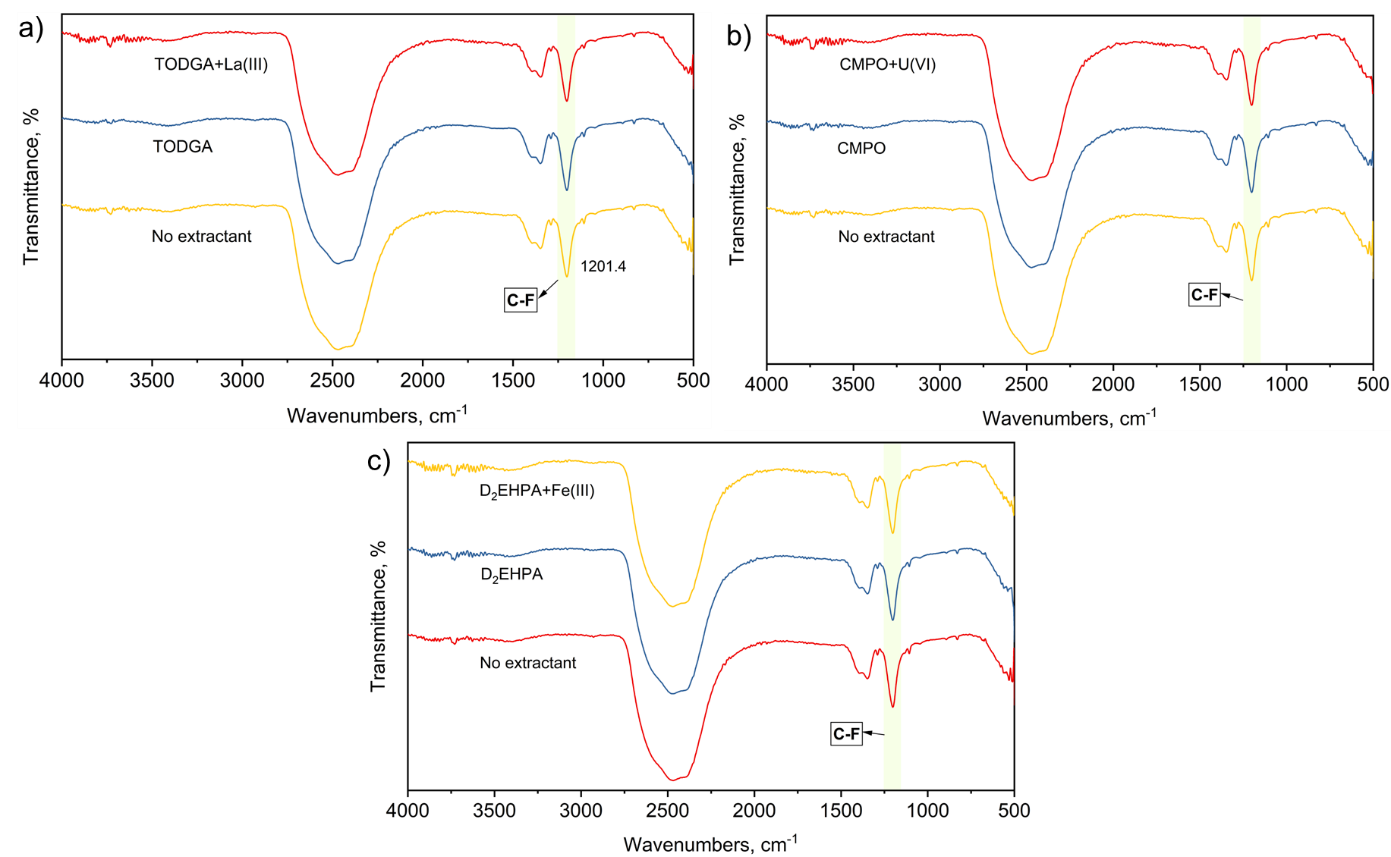
**

**Figure S11.** FT-IR spectra of the dilute phase in a) the SCH, the SCH-TODGA, and the SCH-TODGA-La^3+^ system. b) in the SCH, the SCH-CMPO, and the SCH-CMPO-$\text{UO}_{\text{2}}^{\text{2+}}$ system. c) in the SCH, the SCH-D_2_EHPA, and the SCH-D_2_EHPA-Fe^3+^ system in D_2_O. (C_SDS_ = C_CTAB_ = 40 mM, C_HFIP_ = 4 % v/v, ${\text{C}_{\text{CMPO}}\text{ = C}}_{\text{D}_{\text{2}}\text{EHPA }}\text{= 40 mM}$, $\text{C}_{\text{HNO}_{\text{3}}}\text{ = 1 M}$).

**
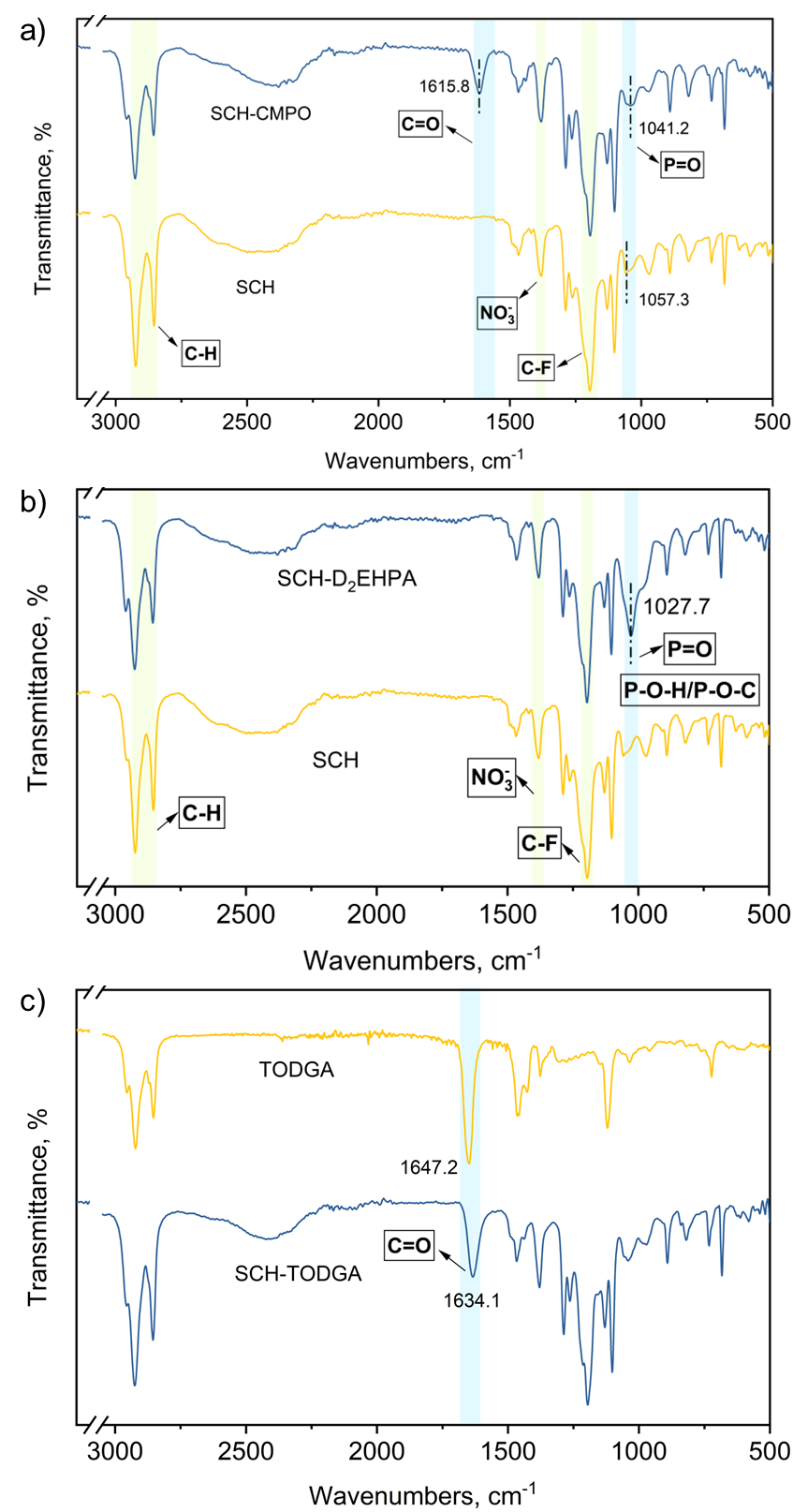
**

**Figure S12.** FT-IR spectra of the condensed phase in a) the SCH-CMPO system and b) the SCH-D_2_EHPA system in D_2_O. (C_SDS_ = C_CTAB_ = 40 mM, C_HFIP_ = 4 % v/v, ${\text{C}_{\text{CMPO}}\text{ = C}}_{\text{D}_{\text{2}}\text{EHPA }}\text{= 40 mM}$, $\text{C}_{\text{HNO}_{\text{3}}}\text{ = 1 M}$). c) FT-IR spectra of the pure TODGA and SCH-TODGA system in D_2_O of the condensed phase.

**
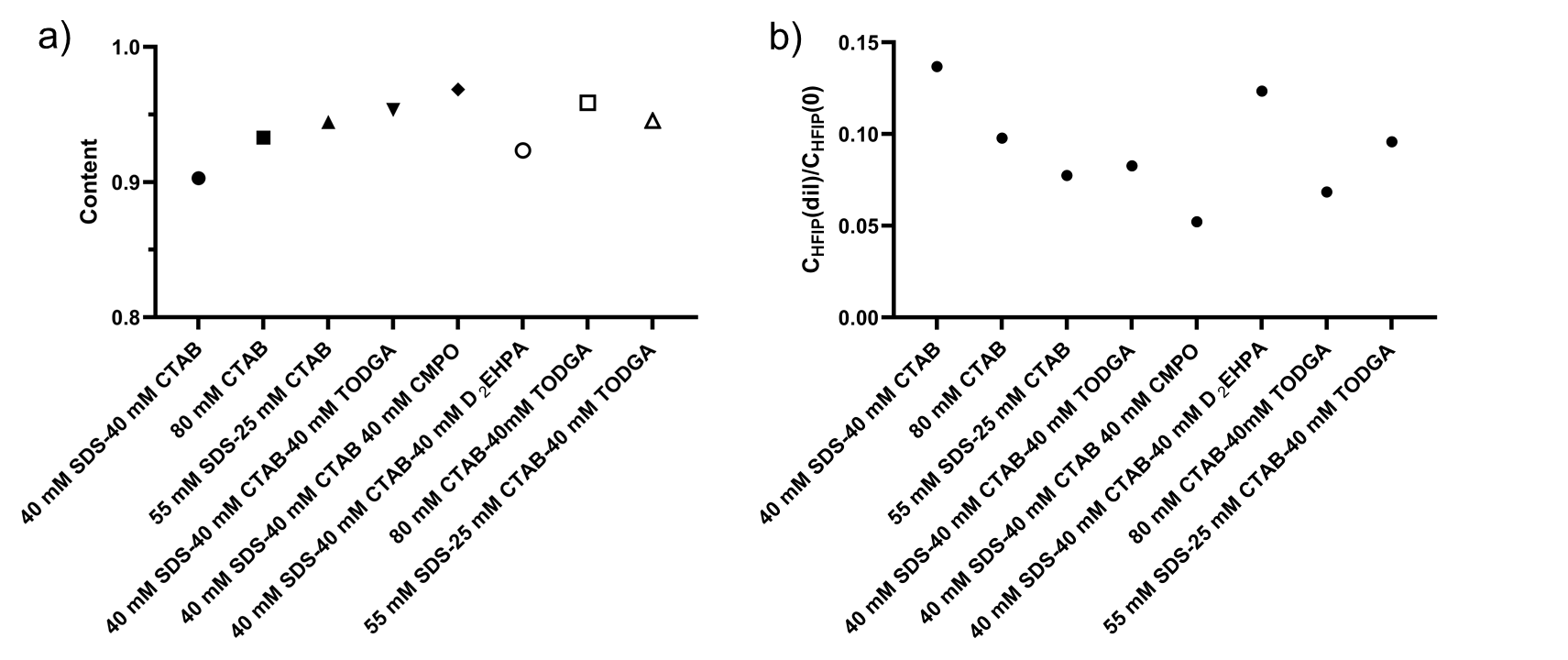
**

**Figure S13.** Total organic carbon content a) in the condensed phase. b) HFIP content in the dilute phase.

**
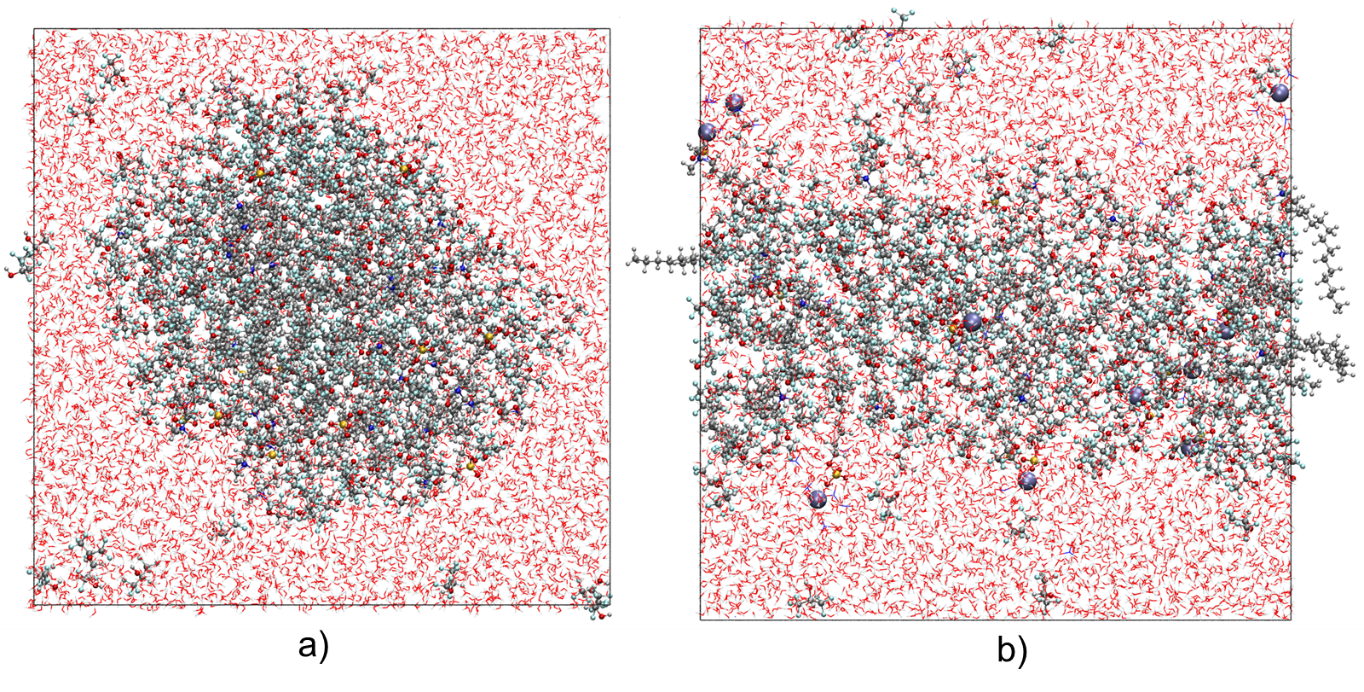
**

**Figure S14.** Representative snapshots from MD simulations. a) Intermolecular interactions among SDS, CTAB, HFIP, and TODGA in the SCH-TODGA system at 298 K and 20 ns. b) Intermolecular interactions among SDS, CTAB, HFIP, and Zr^4+^ in the SCH-Zr^4+^ system at 298 K and 20 ns. (In this picture, the purple spheres represent the La.)

**
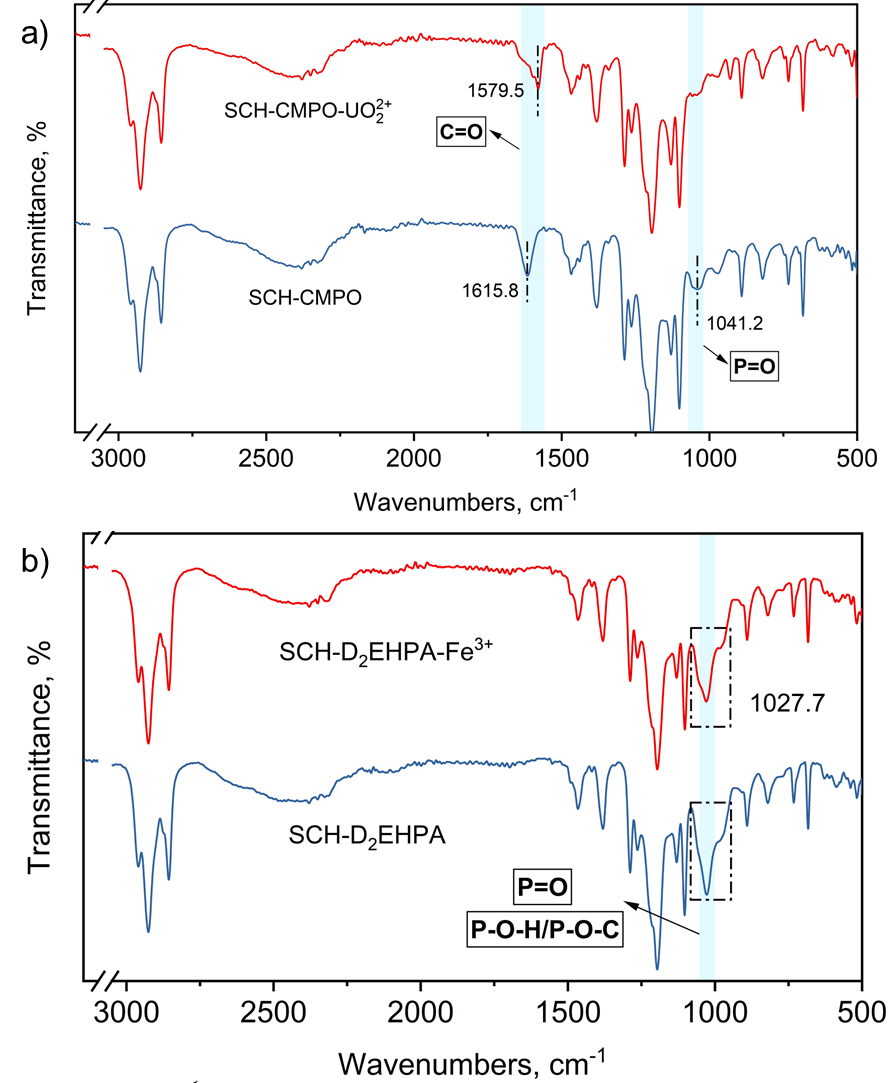
**

**Figure S15.** FT-IR spectra of the condensed phase in a) the SCH-CMPO and the SCH-CMPO-$\text{UO}_{\text{2}}^{\text{2+}}$ system and b) the SCH-D_2_EHPA and the SCH-D_2_EHPA-Fe^3+^ system in D_2_O. (C_SDS_ = C_CTAB_ = 40 mM, C_HFIP_ = 4 % v/v, ${\text{C}_{\text{CMPO}}\text{ = C}}_{\text{D}_{\text{2}}\text{EHPA }}\text{= 40 mM}$, $\text{C}_{\text{HNO}_{\text{3}}}\text{ = 1 M}$, $\text{C}_{\text{UO}_{\text{2}}^{\text{2+}}}\text{ = }\text{C}_{\text{Fe}^{\text{3+ }}}\text{= 10 mM}$).

**
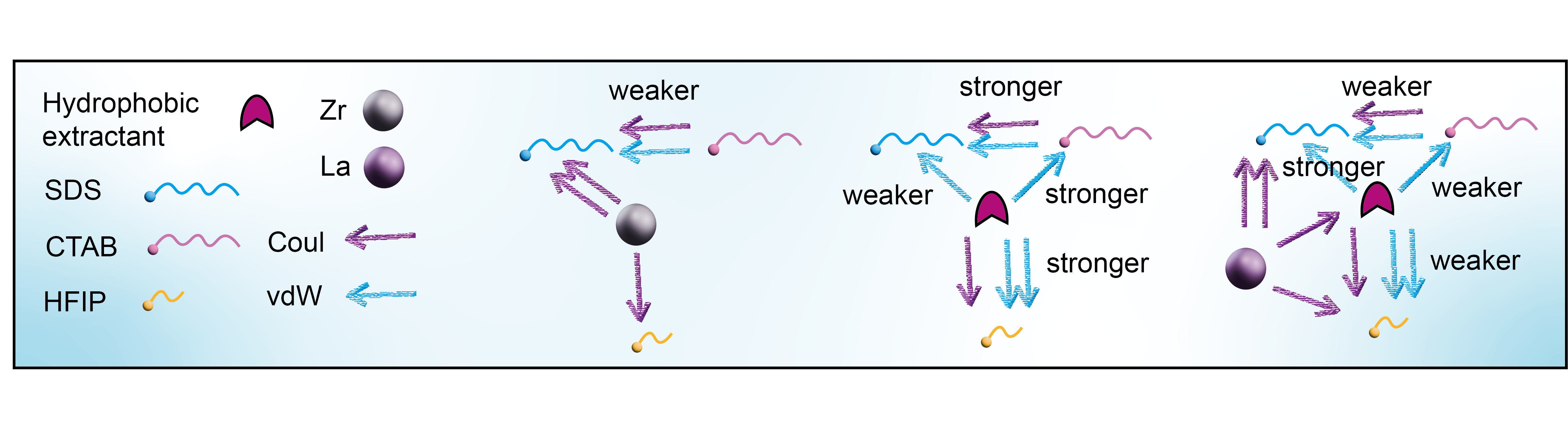
**

**Figure S16.** Comparison of intermolecular interactions between the SCH, the SCH-TODGA and the SCH-TODGA-La^3+^ systems. Purple arrows indicate electrostatic interactions, while blue arrows represent Van der Waals interactions. Repulsive forces are not shown.


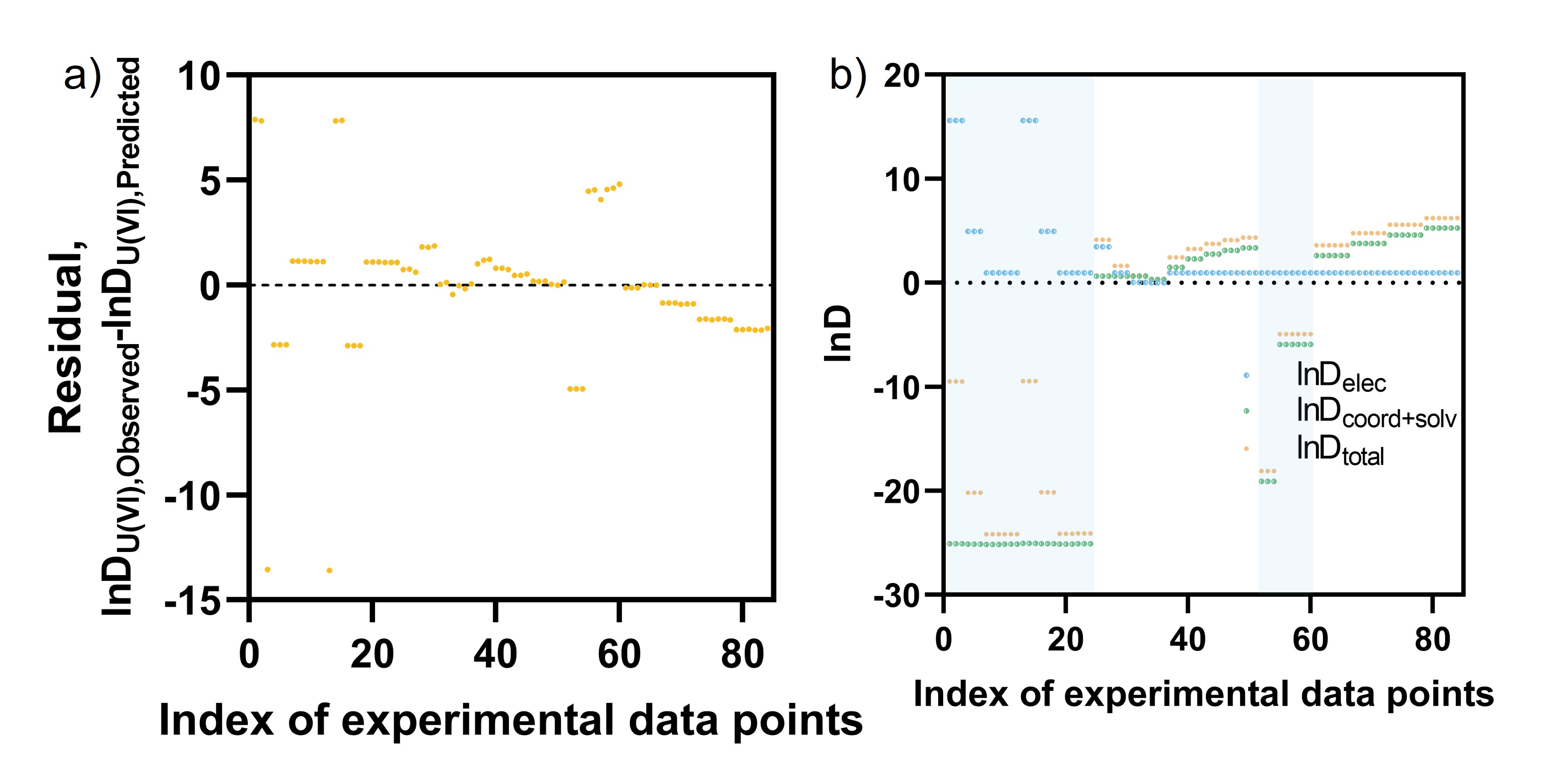


**Figure S17. a)** Residuals of the lnD values, where residual = lnD_observed_-lnD_predicted_. b) Total lnD contributions of the electrostatic, coordination and solvation free energy.


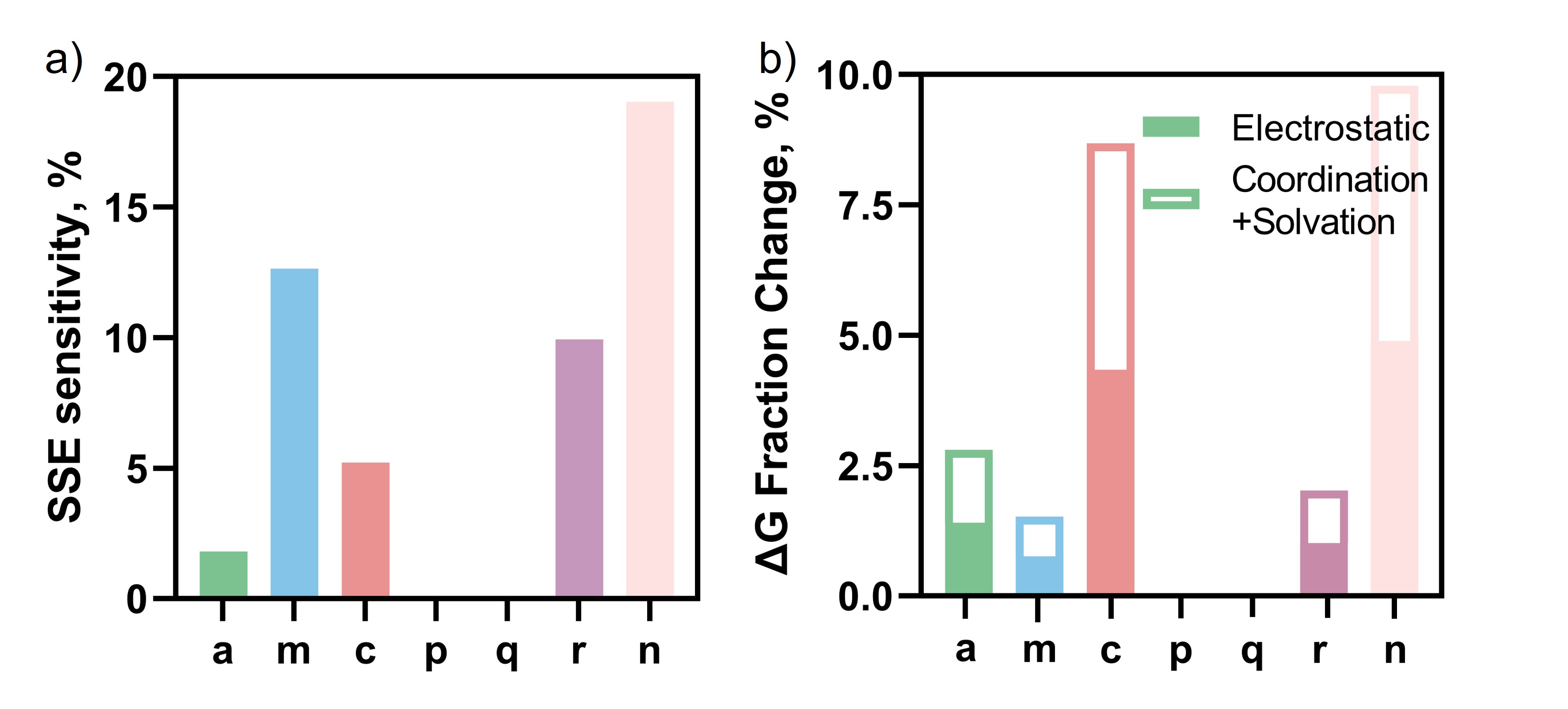


**Figure S18.** a) Parameter sensitivity of the overall model fit (±10%); b) Sensitivity of fractional free energy contributions (±10%).

*SSE refers to the sum of squares for error.

**
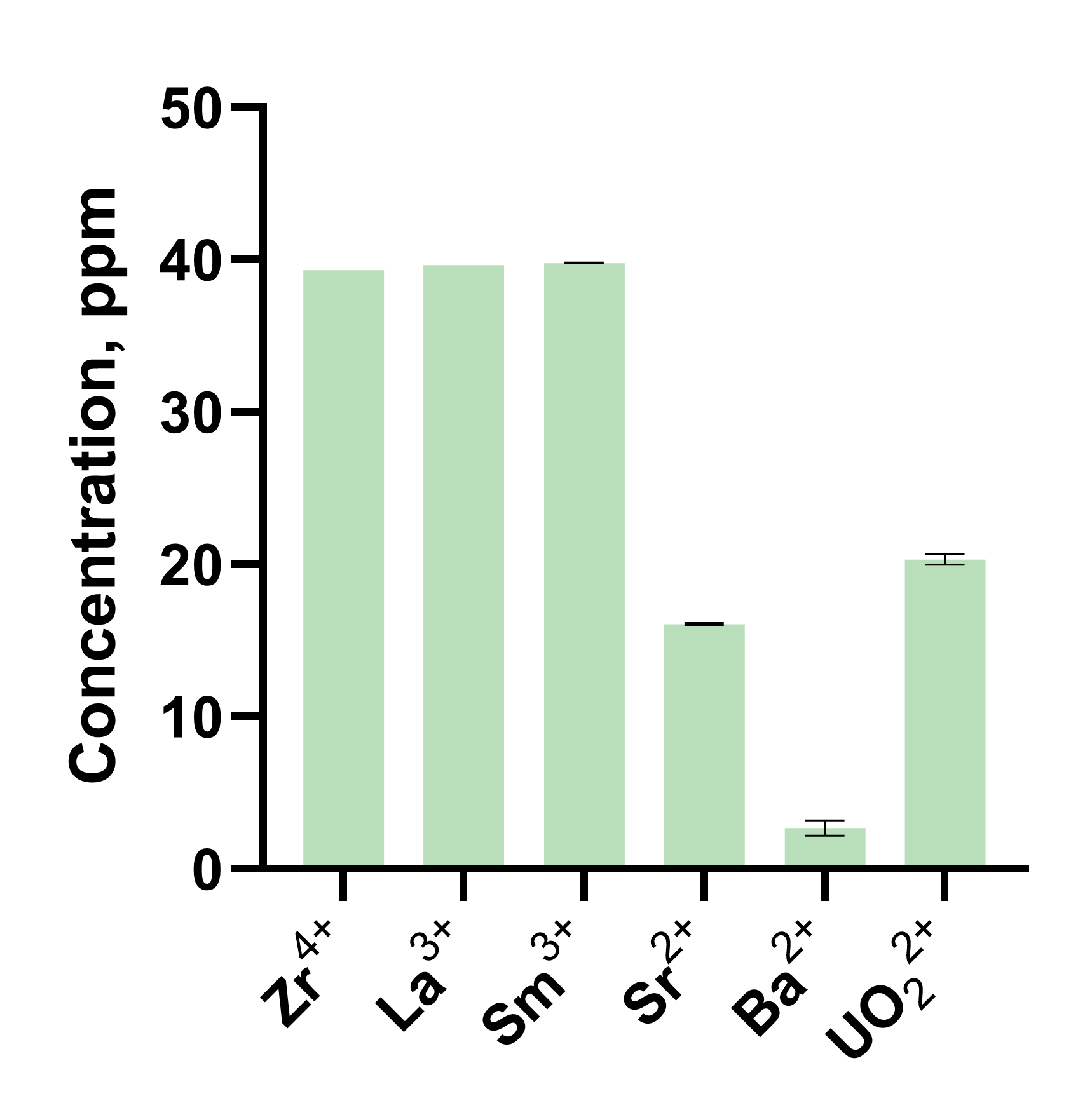
**

**Figure S19.** The concentration of metal ions transferred from the original solution into the condensed phase. The SCH-extraction system encapsulating TODGA is composed of 40 mM SDS, 40 mM CTAB, 4% v/v HFIP, 10 mM TODGA, and 40 ppm M^n+^.

**
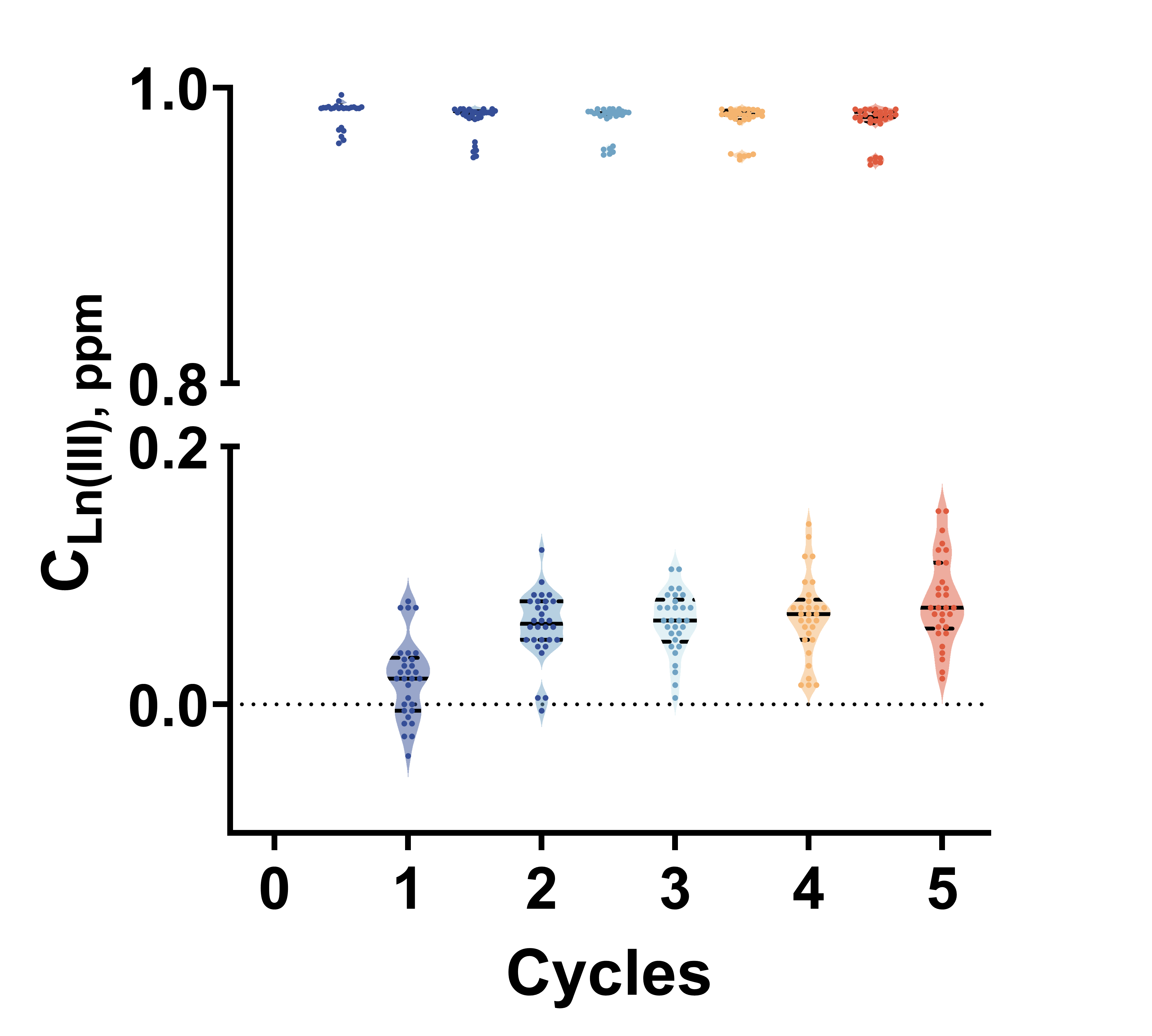
**

**Figure S20.** Five extraction-stripping cycles of Ln^3+^ in the SCH-extraction system encapsulating TODGA. Experiments were conducted with an initial Ln^3+^ concentration of 10 ppm and a solution acidity of 1 M HNO_3_. Each extraction/stripping process is separated by an interval of 24 hours.

**
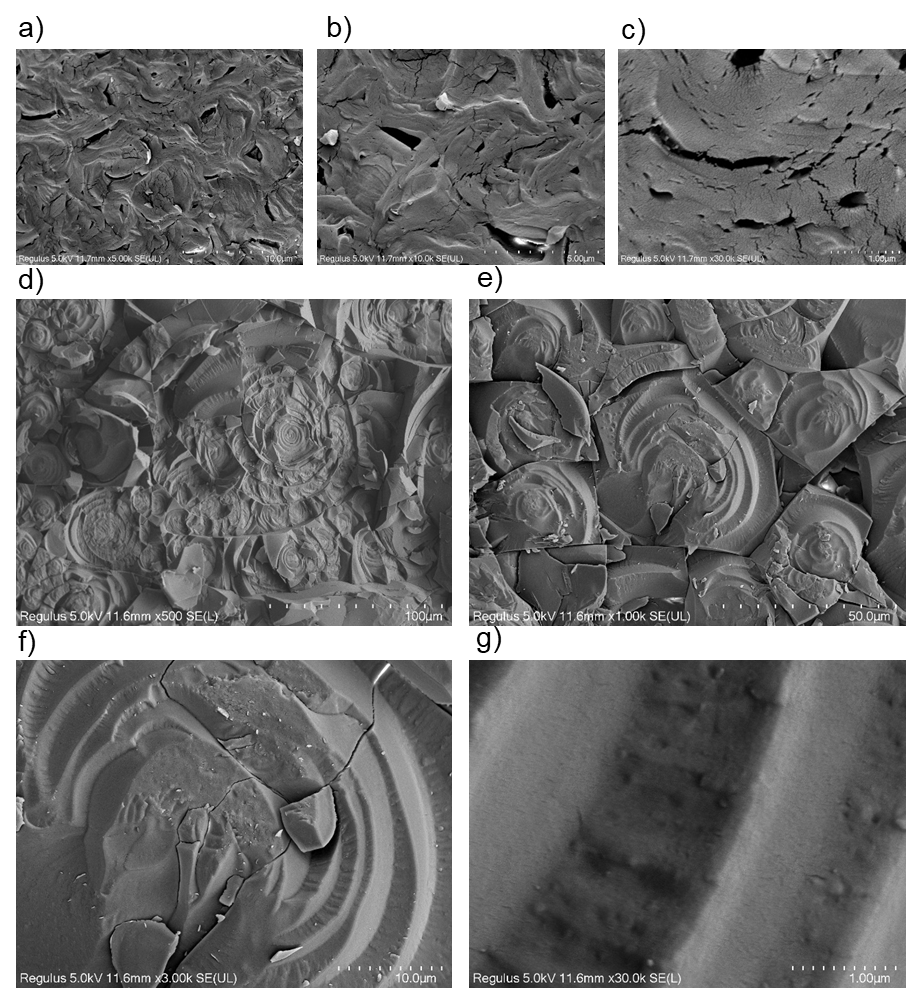
**

**Figure S21.** Cryo-SEM image of the condensed droplets in SCH-extraction system (C_SDS_ = C_CTAB_ = 40 mM, C_HFIP_ = 4 % v/v, C_TODGA_ = 100 mM, $\text{C}_{\text{HNO}_{\text{3}}}\text{ = 1 M}$). a,b,c) without extractant and, d,e,f,g) C_TODGA_ = 100 mM.

**Reference**

1. M. S. M. Wee, S. Nurhazwani, K. W. J. Tan, K. K. T. Goh, I. M. Sims, L. Matia-Merino, “Complex coacervation of an arabinogalactan-protein extracted from the Meryta sinclarii tree (puka gum) and whey protein isolate.” Food Hydrocolloids **2014**, 42, 130-138.
2. G. Q. Huang, J. X. Xiao, S. Q. Wang, H. W. Qiu, “Rheological properties of O-carboxymethyl chitosan-gum Arabic coacervates as a function of coacervation pH.” Food Hydrocolloids **2015**, 43, 436-441.
3. E. Hasanvand, A. Rafe, “Rheological and structural properties of rice bran protein-flaxseed (Linum usitatissimum L.) gum complex coacervates.” Food Hydrocolloids **2018**, 83, 296-307.
4. J. V. Gavette, I. D. Petsalakis, G. Theodorakopoulos, K.-D. Zhang, Y. Yu, J. Rebek, “The effects of hexafluoroisopropanol on guest binding by water-soluble capsule and cavitand hosts.” Chem. Commun. **2015**, 51, 17604-17606.
5. S. Bag, S. K, A. Mondal, R. Jayarajan, U. Dutta, S. Porey, R. B. Sunoj, D. Maiti, “Palladium-Catalyzed meta -C-H Allylation of Arenes: A Unique Combination of a Pyrimidine-Based Template and Hexafluoroisopropanol.” J. Am. Chem. Soc. **2020**, 142, 12453-12466.
6. H. F. Motiwala, A. M. Armaly, J. G. Cacioppo, T. C. Coombs, K. R. K. Koehn, V. M. Norwood, J. Aubé, “HFIP in organic synthesis.” Chem. Rev. **2022**, 122, 12544-12747.
7. N. Watanabe, S. Watase, N. Kadonishi, Y. Okamoto, H. Umakoshi, “Revealed properties of various self-assemblies in two catanionic surfactant systems in relation to their polarity and molecular packing state.” Langmuir **2022**, 38, 14768-14778.
8. Z.-X. Zhu, Y. Sasaki, H. Suzuki, S. Suzuki, T. Kimura, “Cumulative study on solvent extraction of elements by N,N,N′,N′-tetraoctyl-3-oxapentanediamide (TODGA) from nitric acid into n-dodecane.” Anal. Chim. Acta **2004**, 527, 163-168.
9. A. Sengupta, M. S. Murali, S. K. Thulasidas, P. K. Mohapatra, “Solvent system containing CMPO as the extractant in a diluent mixture containing n-dodecane and isodecanol for actinide partitioning runs.” Hydrometallurgy **2014**, 147-148, 228-233.
10. X. Li, K. Huang, Y. Xu, H. Liu, “Interaction of sodium and potassium ions with PEO-PPO copolymer investigated by FTIR, Raman and NMR.” Vib. Spectrosc. **2014**, 75, 59-64.
11. K. Collins, “Ions from the Hofmeister series and osmolytes: effects on proteins in solution and in the crystallization process.” Methods **2004**, 34, 300-311.
12. R. K. Biswas, D. A. Begum, “Solvent extraction of Fe^3+^ from chloride solution by D_2_EHPA in kerosene.” Hydrometallurgy **1998**, 50, 153-168.
13. S. Park, S. Kim, Y. Jho, D. S. Hwang, “Cation-π interactions and their contribution to mussel underwater adhesion studied using a surface forces apparatus: A mini-review.” Langmuir **2019**, 35, 16002-16012.
14. Y. Zhang, P. Batys, J. T. O’Neal, F. Li, M. Sammalkorpi, J. L. Lutkenhaus, “Molecular origin of the glass transition in polyelectrolyte assemblies.” ACS Cent. Sci. **2018**, 4, 638-644.
15. N. Nakatsuka, K.-A. Yang, J. M. Abendroth, K. M. Cheung, X. Xu, H. Yang, C. Zhao, B. Zhu, Y. S. Rim, Y. Yang, P. S. Weiss, M. N. Stojanović, A. M. Andrews, “Aptamer-field-effect transistors overcome Debye length limitations for small-molecule sensing.” Science **2018**, 362, 319-324.
16. A. N. Turanov, V. K. Karandashev, M. Boltoeva, C. Gaillard, V. Mazan, “Synergistic extraction of uranium(VI) with TODGA and hydrophobic ionic liquid mixtures into molecular diluent” ‌Sep. Purif. Technol. **2016**, 164, 97-106.
17. D. Bashford, D. A. Case, “Generalized Born Models of Macromolecular Solvation Effects” Annu. Rev. Phys. Chem. **2000**, 51, 129-152.
18. R. Banda, H. S. Jeon, M. S. Lee, “Separation of Nd from mixed chloride solutions with Pr by extraction with saponified PC 88A and scrubbing.” J. Ind. Eng. Chem. **2015**, 21, 436-442.
19. E. Obón, A. Fortuny, M. T. Coll, A. M. Sastre, “Experimental and modelling studies of neodymium solvent extraction from chloride media with methyl-tri(octyl/decyl)ammonium oleate ionic liquid diluted in kerosene.” Hydrometallurgy **2017**, 174, 216-226.
20. S. N. Kalyakin, V. I. Kuz’min, M. A. Mulagaleeva, “Binary extraction of neodymium nitrate using 2-ethylhexylphosphonic acid 2-ethylhexyl mono ester and tri-n-octylamine” J. Mol. Liq. **2019**, 273, 45-49.
21. R. Banda, H. Jeon, M. Lee, “Solvent extraction separation of Pr and Nd from chloride solution containing La using Cyanex 272 and its mixture with other extractants.” Sep. Purif. Technol. **2012**, 98, 481-487.
22. M. Panigrahi, M. Grabda, D. Kozak, A. Dorai, E. Shibata, J. Kawamura, T. Nakamura, “Liquid-liquid extraction of neodymium ions from aqueous solutions of NdCl_3_ by phosphonium-based ionic liquids.” Sep. Purif. Technol. **2016**, 171, 263-269.
23. J. Lu, K. He, Y. Wang, G. Chen, H. Weng, M. Lin, “An effective process for the separation of U(VI), Th(IV) from rare earth elements by using ionic liquid Cyphos IL 104.” Chin. Chem. Lett. **2022**, 33, 3422-3428.
24. Y. Huang, D. Chen, S. Chen, M. Su, Y. Chen, Yixiong Pang, G. Yuvaraja, “A green method for recovery of thallium and uranium from wastewater using polyethylene glycol and ammonium sulfate based on aqueous two-phase system.” J. Clean. Prod. **2021**, 297, 126452.
25. A. Suresh, S. Jayalakshmi, S. Sarkar, N. Sivaraman, “Effects of temperature on the extraction of U(VI) and Pu(IV) by tris(2-methylbutyl) phosphate from nitric acid media.” Radiochim. Acta **2018**, 106, 281-289.
26. N. T. Hung, L. B. Thuan, T. C. Thanh, N. T. Thuy, D. T. T. Tra, K. Do Van, M. Watanabe, P. Q. Minh, H. S. Than, N. D. Vuong, D. Van Phuc, J.-Y. Lee, J. H. Jeon, R. K. Jyothi, “Selective recovery of thorium and uranium from leach solutions of rare earth concentrates in continuous solvent extraction mode with primary amine N1923.” Hydrometallurgy **2022**, 213, 105933.
27. T. Mori, K. Takao, K. Sasaki, T. Suzuki, T. Arai, Y. Ikeda, “Homogeneous liquid-liquid extraction of U(VI) from HNO_3_ aqueous solution to betainium bis (trifluoromethylsulfonyl) imide ionic liquid and recovery of extracted U(VI).” Sep. Purif. Technol. **2015**, 155, 133-138.
28. R. K. Biswas, H. P. Singha, “Purified Cyanex 272: Its interfacial adsorption and extraction characteristics towards iron(III).” Hydrometallurgy **2006**, 82, 63-74.
29. J. H. Luo, J. Li, X. X. Duan, “Study on removal of Fe^3+^ from sodium dihydrogen phosphate by emulsification solvent extraction.” J. Ind. Eng. Chem. **2013**, 19, 727-731.
30. M. Huo, R. Zhao, Z. Ying, X. Jin, Y. Zhu, Q. Wei, X. Ren, “Efficient separation of Fe^3+^ and Cr^3+^ from chromium sludge leaching solution based on hydrogen bonding using trialkyl Phosphorus oxide.” Sep. Purif. Technol. **2025**, 364, 132462.
31. J. H. Luo, J. Li, X. X. Duan, Y. Jin, “Extraction of Fe^3+^ from sodium dihydrogen phosphate with colloidal liquid aphrons.” Ind. Eng. Chem. Res. **2013**, 52, 4306-4311.
32. H. Deligöz, M. Yilmaz, “Synthesis of polymer-supported calix[4]arenes and selective extraction of Fe^3+^.” React. Funct. Polym. **1996**, 31, 81-88.
33. K. K. Gupta, P. V. Achuthan, A. Ramanujam, J. N. Mathur, “Effect of diluents on the extraction of Sr^2+^ from HNO_3_ solutions with dicyclohexano‐18‐crown‐6.” Solvent Extr. Ion Exch. **2003**, 21, 53-71.
